# Multicomponent Hosomi–Sakurai Reaction on Isosorbide Derivatives

**DOI:** 10.3390/molecules31122155

**Published:** 2026-06-18

**Authors:** Luca Banfi, Lucia Garcia de la Parte, Chiara Lambruschini, Lisa Moni, Daniel Rufo Perez, Renata Riva

**Affiliations:** 1 Department of Chemistry and Industrial Chemistry, University of Genova, via Dodecaneso 31, 16146 Genova, Italy; chiara.lambruschini@unige.it (C.L.); lisa.moni@unige.it (L.M.); renata.riva@unige.it (R.R.); 2 Department of Organic Chemistry, University of Valladolid, Paseo de Belen, 7, 47011 Valladolid, Spain; lucia.garcia.parte@uva.es (L.G.d.l.P.); daniel.rufo@estudiantes.uva.es (D.R.P.)

**Keywords:** multicomponent reactions, Hosomi-Sakurai, isosorbide, carbohydrates, bio-based compounds, diastereoselectivity, ether synthesis

## Abstract

Trimethylsilyl ethers of monoprotected isosorbide derivatives have been subjected to multicomponent Hosomi–Sakurai reactions with allyl trimethylsilane and various aldehydes (aromatic or aliphatic) under the catalysis of trimethylsilyl triflate. This study has allowed for establishing that: (a) the best results are obtained in reactions involving TMS ethers of the *endo*-OH group; (b) the most suited protecting group is the *tert*-butyldiphenylsilyl ether. With this ideal substrate, a scope was studied using both aromatic and aliphatic aldehydes. Good yields and excellent diastereoselectivities were typically achieved with aromatic aldehydes, unless they were very encumbered at the ortho position or strongly electron-poor. With simple aliphatic enolizable aldehydes, it may be useful to use an excess of aldehydes because of self-condensation processes. These results open the way to conjugates of bio-based isosorbide with aldehyde-derived fragments, joined through a very stable ether group.

## 1. Introduction

The production of most organic compounds still heavily relies on oil. However, it is now clear that: (a) oil reserves are destined to end sooner or later; (b) it is very difficult to convert oil-based chemistry to a circular economy; and (c) the end of life of oil-derived products inexorably releases CO_2_ in the atmosphere, contributing to CO_2_ imbalance, which, in turn, is the main cause of global warming. A convenient alternative, as a carbon source, is represented by biomass, which is produced by plants using CO_2_ from the atmosphere as the carbon source [1].

In order to exploit biomass for the synthesis of fine chemicals, a completely new chemistry has to be developed, moving from the functionalization of unfunctionalized starting material (oil) to the selective handling of complex molecules. Biomass-derived building blocks are indeed often highly functionalized, rich in oxygen and stereogenic centers, and poor in nitrogen.

Multicomponent reactions (MCRs) are processes where three or more substrates are joined in a single step to give a product containing the essential parts of all substrates [2]. They are very well suited for diversity-oriented [3] or target-oriented [4] synthesis in the pharmaceutical realm, but they are also a valuable tool in green chemistry [5]. We think that application of MCRs to bio-based substrates can be a very useful contribution in the transition from oil-based to biomass-based chemistry.

For this reason, during the previous years, our group has explored the use of MCRs, mainly the Ugi or Passerini reactions, involving bio-based components [6,7].

**Scheme 1:**
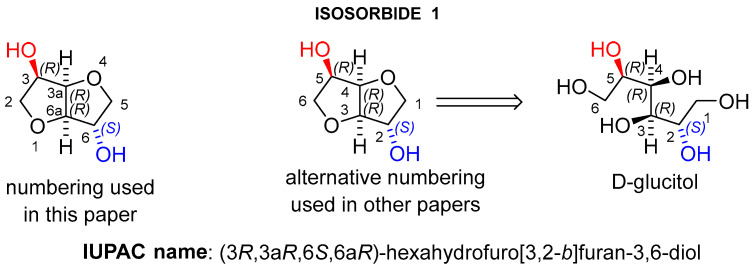
Structure, stereochemistry, and numbering of isosorbide.

Recently, we have been attracted by isosorbide **1** (Scheme 1). This dianhydrohexytol is industrially produced from sorbitol (D-glucitol), which is in turn easily obtained from D-glucose by reduction [8,9,10]. Therefore, it is a bio-based, renewable building block accessible at low cost from starch or cellulose. Its large-scale production is mainly justified by the fact that its mononitrate and dinitrate are commercial drugs. Isosorbide is also used for the fabrication of polymers, plasticizers and solvents [9,11,12].

Isosorbide is a chiral compound with four stereogenic centers that are completely retained during dehydration of sorbitol. The two hydroxy groups are diastereotopic. In the literature, there is some confusion about isosorbide numbering. We prefer to use the one, depicted in Scheme 1, that is based on IUPAC rules for the nomenclature of fused heterocycles, giving the lower series of locants to the (*R*) stereogenic centers. An alternative numbering, shown in Scheme 1, is based on the correspondence of the carbons of isosorbide to the ones of D-glucose, from which it is derived [8]. Therefore, the two diastereotopic hydroxy groups are numbered 5 and 2, instead of 3 and 6. Finally, in some papers, numbers 3 and 6 are inexplicably inverted, assigning number 3 to the (*S*) carbon [9].

Isosorbide has a rigid V-type structure that poses the 6-OH in an *exo* position and the 3-OH in an *endo* position [13]. The latter is more acidic and more reactive as a nucleophile, despite being more sterically shielded [14]. This curious fact has been attributed to a strong hydrogen bond with *O*-4 [15].

Fragments derived from isosorbide or other isohexides have been used in drug conjugates [16] or for drug delivery [17], taking advantage of their biodegradability and safety [18].

**Scheme 2:**
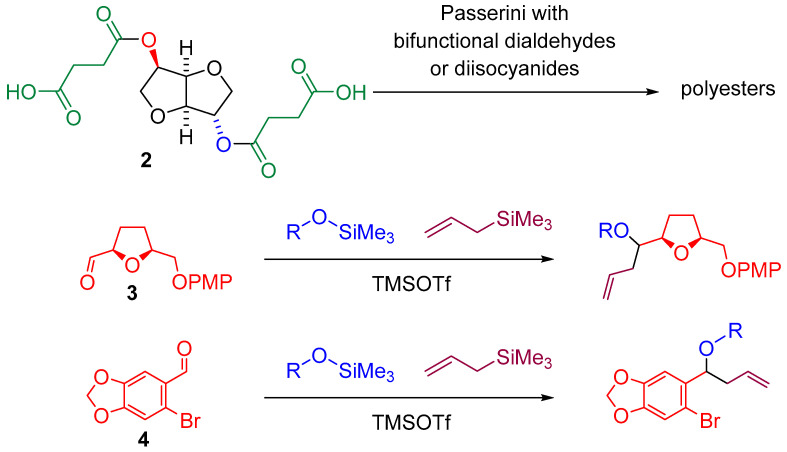
Previous published work related to this research [19,25].

We reasoned that using isosorbide as input in multicomponent reactions would be very useful for producing small libraries of conjugates that join a carbohydrate-like substructure to other aglycon moieties. To our knowledge, the only application of isosorbide-derived building blocks in an MCR (Scheme 2) employs dicarboxylic acids derived from double acylation of isosorbide. They were employed in a Passerini reaction in order to synthesize polyesters decorated with α-acyloxyamides [19].

The use of isosorbide itself in MCRs is more difficult, because the most renowned MCRs employ various functional groups (e.g., carbonyl compounds, carboxylic acids, amines, phenols, and so on), but very seldom alcohols. A notable exception is represented by the multicomponent Hosomi–Sakurai reaction (MHS), first described in 1984 [20], but then only occasionally used [21,22,23,24]. Recently, we have explored the scope of this MCR on various alcohols (mainly bio-based ones) using two aldehydes **3** and **4** (Scheme 2). The latter is an electron-rich aromatic one, whereas the former is an aliphatic, chiral, and bio-based aldehyde [25]. This study has revealed that MHS works best with TMS ethers of secondary alcohols, even encumbered ones, and that TMS ethers of chiral secondary alcohols are able, in some cases, to afford remarkable diastereoselectivity for the new generated stereogenic center.

Thus, we reasoned that monoprotected isosorbide derivatives could be good substrates for this MCR. Here, we report our results on this matter.

## 2. Results and Discussion

**Scheme 3:**
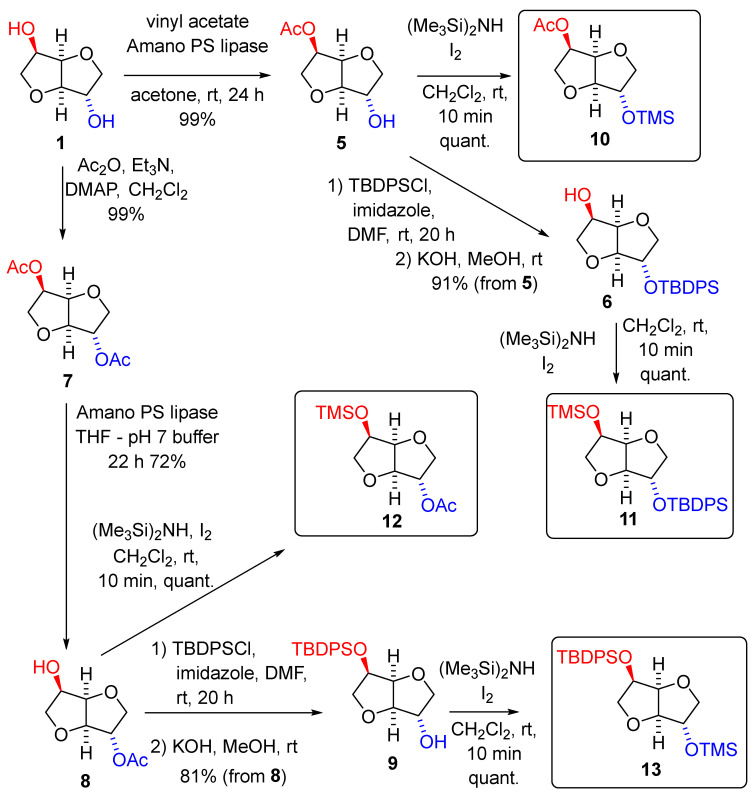
Preparation of isosorbide derivatives **10–13** as substrates for the multicomponent Hosomi–Sakurai reaction with aldehydes.

In order to differentiate the two diastereotopic hydroxy groups in isosorbide **1**, we chose to use enzymatic processes (Scheme 3) [26,27]. Although selective acetylation of the *endo* hydroxy group at position 3 is possible also by using Ac_2_O and catalytic PbO [28], the enzymatic monoacetylation is more sustainable and, using Amano PS lipase, proceeds in higher yields with complete diastereoselectivity [26]. A simple filtration of the catalyst affords pure known monoacetate **5** [26,28] with no need for further purification. In order to obtain the free alcohol at position 3, we introduced a suitable protecting group at position 6. Some protecting groups were found to be troublesome to be introduced (benzyl, *p*-metoxybenzyl) or too labile under the multicomponent reaction conditions (Me_2_*t*BuSi). The best suited one turned out to be the diphenyl-*tert*-butylsilyl group (TBDPS). Its introduction, followed by deacetylation, gave the known alcohol **6** [29] in high yields.

In order to obtain the diastereomers of **5** and **6**, that is **8** and **9**, we first converted **1** into diacetate **7** [27,30] and then performed a monohydrolysis with the same enzyme to give monoacetate **8**. Again, a very good diastereoselection was observed (98.5:1.5), but the yield (unoptimized) was slightly lower, probably caused by double hydrolysis giving water-soluble isosorbide **1**, which is then lost during extractive work-up. A large-scale production of **8** using Novozym 435 was described in the literature [27].

Monoacetate **8** was converted into mono TBDPS-protected alcohol **9** in good yields by the same procedure used for its diastereomer **5**.

Having in hand four isosorbide-derived monoalcohols, we investigated the outcome of the MHS reaction on them. However, based on our previous experience [25], we first derivatized them into the corresponding trimethylsilyl ethers **10**–**13**. Their preparation can be easily performed from the corresponding alcohols using stoichiometric (TMS)_2_NH in the presence of catalytic iodine [31]. At the end of the reaction, after destroying I_2_ with thiosulfate, TMS ethers are obtained by a quick filtration through a silica plug and evaporation to dryness. They are pure enough to be used for the MCR without further purification. In particular, TMS ether **11** (the one that we used more often in this work) was found to be very stable and can be stored at –20 °C for months.

**Scheme 4:**
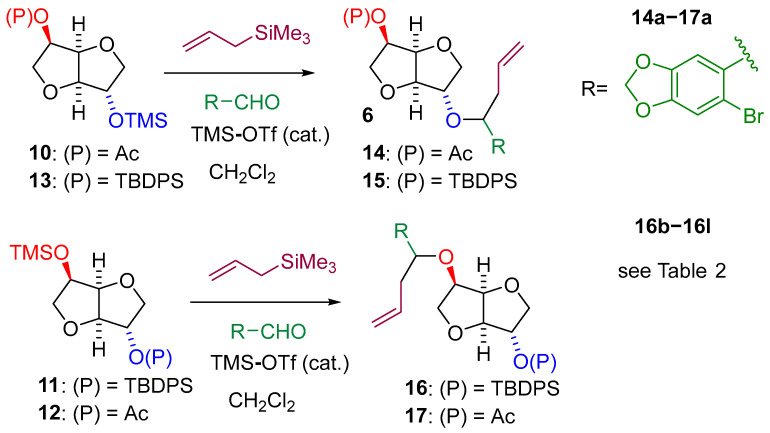
Multicomponent Hosomi–Sakurai reaction on isosorbide derivatives **10**–**13**.

The four TMS ethers were first tested on 5-bromopiperonal as a model aldehyde, and the results are listed in Table 1 (see also Scheme 4). In our previous work, we used 1.5 equivalents of TMS ethers, with the aldehyde as the limiting agent, in order to suppress bicomponent reactions [25]. In this case we decided to use a slightly lower excess (1.4 equivalents) of TMS ethers **10**–**13**.

As it is clear from Table 1, the two derivatives having the free OH group at position 3 (*endo* position) gave better results, both in terms of yields and of diastereoselectivity (entries 2 and 3). This is in line with the known higher reactivity of the *endo* hydroxy group. Moreover, the *endo* group is also considered more sterically shielded [14], and this can account for the higher diastereoselectivity. A possible rationalization of stereoselectivity in the case of *endo* TMS ethers **11** and **12** can be found in the Supplementary Materials.

On the basis of these preliminary results, we selected TMS ether **11** as the best candidate for investigating the scope of the reaction. We preferred **11** compared to **13** because of the higher yields, probably due to a partial lability of the acetoxy group under reaction conditions. In our previous work on the multicomponent Hosomi–Sakurai reaction, we have indeed observed some lability of a butyrate ester [25]. It should be noted that the related *tert*-butyldimethylsilyl protecting group was found to be non-compatible with the conditions of this MCR, undergoing extensive deprotection.

First we used a series of aromatic or heteroaromatic aldehydes (Scheme 4 and Table 2, entries 1–10).

In most cases, the diastereoselectivity was excellent (5:1 ≤ d.r ≤ 12:1) (entries 1, 3–8), while only for trimethoxybenzaldehyde it was moderate (≈3:1) (entry 2). The yield was from good to excellent for products **16a**, **16b**, **16c**, **16g**, and **16h**. With the very bulky aldehydes 2-chloro-6-fluorobenzaldehyde (entry 5) and 2-benzyloxybenzaldehyde (entry 9), significant amounts of the bicomponent product, the homoallylic alcohol derived from nucleophilic addition of the allyl group to the aldehyde, were detected. This is reasonable, because the tricomponent reaction has higher steric requirements than the bicomponent one. Also, the very electron-poor 4-nitrobenzaldehyde (entry 6) was less efficient. This was expected since the mechanism involves the intermediate formation of an oxycarbenium ion. For a detailed explanation of the mechanism, we suggest looking at our previous paper [25]. Nevertheless, the desired tricomponent product was obtained in reasonable yield also in these critical cases and could be isolated in pure form. Finally, in the case of benzofuran-2-carboxyaldehyde, the reaction was rather dirty, probably because of instability of the furan ring under these conditions, and we were not able to isolate the desired product in acceptable purity.

We also investigated two aliphatic aldehydes. With cyclohexanecarboxaldehyde, we obtained a moderate yield and, surprisingly, a decrease in the diastereoselection (entry 11). With isovaleraldehyde, the d.r. returned very good, but the yield was only fair (entry 12). However, we noticed that the reaction was indeed very clean and that the unreacted isosorbide-derived substrate (**11**) was nearly completely recovered (as the alcohol **6,** since the TMS ether does not survive chromatography).

By examination of the crude product, we reasoned that the aldehyde (which is the limiting agent under conditions A) is consumed by a side reaction. However, the bicomponent product was in this case only negligible, whereas we noticed the presence of self-condensation products. It is worth noting that self-condensation is not possible for aromatic, non-enolizable aldehydes, whereas enolization is expected to be more difficult for cyclohexanecarboxyaldehyde compared to isovaleraldehyde.

Thus, we decided to use TMS ether **11** as the limiting agent (entry 13), with a slight excess of aldehyde. The yield was not improved, but again, the overall recovery was good, as the yield was calculated based on not recovered **6**/**11**. Reasoning that also the catalyst could be consumed by conversion of the aldehyde into the corresponding silyl enol ether, we increased the equivalents of both aldehyde and TMS-OTf (adding both in two aliquots). This change brought about a significant increase in the yield (entry 14). These outcomes proved that this multicomponent Hosomi–Sakurai reaction on aliphatic aldehydes is still efficient but is in some cases in competition with self-condensation processes, requiring a different stoichiometry.

Summarizing the scope and limitations of the reaction with TMS ether **11**, easily prepared from alcohol **6**, we may conclude that

(a) The MHS reaction of TMS ether **11**, easily prepared from alcohol **6**, with aromatic, not enolizable aldehydes proceeds typically well with good to excellent diastereoselectivities. Lower, but still acceptable, yields are achieved only with electron-poor aldehydes and/or aldehydes with bulky *ortho* substituents.
(b) With aliphatic enolizable aldehydes, self-condensation processes may consume the aldehyde component, especially when the α-carbon is a methylene group. Thus, even if the reaction is quite clean and the overall recovery high, using equimolar amounts of TMS ether and of the aldehyde (or a slight excess of TMS ether) can lead to insufficient conversions. This problem may be solved, with simple aldehydes such as isovaleraldehyde, simply by using TMS ether **11** as the limiting agent and a slight excess of the aldehyde. Moreover, also an increase of the catalyst, adding it in two aliquots, may be useful.

**Scheme 5:**
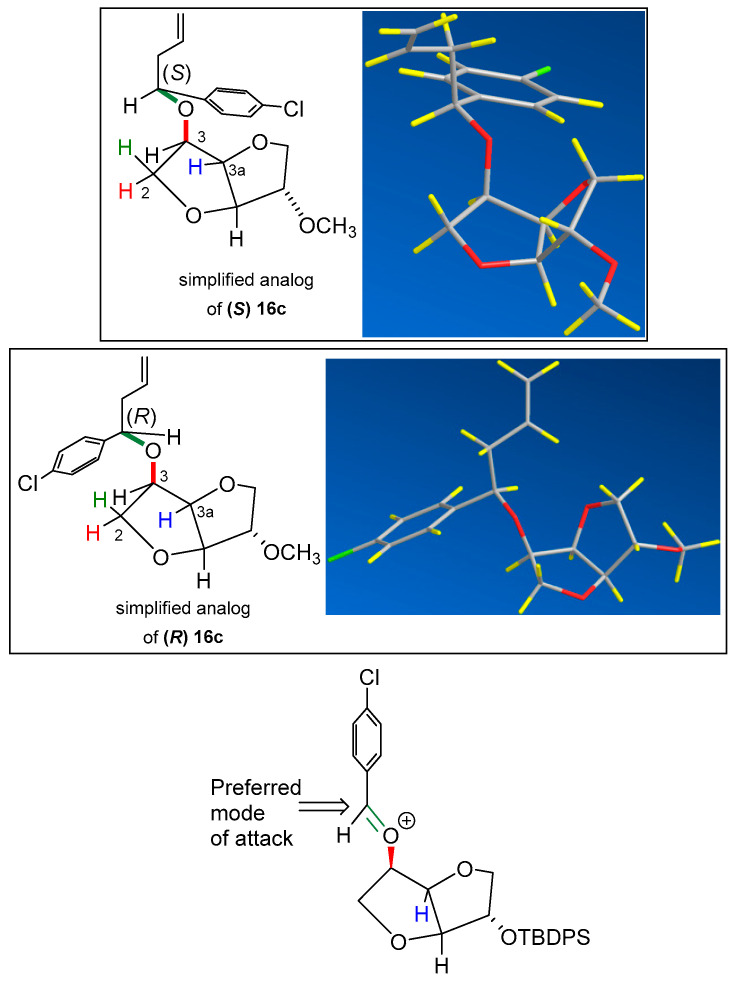
Presumed preferred conformations for (*S*) and (*R*) isomers for a simplified analog of **1** (the TBDPS group was substituted with a methyl for the sake of clarity) and rationalization of diastereoselectivity.

As far as it concerns the relative configuration of the major diastereomer, we noticed, for products **16a**–**16i**, derived from aromatic aldehydes, a significant difference in the δ values (^1^H NMR) for *H*-3a and *H*-2 (especially one of them). In the major diastereomer, *H*-3a always resonates upfield, by 0.22–0.36 ppm, whereas the *H*-2 protons always resonate downfield, with a difference of 0.28–0.37 for one of them or 0.07–0.10 ppm for the other one. These differences completely disappear on passing to the products **16k** and **16l** derived from aliphatic aldehydes. Therefore, these trends are likely due to the anisotropic effect of the aromatic ring. A conformational study, which is detailed in the Supplementary Materials, allowed us to tentatively propose the preferred conformations shown in Scheme 5.

Thanks to the high rigidity of the bicyclic system, different conformations may arise only from rotation around the bonds colored in red and green. Rotation around the bond colored in red with no doubt gives, as the best conformations, those placing the bulky substituent away from the bicyclic isosorbide system (outside). As regards the three possible staggered conformations resulting from rotation around the bond colored in green, the one placing the allyl group *anti* to the O–C3 bond resulted in the most stable one from our minimization, which is also in good agreement with nOe experiments. If this is true, in the (*S*) isomer the aromatic ring will shield *H*-3a, whereas in the (*R*) isomer, it will shield *H*-2.

Therefore, we suppose that the main diastereomer has an (*S*) configuration. However, we must stress the fact that we cannot be 100% sure of this assignment, although it appears in line with a logical rationalization of the observed outcomes. The key intermediate is an oxocarbenium ion, depicted in Scheme 5, which likely would put the aryl group as far away as possible from the bicyclic system. Then the allyl nucleophile will preferably attack from the left (*Re* face), since the V-shaped bicyclic system is expected to encumber the right one (*Si* face).

In conclusion, application of the multicomponent Hosomi–Sakurai reaction on monoprotected isosorbide derivative **6** proved to be a good method to join to the glycomimetic, bio-based bicyclic system of isosorbide to aldehyde-derived aromatic or aliphatic fragments. This method can therefore find application in fragment-based drug discovery, also thanks to the expected high metabolic stability of the ether bond that connects the two fragments.

**Scheme 6:**
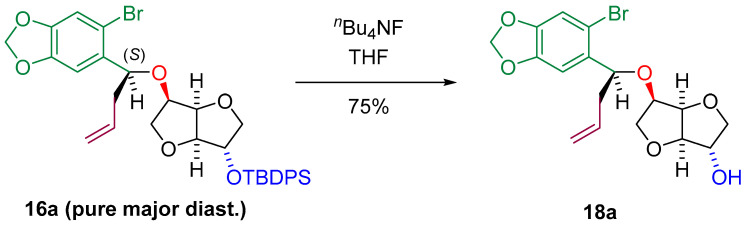
Deprotection of **16a** (pure major diastereomer) to give alcohol **18a**.

Obviously the *exo* OH can be easily deprotected, as shown in Scheme 6 for compound **16a**. The free hydroxy group at carbon 6 can thus be further manipulated (e.g., by acylation or ether formation), allowing the addition of a second fragment. Finally, the allyl group represents an additional handle that can be functionalized and/or used to attach further fragments.

## 3. Experimental Section

NMR spectra were taken at the indicated temperature in CDCl_3_ at 400 MHz (^1^H) and 100 MHz (^13^C), using, as an internal standard, TMS (^1^H NMR: 0.000 ppm) or the central peak of CDCl_3_ (^13^C: 77.02 ppm). Chemical shifts are reported in ppm (δ scale). Peak assignments were made with the aid of COSY and HSQC experiments. In the ABX system, proton A is the one upfield. GC-MS analyses were carried out using a Shimadzu (Kyoto, Japan) GC−MS-QP2010 SE spectrometer with an HI-5 MS column (0.25 mm, 0.25 mm, 30 m) and with these conditions: METHOD A: Flow: 1.03 mL/min, split ratio: 10.0. Initial temp.: 70 °C, hold time: 4 min, rate: 25 °C/min. METHOD B: Flow: 0.96 mL/min, split ratio: 10.0. Initial temp.: 150 °C, hold time: 3 min, rate: 25 °C/min. HRMS: samples were analyzed with a Waters (Milford, MA, USA) Synapt G2 QToF mass spectrometer. MS signals were acquired from 50 to 1200 *m*/*z* in ESI positive ionization mode. TLC analyses were carried out on silica gel plates and viewed at UV (254 nm) and/or developed with Hanessian stain (dipping into a solution of (NH_4_)_4_MoO_4_·4 H_2_O (21 g) and Ce(SO_4_)_2_·4 H_2_O (1 g) in H_2_SO_4_ (31 mL) and H_2_O (469 mL) and warming). R*_f_* were measured after an elution of 7–9 cm. Column chromatographies were done with the “flash” methodology using 220–400 mesh silica. Petroleum ether (40–60 °C) is abbreviated as PE. In extractive work-up, aqueous solutions were always reextracted three times with the appropriate organic solvent. Organic extracts were always dried over Na_2_SO_4_ and filtered before evaporation of the solvent under reduced pressure. All reactions using dry solvents were carried out under a nitrogen or argon atmosphere.

*For the sake of clarity, for all isosorbide derivatives, we have maintained the same numbering of the parent compound shown in Scheme 1.*

**(3*R*,3a*R*,6*S*,6a*R*)-6-hydroxyhexahydrofuro [3,2-*b*]furan-3-yl acetate 5**. A solution of isosorbide **1** (3.00 g, 20.5 mmol) in acetone (22.7 mL) is treated with vinyl acetate (5.7 mL, 61.5 mmol) and with Amano PS lipase SD (563 mg). The suspension is stirred at rt for 28 h. The enzyme is filtered off, washing it with EtOAc. Evaporation to dryness affords a colorless oil, pure enough (by ^1^H NMR) to be used for the next step (3.82 g, 99%). The peaks of the diastereomer **8** cannot be detected at ^1^H NMR. An analytical sample can be obtained by chromatography using PE/EtOAc 8:2 as the eluent.

This compound is already known [26,28].

R*_f_*: 0.60 (EtOAc). **[α]_D_** +106.8 (c 2, CHCl_3_) [28]. **GC-MS**: R*_t_* 9.67 min. *m*/*z*: 146 (M^+^−42, 0.8), 145 (M^+^−43, 0.9), 128 (9.1), 98 (4.9), 86 (4.4), 85 (15.7), 70 (4.3), 69 (27.5), 68 (18.6), 58 (5.4), 57 (10.9), 55 (5.9), 45 (6.5), 44 (11.8), 43 (100.0), 42 (6.0), 41 (10.5), 39 (7.0). **^1^H NMR** (400 MHz, CDCl_3_): δ 5.15 (q, 1H, *J* = 5.5 Hz, *H*-3), 4.85 (t, 1H, *J* = 4.9 Hz, *H*-3a), 4.41 (d, 1H, *J* = 4.5, *H*-6a), 4.35 (s, 1H, *H*-6), 3.97–3.86 (m, 3H, *H*-5 + one *H*-2), 3.77 (dd, 1H, *J*= 9.8, 5.4, one *H*-2), 2.13 (s, 3H, C*H*_3_CO). **^13^C NMR** (101 MHz, CDCl_3_) δ 170.6 (*C*=O), 88.0 (*C*-6a), 80.2 (*C*-3a), 75.8 (*C*-6), 75.4 (*C*-5), 74.0 (*C*-3), 70.0 (*C*-2), 20.6 (*C*H_3_CO).

**(3*R*,3a*R*,6*S*,6a*S*)-6-((tert-Butyldiphenylsilyl)oxy)hexahydrofuro [3,2-*b*]furan-3-ol 6**. A solution of crude monoacetate **5** (3.82 g, 20.3 mmol) in dry DMF (21.0 mL) is treated with imidazole (2.07 g, 30.5 mmol) and *tert*-butyldiphenylsilyl chloride (6.07 mL, 23.3 mmol). The solution is stirred at rt for 20 h. Then it is diluted with water (60 mL) and extracted thrice with PE/Et_2_O 1:1. The united organic phases are washed with water and then with brine. After drying and evaporation to dryness, a colorless oil is obtained, which contains pure product **6** plus some *tert*-butyldiphenylsiloxane (9.27 g). This oil is taken up in methanol (150 mL), cooled to 0 °C and treated with a 1 M solution of KOH in methanol (41 mL, 41 mmol). After 10 min, the cooling bath is removed, and the solution is stirred until the reaction is complete by TLC (2h and 30 min). Acetic acid (2.35 mL, 41 mmol) is added. The solvent is evaporated, and the crude mixture (an oil containing some solid AcOK) is directly chromatographed, using 80 g of silica and PE/EtOAc from 8:2 to 7:3, to give pure **6** as an oil (7.10 g, 91%).

This compound is already known [29].

R*_f_*: 0.44 (PE/EtOAc 70:30). **[α]_D_** +22.2 (c 1, CHCl_3_). **^1^H NMR** (400 MHz, CDCl_3_): δ 7.68–7.60 (m, 4H, meta aromatic C*H*), 7.48–7.36 (m, 6 H, ortho and para aromatic C*H*), 4.70 (t, 1H, *J* = 4.8 Hz, *H*-3a), 4.38 (d, 1H, *J* = 4.0, *H*-6a), 4.35 (d, 1H, *J* = 3.2, *H*-6), 4.27 (dq, 1H, *J* = 8.0, 6.0, *H*-3), 3.88 (d, 1H, *J* = 10.0, *H*-5), 3.80 (dd, 1H, *J* = 9.4, 6.2, *H*-2), 3.66 (dd, 1H, *J* = 10.0, 3.2, *H*-5), 3.41 (dd, 1H, *J* = 9.2, 6.0, *H*-2), 2.62 (d, 1H, *J* = 8.4, O*H*), 1.07 (s, 9H, C(C*H*_3_)_3_). **^13^C NMR** (101 MHz, CDCl_3_) δ 135.6 (×2) (*C*H ortho to Si), 133.3, 133.1 (quat.), 130.00, 129.96 (*C*H para to Si), 127.9, 127.8 (*C*H meta to Si), 88.2 (*C*-6a), 81.7 (*C*-3a), 78.1 (*C*-6), 75.8 (*C*-5), 73.4 (*C*-2), 72.4 (*C*-3), 26.8 (C(*C*H_3_)_3_), 19.1 (*C*(CH_3_)_3_).

**(3*R*,3a*R*,6*S*,6a*R*)-hexahydrofuro[3,2-*b*]furan-3,6-diyl diacetate 7**. A suspension of isosorbide **1** (1.00 g, 6.84 mmol) in dry CH_2_Cl_2_ (13.7 mL) is treated with 4-(*N*,*N*-dimethylamino)pyridine (84 mg, 0.684 mmol) and triethylamine (3.8 mL, 27.4 mmol). The suspension becomes a solution. Finally, after cooling to 0 °C, acetic anhydride (1.90 mL, 20.5 mmol) is added dropwise. After 5 min, the cooling bath is removed, and the solution is stirred at rt for 1 h and 45 min. The reaction is quenched with saturated aqueous NH_4_Cl. Extraction with CH_2_Cl_2_, washing of the organic phases with brine, drying and evaporation give a crude product (yellow cloudy oil) nearly pure by ^1^H NMR. Anyway, it is chromatographed with PE/EtOAc 1:1 to give pure **7** as an oil (1.54 g, 98%).

This compound is already known [27,30].

R*_f_*: 0.47 (PE/EtOAc 50:50). **^1^H NMR** (400 MHz, CDCl_3_): δ 5.20–5.18 (m, 1H, *H*-6), 5.15 (q, 1H, *J* = 5.6, *H*-3), 4.83 (t, 1H, *J* = 5.0, *H*-3a), 4.49 (d, 1H, *J* = 4.6, *H*-6a), 4.03–3.96 (m, 2H, *H*-5), 3.95 (dd, 1H, *J* = 9.8, 6.0, *H*-2), 3.80 (dd, 1H, *J* = 9.7, 5.5, *H*-2), 2.13, 2.08 (2 s, 2×3H, C*H*_3_). **^13^C NMR** (101 MHz, CDCl_3_) δ 170.4, 170.1 (*C*=O), 85.9 (*C*-6a), 80.7 (*C*-3a), 78.1 (*C*-6), 73.9 (*C*-3), 73.5 (*C*-5), 70.2 (*C*-2), 20.9, 20.7 (*C*H_3_).

**(3*R*,3a*R*,6*S*,6a*R*)-3-hydroxyhexahydrofuro[3,2-*b*]furan-6-yl acetate 8**. A solution of diacetate **7** (3.088 g, 13.4 mmol) in THF (6.7 mL) is treated with 79.6 mL (39.8 mmol) of a 0.5 M pH 7 phosphate buffer solution (freshly prepared by mixing 63.4 mL of a 0.5 M solution of NaH_2_PO_4_ in distilled water and 98.1 mL of a 0.5 M solution of Na_2_HPO_4_ in distilled water). This suspension is treated with Amano PS lipase SD (618 mg) and stirred at rt overnight. After 22 h, the reaction is complete by TLC. Solid NaCl is added up to saturation, and the aqueous phase is extracted several times with EtOAc (emulsions were observed anyway). Drying and evaporation furnish the crude product as a solid (1.892), which seems nearly pure at ^1^H NMR. Anyway, it is chromatographed (EtOAc) to give pure **8** as a white solid (1.823 mg, 72%). A slightly lower yield is obtained when extractions are carried out with CH_2_Cl_2_ as described in a previous work [27]. This reaction is not thoroughly optimized. We think that it should be better monitored in order to avoid full hydrolysis to isosorbide, which is then lost in the water phase upon extraction. D.r. was determined by ^1^H NMR on the crude product and was = 98.5:1.5. This product is already known [27,32,33,34].

R*_f_*: 0.51 (EtOAc). **M.p.**:74–75 °C. **^1^H NMR** (400 MHz, CDCl_3_): δ 5.22 (d, 1H, *J* = 3.1, *H*-6), 4.64 (t, 1H, *J* = 4.9, *H*-3a), 4.48 (d, 1H, *J* = 4.3, *H*-6a), 4.31 (dq, 1H, *J* = 7.2, 5.6, *H*-3), 4.04, 4.01 (AB part of an ABX syst., 2H, *J*_AB_ = 10.5, *J*_AX_ = 4.0, *J*_BX_ = 0, *H*-5), 3.90 (dd, 1H, *J* = 9.5, 5.9, *H*-2), 3.58 (dd, 1H, *J* = 9.5, 5.9, *H*-2), 2.60 (dd, 1H, *J* = 7.3, 2.0, O*H*), 2.09 (s, 3H, C*H*_3_). **^13^C NMR** (101 MHz, CDCl_3_) δ 170.0 (*C*=O), 85.6 (*C*-6a), 81.9 (*C*-3a), 78.4 (*C*-6), 73.6, 73.5 (*C*-5, *C*-2), 72.3 (*C*-3), 20.9 (*C*H_3_).

**(3*R*,3a*S*,6*S*,6a*R*)-3-((tert-Butyldiphenylsilyl)oxy)hexahydrofuro[3,2-*b*]furan-6-ol 9**. It is prepared in 81% overall yield from monoacetate **8** following the same procedure described for the synthesis of **6**.

Colorless oil. R*_f_*: 0.41 (PE/EtOAc 70:30). **[α]_D_**: + 56.7 (c 1.6, CHCl_3_). **GC-MS** (method A): R*_t_*: 14.84 min. *m*/*z*: 327 (M^+^-57, 62.1), 281 (5.6), 253 (11.3), 241 (6.7), 225 (6.7), 223 (13.5), 207 (17.2), 205 (8.2), 199 (51.4), 197 (10.7), 191 (7.2), 183 (15.7), 182 (5.4), 181 (24.4), 177 (7.7), 171 (6.1), 165 (6.1), 164 (5.6), 163 (33.7), 147 (7.8), 145 (10.2), 139 (19.3), 135 (15.3), 129 (7.4), 123 (7.7), 121 (9.8), 115 (11.9), 105 (19.4), 103 (10.9), 91 (14.7), 85 (5.3), 79 (7.6), 78 (26.8), 77 (26.9), 73 (10.4), 69 (100), 68 (5.1), 57 (22.2), 56 (8.6), 55 (7.1), 52 (5.7), 51 (9), 50 (5.8), 45 (17.6), 44 (58.1), 43 (27.4), 42 (8.3), 41 (44), 40 (6.5), 39 (27.2), 38 (5). **^1^H NMR** (400 MHz, CDCl_3_): δ 7.77–7.72 (m, 2 H), 7.69–7.63 (m, 2H), 7.47–7.35 (m, 6H), 4.40 (t, 1H, *J* = 4.6 Hz, *H*-3a), 4.32–4.26 (m, 2H, *H*-6, *H*-6a), 4.21 (q, 1H, *J* = 6.6 Hz, *H*-3), 4.02 (dd, 1H, *J* = 10.2, 3.4, *H*-5), 3.95 (d, 1H, *J* = 10.2, *H*-5), 3.61 (dd, 1H, *J* = 8.6, 6.3, *H*-2), 3.53 (t, 1H, *J* = 7.8, *H*-2), 1.90–1.75 (m, 1H, O*H*), 1.08 (s, 9H, C(C*H*_3_)_3_). **^13^C NMR** (101 MHz, CDCl_3_) δ 135.8, 135.6 (*C*H ortho to Si), 133.6, 133.3 (quat.), 129.9 (×2) (*C*H para to Si), 127.8 (×2) (*C*H meta to Si), 87.9 (*C*-6a), 81.3 (*C*-3a), 76.9 (*C*-6), 75.7 (*C*-5), 74.2 (*C*-3), 71.9 (*C*-2), 26.8 (C(*C*H_3_)_3_), 19.2 (*C*(CH_3_)_3_). **HRMS** (ESI+): calcd. for C_22_H_29_O_4_Si^+^ [M + H]+ 385.18296, found 385.18311.

**General procedure for the preparation of TMS ethers 10–13**. To a solution of alcohol **5**–**6** or **8**–**9** (3.00 mmol) in dry CH_2_Cl_2_ (10 mL), iodine (60.0 µmol, 2 mol%) and hexamethyldisilazane (2.40 mmol) are added at rt. The initially colorless solution turns brown, and the color fades over 10 min. Then, solid Na_2_S_2_O_3_·5H_2_O (968 mg, 3.90 mmol) is added, and the reaction mixture turns colorless. The mixture is stirred for 30 min and then filtered quickly through a silica plug (1 cm), washing with CH_2_Cl_2_ (30 mL). The solvent is removed, and the silyl ether is used as such without further purification. It is recommended not to strip the solvent at high vacuum (10^−2^ mbar), because these TMS ethers tend to be slightly volatile at this pressure. In the case of TMS ether **11**, we checked its stability upon storage, and we found out that it was quite stable for months in the freezer.

**(3*R*,3a*R*,6*S*,6a*R*)-6-[(1-(*R*,*S*)-(5-bromobenzo[*d*][1,3]dioxole-5-yl)but-3-en-1-yl)oxy]-hexahydrofuro[3,2-*b*]furan-3-yl acetate 14a**. A solution of TMS ether **10** (164 mg, 670 µmol) in dry CH_2_Cl_2_ (2.9 mL) is added with 5-bromopiperonal (3-bromobenzo[*d*][1,3]dioxole-5-carboxyaldehyde) (133 mg, 581 µmol, 0.2 M). The solution is cooled to −78 °C and treated with allyltrimethylsilane (139 µL, 0.87 mmol) and then with a freshly prepared 0.3 M solution of TMS-OTf (trimethylsilyl trifluoromethanesulfonate) in dry CH_2_Cl_2_ (580 µL, 174 µmol). Note: if these two additions take more than 5 min, some precipitation of 5-bromopiperonal may be observed, but this does not invalidate the positive outcome of the reaction. The solution is stirred at −78 °C for 2 h and then quenched by the addition of a saturated aqueous solution of NaHCO_3_. After warming, the mixture is extracted with EtOAc, dried, and evaporated to dryness. The crude product is chromatographed (PE/EtOAc 6:4) to give product **14a** as a mixture of two inseparable diastereomers X (major) and Y (minor) (128 mg, 50%). The diastereomeric ratio was determined by ^1^H NMR on the crude product by integrating the doublets at 4.53 (X) and 4.37 (Y) and resulted = 57:43.

Colorless oil. R*_f_*: 0.26 (CH_2_Cl_2_/PE 5:1). **[α]_D_**: + 45.7 (c 1, CHCl_3_). **GC-MS** (method B): R*_t_*: 11.02 (Y), 11.09 (X). *m*/*z* (Y): 401 (M^+^-41) (11.1), 399 (M^+^-41) (11.3), 231 (3.3), 229 (5.1), 174 (10.1), 173 (4.8), 171 (22.9), 144 (7.8), 116 (13.1), 115 (19.6), 111 (43.1), 85 (5.4), 83 (27.3), 69 (54.3), 55 (9.0), 44 (6.3), 43 (100), 41 (12.0), 39 (5.1). *m*/*z* (X): 401 (M^+^-41) (9.8), 399 (9.9), 229 (5.0), 174 (10.0), 173 (4.8), 171 (23.3), 144 (8.1), 116 (13.6), 115 (21.4), 111 (43.3), 85 (5.5), 83 (27.5), 69 (53.9), 55 (9.1), 44 (5.1), 43 (100,0), 41 (11.9), 39 (5.0). **^1^H NMR** (400 MHz, CDCl_3_): δ 6.96, 6.90 (X) (2 s, 2H, *H*-3′, *H*-6′), 6.95, 6.94 (Y) (2 s, 2H, *H*-3′, *H*-6′), 6.02–5.93 (X + Y) (m, 2H, OC*H*_2_O), 5.89–5.74 (X + Y) (m, 1H, C*H*=CH_2_), 5.18–5.00 (m, 3H, CH=C*H*_2_ + *H*-3), 4.82–4.75 (m, 1.57 H, *H*-3a (X + Y) + C*H*-CH_2_CH= (X)), 4.72 (Y) (dd, 0.43 H, *J* = 7.0, 5.8, C*H*-CH_2_CH = (Y), 4.53 (X) (d, 0.57H, *J* = 4.5, *H*-6a), 4.37 (Y) (d, 0.43H, *J* = 4.3, *H*-6a), 4.10 (Y) (d, 0.43H, *J* = 10.1, *H*-5), 4.00–3.73 (m, 2.57H, *H*-2, *H*-5), 3.73–3.63 (X + Y) (m, 1H, *H*-2), 2.43–2.28 (X + Y) (m, 2H, C*H*_2_CH=), 2.12 (Y), 2.10 (X) (2s, 3H, C*H*_3_C=O). **^13^C NMR** (101 MHz, CDCl_3_) δ 170.41, 170.39 (*C*=O, X + Y), 147.91, 147.84, 147.83, 147.77 (*C*-2a′, *C*-6a′, X + Y), 134.1 (C*H*=CH_2_), 133.9 (X), 133.8 (Y) (*C*-4′), 117.7 (X), 117.5 (Y) (CH=*C*H_2_), 113.1 (X), 113.0 (Y) (*C*-5′), 112.3 (X), 112.2 (Y) (*C*-3′), 107.4 (Y), 107.3 (X) (*C*-6′), 101.8 (Y), 101.7 (X) (O*C*H_2_O), 86.5 (Y) 85.8 (X) (*C*-6a), 81.9 (X) 81.6 (Y) (*C*-3a), 80.5 (X) 80.4 (Y) (*C*H-CH_2_CH=), 79.3 (X), 78.5 (Y) (*C*-6), 74.2 (Y), 74.04 (X) (*C*-3), 73.97 (X), 72.7 (Y) (*C*-5), 69.63, 69.61 (X + Y) (*C*-2), 41.1 (X + Y) (*C*H_2_-CH=), 20.7 (X + Y) (*C*H_3_C=O). **HRMS** (ESI+): calcd. for C_19_H_22_BrO_7_^+^ [M + H]+ 441.0543, found 441.0541.

**(3*R*,3a*S*,6*S*,6a*R*)-6-[(1-(*R*,*S*)-(5-bromobenzo[*d*][1,3]dioxole-5-yl)but-3-en-1-yl)oxy]-3-((*tert*-butyldiphenylsilyl)oxy)hexahydrofuro[3,2-*b*]furan 15a**. It is prepared, starting from 137.9 mg of TMS ether **13** (302 µmol), following the same procedure described above for the synthesis of **14a**. The reaction time was, however, 3.5 h. Analysis of the crude mixture at ^1^H NMR indicated a diastereomeric ratio = 53: 47. Chromatography (CH_2_Cl_2_/PE 2:1) gives a diastereomeric mixture of **15a** (91.5 mg, 53%). Through a second careful chromatography, it is possible to obtain pure or enriched samples of the two diastereomers. R*_f_*: 0.46 (major) and 0.43 (minor) (CH_2_Cl_2_/PE 2:1).

**Major diastereomer (faster running)**:

**^1^H NMR** (400 MHz, CDCl_3_): δ 7.77–7.70 (m, 2 H, ortho C*H* of TBDPS), 7.68–7.62 (m, 2H, ortho C*H* of TBDPS), 7.47–7.35 (m, 6H, para and meta C*H* of TBDPS), 6.93, 6.91 (2 s, 2H, *H*-3′, *H*-6′), 6.00–5.90 (m, 2H, *H*-1′), 5.87–5.71 (m, 1H, C*H*=CH_2_), 5.10–4.96 (m, 2H, CH=C*H*_2_), 4.74 (t, 1H, *J* = 6.1, C*H*-CH_2_C=), 4.36 (t, 1H, *J* = 4.3, *H*-6), 4.23 (d, 1H, *J* = 4.1, *H*-6a), 4.20–4.14 (m, 1H, *H*-3), 4.12 (d, 1H, *J* = 10.3, *H*-5), 3.98–3.82 (m, 2H, *H*-3a, *H*-5), 3.56–3.40 (m, 2H, *H*-2), 2.46–2.29 (m, 2H, C*H*_2_CH=C), 1.07 (s, 9H, C(C*H*_3_)_3_). **^13^C NMR** (101 MHz, CDCl_3_) δ 147.9, 147.8 (*C*-2a′, *C*-6a′), 135.8, 135.6 (*C*H ortho to Si), 134.0 (*C*H=CH_2_ + *C*-4′), 133.6, 133.3 (quat. TBDPS), 129.8 (*C*H para to Si), 127.7 (*C*H meta to Si), 117.5 (CH=*C*H_2_), 113.1 (*C*-5′), 112.2 (*C*-3′), 107.6 (*C*-6′), 101.8 (*C*-1′), 86.4 (*C*-6a), 82.4 (*C*-3a), 81.6 (*C*-6), 78.5 (*C*H-CH_2_CH=), 74.3 (*C*-3), 72.9 (*C*-5), 71.4 (*C*-2), 41.2 (*C*H_2_-CH=), 26.8 (C(*C*H_3_)_3_), 19.2 (*C*(CH_3_)_3_). **HRMS** (ESI+): calcd. for C_33_H_38_BrO_6_Si^+^ [M + H]+ 637.16155, found 637.1621.

**Minor diastereomer (slower running) (only enriched; ratio: 3:1)**:

**[α]_D_**: + 14.4 (c 1, CHCl_3_). **^1^H NMR** (400 MHz, CDCl_3_): δ 7.77–7.70 (m, 2 H, ortho C*H* of TBDPS), 7.68–7.62 (m, 2H, ortho C*H* of TBDPS), 7.47–7.35 (m, 6H, para and meta C*H* of TBDPS), 6.95, 6.91 (2 s, 2H, *H*-3′, *H*-6′), 6.02–5.92 (m, 2H, *H*-1′), 5.87–5.69 (m, 1H, C*H*=CH_2_), 5.11–4.95 (m, 2H, CH=C*H*_2_), 4.75 (mc) (m, 1H, C*H*-CH_2_C=), 4.42–4.32 (m, 2H, *H*-6, *H*-6a), 4.22–4.15 (m, 1H, *H*-3), 3.98–3.83 (m, 2H, *H*-5), 3.80 (s, 1H, *H*-3a), 3.64–3.41 (m, 2H, *H*-2), 2.37–2.29 (m, 2H, C*H*_2_CH=C), 1.06 (s, 9H, C(C*H*_3_)_3_). **^13^C NMR** (101 MHz, CDCl_3_) δ 147.9, 147.8 (*C*-2a′, *C*-6a′), 135.8, 135.6 (*C*H ortho to Si), 134.3 (C-4′), 134.2 (*C*H=CH_2_), 133.6, 133.3 (quat. TBDPS), 129.8 (*C*H para to Si), 127.7 (*C*H meta to Si), 117.5 (CH=*C*H_2_), 113.2 (*C*-5′), 112.4 (*C*-3′), 107.4 (*C*-6′), 101.8 (*C*-1′), 85.6 (*C*-6a), 82.4 (*C*-3a), 81.7 (*C*-6), 79.2 (*C*H-CH_2_CH=), 74.2 (*C*-3), 74.1 (*C*-5), 71.6 (*C*-2), 41.2 (*C*H_2_-CH=), 26.8 (C(*C*H_3_)_3_), 19.2 (*C*(CH_3_)_3_). **HRMS** (ESI+): calcd. for C_33_H_38_BrO_6_Si^+^ [M + H]+ 637.16155, found 637.1629.

**(3*R*,3a*R*,6*S*,6a*S*)-3-[(1-(*R*,*S*)-(5-bromobenzo[*d*][1,3]dioxole-5-yl)but-3-en-1-yl)oxy]-6-((*tert*-butyldiphenylsilyl)oxy)hexahydrofuro[3,2-*b*]furan 16a**. A solution of TMS ether **11** (312.6 mg, 684 µmol) in dry CH_2_Cl_2_ (2.45 mL) is added with 5-bromopiperonal (3-bromobenzo[*d*][1,3]dioxole-5-carboxyaldehyde) (112.2 mg, 490 µmol, 0.2 M). The solution is cooled to −78 °C and treated with allyltrimethylsilane (117 µL, 735 µmol) and then with a freshly prepared 0.3 M solution of TMS-OTf (trimethylsilyl trifluoromethanesulfonate) in dry CH_2_Cl_2_ (490 µL, 147 µmol). Note: if these two additions take more than 5 min, some precipitation of 5-bromopiperonal may be observed, but this does not invalidate the positive outcome of the reaction. The solution is stirred at −78 °C for 2 h and then quenched by the addition of a saturated aqueous solution of NaHCO_3_. After warming, the mixture is extracted with EtOAc, dried, and evaporated to dryness. The crude product is chromatographed (PE/EtOAc 92:8) to give the diastereomerically pure major diastereomer of **16a** (faster running) (202 mg) plus some mixed fractions containing both (76 mg). Overall yield: 89%. The diastereomeric ratio was determined by ^1^H NMR of the crude product and resulted = 86:14. R*_f_*: 0.44 (major) and 0.38 (minor) (PE/EtOAc 9:1). Only the major diastereomer was fully characterized.

**Major diastereomer (faster running).**

**[α]_D_**: −7.4 (c 0.78, CHCl_3_) **^1^H NMR** (400 MHz, CDCl_3_): δ 7.65–7.58 (m, 4H, C*H* meta of TDBDPS), 7.46–7.33 (m, 6H, C*H* ortho and para of TBDPS), 7.11 (s, 1H, *H*-6′), 6.96 (s, 1H, *H*-3′), 6.00 (d, 1H, *J* = 1.2, O-C*H*H-O), 5.99 (d, 1H, *J* = 1.2, O-C*H*H-O), 5.78 (ddt, 1H, *J* = 16.8, 10.4, 7.0, C*H*=CH_2_), 5.08–4.93 (m, 2H, CH=C*H*_2_), 4.68 (dd, 1H, *J* = 7.7, 5.4, C*H*-CH_2_C=), 4.45 (t, 1H, *J* = 4.0, *H*-3a), 4.31 (d, 1H, *J* = 3.8, *H*-6a), 4.26 (mc) (m, 1H, *H*-6), 3.92–3.79 (m, 3H, *H*-3 + 1 *H*-2 + 1 *H*-5), 3.76 (dd, 1H, *J* = 9.8, 3.6, *H*-5), 3.49 (t, 1H, *J*= 8.0, *H*-2), 2.48 (dt, 1H, *J* = 14.2, 7.1, C*H*HC=), 2.36 (dt, 1H, *J* = 14.1, 6.4, C*H*HC=), 1.04 (s, 9H, C(C*H*3)3). **^13^C NMR** (101 MHz, CDCl_3_) δ 147.95, 147.87 (*C*-2a′, *C*-6a′), 135.6 (*C*H ortho to Si), 134.1, 134.0 (*C*-4′, *C*H=CH_2_), 133.5, 133.2 (quat. of TBDPS), 129.88, 129.85 (*C*H para to Si), 127.8, 127.7 (*C*H meta to Si), 117.4 (CH=*C*H_2_), 113.3 (*C*-5′), 112.2 (*C*-3′), 107.8 (*C*-6′), 101.8 (O-*C*H_2_O), 88.1 (*C*-6a), 80.9 (*C*-3a), 80.6 (*C*H-CH_2_C=), 78.4 (*C*-3), 78.2 (*C*-6), 76.1 (*C*-5), 69.0 (*C*-2), 41.4 (*C*H_2_-CH=), 26.8 (C(*C*H3)3), 19.1 (*C*(CH3)3). **HRMS** (ESI+): calcd. for C_33_H_38_BrO_6_Si^+^ [M + H]+ 637.16155, found 637.1607.

**Minor diastereomer (slower running).**

Only NMR data, taken from a mixed fraction, are reported. **^1^H NMR** (400 MHz, CDCl_3_): δ 7.66–7.59 (m, 4H, C*H* meta of TDBDPS), 7.46–7.33 (m, 6H, C*H* ortho and para of TBDPS), 6.96 (s, 1H, *H*-6′), 6.86 (s, 1H, *H*-3′), 5.99–5.95 (m, 2H, O C*H*2-O), 5.82 (ddt, 1H, *J* = 16.8, 10.4, 7.0, C*H*=CH2), 5.08–4.96 (m, 3H, CH=C*H*2, C*H*-CH_2_C=), 4.73 (t, 1H, *J* = 4.1, *H*-3a), 4.32 (d, 1H, *J* = 4.0, *H*-6a), 4.26 (mc) (m, 1H, *H*-6), 3.89 (d, 1H, *J* = 9.7, *H*-5), 3.84–3.74 (m, 2H, *H*-3 + 1 *H*-2), 3.58 (dd, 1H, *J* = 8.5, 7.0, *H*-5), 3.39 (t, 1H, *J* = 8.4, *H*-2), 2.57 (dtt, 1H, *J* = 14.4, 7.1, C*H*H-C=), 2.40 (dt, 1H, *J* = 14.1, 6.4, C*H*H-C=), 1.06 (s, 9H, C(C*H*3)3). **^13^C NMR** (101 MHz, CDCl_3_) δ 147.9 (*C*-2a′, *C*-6a′), 135.6 (*C*H ortho to Si), 134.2 (*C*-4′), 133.8 (*C*H=CH_2_), 133.4, 133.3 (quat. of TBDPS), 129.9 (*C*H para to Si), 127.8 (*C*H meta to Si), 117.3 (CH=*C*H_2_), 113.7 (*C*-5′), 112.3 (*C*-3′), 107.4 (*C*-6′), 101.8 (O-*C*H_2_-O), 88.5 (*C*-6a), 80.0 (*C*-3a), 79.4 (*C*H-CH_2_C=), 78.2 (*C*-3, *C*-6), 76.2 (*C*-5), 69.6 (*C*-2), 41.4 (*C*H_2_C=), 26.8 (C(*C*H3)3), 19.1 (*C*(CH3)3).

**(3*R*,3a*R*,6*S*,6a*R*)-3-[(1-(*R*,*S*)-(5-bromobenzo[*d*][1,3]dioxole-5-yl)but-3-en-1-yl)oxy]-hexahydrofuro[3,2-*b*]furan-6-yl acetate 17a**. It is prepared, starting from 103 mg of bromopiperonal (450 µmol) and TMS ether **12** (164 mg, 630 µmol), following the same procedure described above for the synthesis of **16a**. The reaction time is 2 h. Analysis of the crude mixture at ^1^H NMR indicated a diastereomeric ratio = 89:11. Chromatography (PE/EtOAc 3:6) gives the diastereomeric mixture of **17a** (140.1 mg, 71%). A small analytical sample of the major diastereomer (slower running) and minor diastereomer (faster running) can be obtained by a second chromatography. R*_f_*: 0.51 (major) and 0.59 (minor) (PE/EtOAc 3:6). Only the major isomer was fully characterized.

**Major diastereomer (slower running).**

**[α]_D_**: + 6.1 (c= 0.4, CHCl_3_)**^1^H NMR** (400 MHz, CDCl_3_): δ 7.11 (s, 1H, *H*-6′), 6.96 (s, 1H, *H*-3′), 6.03–6.56 (m, 2H, O-C*H*_2_-O), 5.81 (ddt, 1H, *J* = 17.2, 10.2, 7.0, C*H*=CH_2_), 5.14 (d, 1H, *J* = 3.8, *C*-6), 5.11–4.99 (m, 2H, CH=C*H*_2_), 4.73 (dd, 1H, *J* = 7.8, 5.1, C*H*-CH_2_C=), 4.45–4.39 (m, 2H, *H*-3a, *H*-6a), 4.12 (dd, 1H, *J* = 10.7, 4.0, *H*-5), 3.95 (d, 1H, *J* = 10.0, *H*-5), 3.93–3.83 (m, 2H, *H*-2, *H*-3), 3.68 (dd, 1H, *J* = 8.0, 7.2, *H*-2), 2.54–2.33 (m, 2H, C*H*_2_C=), 2.05 (s, 3H, C*H*_3_). **^13^C NMR** (101 MHz, CDCl_3_) δ 170.1 (*C*=O), 147.93, 147.87 (*C*-2a′, *C*-6a′), 134.05, 134.02 (*C*-4′, *C*H=CH_2_), 117.5 (CH=*C*H_2_), 113.1 (*C*-5′), 112.2 (*C*-3′), 107.7 (*C*-6′), 101.8 (O-*C*H_2_O), 85.6 (*C*-6a), 81.1 (*C*-3a), 80.7 (*C*H-CH_2_C=), 78.6 (*C*-6), 78.3 (*C*-3), 73.8 (*C*-5), 69.7 (*C*-2), 41.3 (*C*H_2_-CH=), 21.0 (*C*H_3_). **HRMS** (ESI+): calcd. for C_19_H_22_BrO_7_^+^ [M + H]+ 441.0543, found 441.0541.

**Minor diastereomer (faster running).**

**^1^H NMR** (400 MHz, CDCl_3_): δ 6.97 (s, 1H, *H*-6′), 6.88 (s, 1H, *H*-3′), 6.01–6.56 (m, 2H, O-C*H*_2_-O), 5.84 (ddt, 1H, *J* = 17.2, 10.2, 6.9, C*H*=CH_2_), 5.15 (d, 1H, *J* = 3.8, *C*-6), 5.09–4.97 (m, 3H, CH=C*H*_2_, C*H*-CH_2_C=), 4.69 (t, 1H, *J* = 4.5, *H*-3a), 4.45 (d, 1H, *J* = 4.3, *H*-6a), 4.10 (dd, 1H, *J* = 10.8, 3.7, *H*-5), 4.01 (d, 1H, *J* = 10.8, *H*-5), 3.84 (td, 1H, *J* = 7.1, 4.7, *H*-3), 3.71 (dd, 1H, *J* = 8.9, 6.7, *H*-2), 3.57 (dd, 1H, *J* = 8.8, 7.6, *H*-2), 2.54 (dt, 1H, *J* = 14.1, 7.1, C*H*H-C=), 2.41 (dddt, 1H, *J* = 13.2, 6.8, 6.0, 2.8, C*H*H-C=), 2.07 (s, 3H, C*H*_3_). **^13^C NMR** (101 MHz, CDCl_3_) δ 170.1 (*C*=O), 148.0 (*C*-2a′, *C*-6a′), 133.9 (*C*-4′, *C*H=CH_2_), 117.3 (CH=*C*H_2_), 113.8 (*C*-5′), 112.3 (*C*-3′), 107.3 (*C*-6′), 101.8 (O-*C*H_2_O), 85.9 (*C*-6a), 80.4 (*C*-3a), 79.5 (*C*H-CH_2_C=), 78.5 (*C*-6), 77.6 (*C*-3), 73.8 (*C*-5), 70.5 (*C*-2), 41.5 (*C*H_2_-CH=), 21.0 (*C*H_3_).

**(3*R*,3a*R*,6*S*,6a*S*)-3-[(1-(*R*,*S*)-(3,4,5-trimethoxyphenyl)but-3-en-1-yl)oxy]-6-((*tert*-butyldiphenylsilyl)oxy)hexahydrofuro[3,2-*b*]furan 16b**. It is prepared in 96% yield from 3,4,5-trimethoxybenzaldehyde following the same procedure used for **16a** (reaction time: 1 h). The two diastereomers are not separable. The d.r. (determined on the crude product or on the chromatographed product) was = 76:24 (A = major diast.; B = minor diast.).

Colorless oil. R*_f_*: 0.26 (PE/EtOAc 5:1). **[α]_D_**: + 15.7 (c 1, CHCl_3_). **^1^H NMR** (400 MHz, CDCl_3_): δ 7.66–7.54 (A + B) (m, 4H, C*H* meta of TDBDPS), 7.46–7.31 (A + B) (m, 6H, C*H* ortho and para of TBDPS), 6.62 (A + B) (s, 2H, *H*-2′, *H*-6′), 5.72 (A + B) (ddt, 1H, *J* = 17.2, 10.2, 6.9, C*H*=CH_2_), 5.08–4.94 (m, 2H, CH=C*H*_2_), 4.68 (t, 0.24H, *J* = 4.7, *H*-3a (B)), 4.48–4.15 (m, 3.76H, *H*-3a (A), *H*-6a (A + B), *H*-6 (A + B), C*H*-CH_2_C = (A + B)), 3.88 (s, 6H, OC*H*_3_), 3.85 (s, 3H, OC*H*_3_), 3.92–3.71 (m, 2.52 H, 1 *H*-2 (A), *H*-3 (A + B), 1 *H*-5 (A)), 3.65 (dd, 0.24H, *J* = 9.7, 3.1, 1 *H*-5 (B)), 3.53 (dd, 0.24H, *J* = 8.4, 6.8, *H*-2 (B)), 3.50–3.42 (m, 0.76H, *H*-2 (A)), 3.40 (dd, 0.24H, *J* = 9.1, 6.3, *H*-2 (B)), 2.67–2.57 (A + B) (m, 1H, C*H*H-CH=), 2.43–2.30 (A + B) (m, 1H, C*H*H-CH=), 1.06 (B), 1.01 (A) (s, 9H, C(C*H*3)3). **^13^C NMR** (101 MHz, CDCl_3_) δ 153.2 (*C*-3′, *C*-4′, *C*-5′) (A + B), 137.5 (A), 137.3 (B) (*C*-1′), 135.6 (*C*H ortho to Si), 134.7 (A + B) (*C*H=CH_2_) 133.5 (A), 133.3 (B), 133.2 (A), 133.1 (B) (quat. of TBDPS), 130.00 (B), 129.96 (B), 129.90 (A), 129.88 (A) (*C*H para to Si), 127.9 (B), 127.8 (B), 127.8 (A), 127.7 (A) (*C*H meta to Si), 117.1 (A + B) (CH=*C*H_2_), 103.5 (A + B) (*C*-2′, *C*-6′), 88.2 (B), 88.1 (A) (*C*-6a), 83.5 (A + B) (*C*-3a), 81.7 (B), 80.7 (A) (*C*H-CH_2_C=), 78.9 (*C*-3), 78.3 (A), 78.1 (B) (*C*-6), 76.0 (A), 75.8 (B) (*C*-5), 73.4 (B), 68.6 (A) (*C*-2), 60.9 (A + B) (O*C*H_3_ at C-4′), 56.1 (A + B) (O*C*H_3_ at C-3′, C-5′), 42.6 (A + B) (*C*H_2_-CH=), 26.8 (A + B) (C(*C*H3)3), 19.1 (A + B) (*C*(CH3)3). **HRMS** (ESI+): calcd. for C_35_H_45_O_7_Si^+^ [M + H]+ 606.29291, found 606.2940.

**(3*R*,3a*R*,6*S*,6a*S*)-3-[(1-(*R*,*S*)-(4-chlorophenyl)but-3-en-1-yl)oxy]-6-((*tert*-butyldiphenylsilyl)oxy)hexahydrofuro[3,2-*b*]furan 16c**. It is prepared in 76% overall yield from 4-chlorobenzaldehyde following the same procedure used for **16a** (reaction time: 1.5 h). The two diastereomers are separable by chromatography (PE/EtOAc 10:1). The d.r. (determined on the crude product) was = 88:12. Colorless oil. R*_f_*: 0.27 (major), 0.36 (minor) (PE/EtOAc 10:1). Only the major diastereomer was fully characterized.

**Major diastereomer (slower running).**

**[α]_D_**: + 9.3 (c 1, CHCl_3_).**^1^H NMR** (400 MHz, CDCl_3_): δ 7.62–7.57 (m, 4H, C*H* meta of TDBDPS), 7.46–7.27 (m, 10H, C*H* ortho and para of TBDPS, *H*-2′, *H*-3′), 5.66 (ddt, 1H, *J* = 17.2, 10.3, 7.1, C*H*=CH_2_), 5.03–4.95 (m, 2H, CH=C*H*_2_), 4.35 (t, 1H, *J* = 3.8, *H*-3a), 4.29–4.23 (m, 3H, C*H*-CH_2_C=, *H*-6a, *H*-6), 3.89–3.79 (m, 3H, *H*-5 (1), *H*-2 (1), *H*-3), 3.74 (dd, 1H, *J* = 9.7, 3.5, *H*-5), 3.51–3.44 (m, 1H, *H*-2), 2.62 (dt, 1H, *J* = 13.8, 6.8, C*H*HC=), 2.37 (dt, 1H, *J* = 14.0, 6.9, C*H*HC=), 1.03 (s, 9H, C(C*H*3)3).**^13^C NMR** (101 MHz, CDCl_3_) δ 140.1 (*C*-1′), 135.6 (*C*H ortho to Si), 134.0 (*C*H=CH_2_), 133.6, 133.4, 133.2 (quat. of TBDPS, *C*-4′), 129.9 (*C*H para to Si), 128.6, 128.3 (*C*-2′, *C*-3′), 127.8 (*C*H meta to Si), 117.5 (CH=*C*H_2_), 88.1 (*C*-6a), 82.1 (*C*H-CH_2_C=), 80.8 (*C*-3a), 78.5 (*C*-3), 78.2 (*C*-6), 76.1 (*C*-5), 68.7 (*C*-2), 42.2 (*C*H_2_-CH=), 26.8 (C(*C*H3)3), 19.1 (*C*(CH3)3). **HRMS** (ESI+): calcd. for C_32_H_38_ClO_4_Si^+^ [M + H]+ 549.22224, found 549.2223.

**Minor diastereomer (faster running).**

**^1^H NMR** (400 MHz, CDCl_3_): δ 7.65–7.59 (m, 4H, C*H* meta of TDBDPS), 7.47–7.34 (m, 6H, C*H* ortho and para of TBDPS), 7.31 (d, 2H, *J* = 8.4, *H*-3′, *H*-5′), 7.11 (d, 2H, *J* = 8.4, *H*-2′, *H*-6′), 5.69 (ddt, 1H, *J* = 17.3, 10.3, 7.0, C*H*=CH_2_), 5.05–4.93 (m, 2H, CH=C*H*_2_), 4.67 (t, 1H, *J* = 4.2, *H*-3a), 4.52 (t, 1H, *J* = 6.8, C*H*-CH_2_CH=), 4.29 (d, 1H, *J* = 4.0, *H*-6a), 4.27–4.25 (m, 1H, *H*-6), 3.88 (d, 1H, *J* = 9.8, *H*-5), 3.84–3.75 (m, 2H, *H*-5, *H*-3), 3.52 (dd, 1H, *J* = 8.6, 6.9, *H*-2), 3.39 (t, 1H, *J* = 8.4, *H*-2), 2.64 (dtt, 1H, *J* = 13.6, 6.8, 1.2, C*H*H-CH=), 2.64 (dtt, 1H, *J* = 13.6, 6.8, 1.2, C*H*H-CH=), 2.64 (dtt, 1H, *J* = 14.4, 7.2, 1.2, C*H*H-CH=), 1.06 (s, 9H, C(C*H*3)3). **^13^C NMR** (101 MHz, CDCl_3_) δ 140.1 (*C*-1′), 135.7, 135.6 (*C*H ortho to Si), 134.0 (*C*H=CH_2_), 133.6, 133.4, 133.2 (quat. of TBDPS, *C*-4′), 129.91, 129.90 (*C*H para to Si), 128.7, 128.3 (*C*-2′, *C*-3′), 127.80, 127.78 (*C*H meta to Si), 117.4 (CH=*C*H_2_), 88.4 (*C*-6a), 80.9 (*C*H-CH_2_C=), 80.0 (*C*-3a), 78.1 (*C*-6), 78.0 (*C*-3), 76.2 (*C*-5), 69.9 (*C*-2), 42.4 (*C*H_2_-CH=), 26.8 (C(*C*H3)3), 19.1 (*C*(CH3)3).

**(3*R*,3a*R*,6*S*,6a*S*)-3-[(1-(*R*,*S*)-(3-Methoxyphenyl)but-3-en-1-yl)oxy]-6-((*tert*-butyldiphenylsilyl)oxy)hexahydrofuro[3,2-*b*]furan 16d**. It is prepared in 80% overall yield from 3-methoxybenzaldehyde following the same procedure used for **16a** (reaction time: 2 h). The d.r. (determined on the chromatographed product as the mixture of unseparated diastereomers) was = 90:10. Colorless oil. R*_f_*: 0.38 (major), 0.41 (minor) (PE/EtOAc 10:1). A careful second chromatography allows for obtaining diastereomerically pure samples of both diastereomers. Only the major diastereomer was fully characterized.

**Major diastereomer (slower running).**

**[α]_D_**: + 4.6 (c 1, CHCl_3_). **^1^H NMR** (400 MHz, CDCl_3_): δ 7.64–7.56 (m, 4H, C*H* meta of TDBDPS), 7.46–7.31 (m, 6H, C*H* ortho and para of TBDPS), 7.27 (t, 1H, *J* = 7.8, *H*-5′), 6.97 (dd, 1H, *J* = 2.4, 1.6, *H*-2′), 6.92 (dt, 1H, *J* = 7.5, 1.2, *H*-6′), 6.85 (ddd, 1H, *J* = 8.2, 2.6, 0.9, *H*-4′), 5.71 (ddt, 1H, *J* = 17.2, 10.2, 7.0, C*H*=CH_2_), 5.05–4.94 (m, 2H, CH=C*H*_2_), 4.36 (t, 1H, *J* = 3.8, *H*-3a), 4.29–4.20 (m, 3H, C*H*-CH_2_C=, *H*-6a, *H*-6), 3.93–3.79 (m, 3H, *H*-5 (1), *H*-2 (1), *H*-3), 3.83 (s, 3H, OC*H*_3_), 3.76 (dd, 1H, *J* = 9.7, 3.5, *H*-5), 3.52–3.42 (m, 1H, *H*-2), 2.64 (dtt, 1H, *J* = 14.2, 7.0, 1.2, C*H*HC=), 2.37 (dtt, 1H, *J* = 14.0, 7.0, 1.2, C*H*HC=), 1.02 (s, 9H, C(C*H*3)3). **^13^C NMR** (101 MHz, CDCl_3_) δ 159.8 (*C*-3′), 143.2 (*C*-1′), 135.62, 135.61 (*C*H ortho to Si), 134.5 (*C*H=CH_2_), 133.5, 133.2 (quat. of TBDPS), 129.87, 129.84 (*C*H para to Si), 129.4 (*C*-5′), 127.8, 127.7 (*C*H meta to Si), 119.3 (*C*-6′), 117.1 (CH=*C*H_2_), 113.7 (*C*-4′), 111.9 (*C*-2′), 88.0 (*C*-6a), 82.9 (*C*H-CH_2_C=), 80.9 (*C*-3a), 78.4 (*C*-3), 78.2 (*C*-6), 76.0 (*C*-5), 68.6 (*C*-2), 55.3 (O*C*H_3_), 42.3 (*C*H_2_-CH=), 26.8 (C(*C*H3)3), 19.1 (*C*(CH3)3). **HRMS** (ESI+): calcd. for C_33_H_41_O_5_Si^+^ [M + H]+ 545.27178, found 545.2716.

**Minor diastereomer (faster running).**

**^1^H NMR** (400 MHz, CDCl_3_): δ 7.66–7.59 (m, 4H, C*H* meta of TDBDPS), 7.48–7.33 (m, 6H, C*H* ortho and para of TBDPS), 7.25 (t, 1H, *J* = 7.8, *H*-5′), 6.90–6.79 (m, 3H, *H*-2′, *H*-4′, *H*-6′), 5.74 (ddt, 1H, *J* = 17.2, 10.2, 6.9, C*H*=CH_2_), 5.08–4.93 (m, 2H, CH=C*H*_2_), 4.68 (t, 1H, *J* = 4.2, *H*-3a), 4.51 (t, 1H, *J* = 6.9, C*H*-CH_2_C=), 4.29 (d, 1H, *J* = 4.0, *H*-6a), 4.28–4.23 (m, 1H, *H*-6), 3.92–3.75 (m, 3H, *H*-5 (2), *H*-3), 3.80 (s, 3H, OC*H*_3_), 3.52 (dd, 1H, *J* = 8.6, 7.0, *H*-2), 3.40 (t, 1H, *J* = 8.4, *H*-2), 2.65 (dtt, 1H, *J* = 14.0, 6.9, 1.2, C*H*HC=), 2.41 (dtt, 1H, *J=* 14.0, 6.9, 1.2, C*H*HC=), 1.06 (s, 9H, C(C*H*3)3). **^13^C NMR** (101 MHz, CDCl_3_) δ 159.7 (*C*-3′), 143.3 (*C*-1′), 135.7 (*C*H ortho to Si), 134.5 (*C*H=CH_2_), 133.4, 133.3 (quat. of TBDPS), 129.9 (*C*H para to Si), 129.5 (*C*-5′), 127.8 (*C*H meta to Si), 119.4 (*C*-6′), 117.0 (CH=*C*H_2_), 113.2 (*C*-4′), 112.4 (*C*-2′), 88.4 (*C*-6a), 81.6 (*C*H-CH_2_C=), 80.0 (*C*-3a), 78.2 (*C*-6), 77.9 (*C*-3), 76.2 (*C*-5), 69.8 (*C*-2), 55.2 (O*C*H_3_), 42.4 (*C*H_2_-CH=), 26.8 (C(*C*H3)3), 19.1 (*C*(CH3)3).

**(3*R*,3a*R*,6*S*,6a*S*)-3-[(1-(*R*,*S*)-(2-Chloro-6-fluorophenyl)but-3-en-1-yl)oxy]-6-((*tert*-butyldiphenylsilyl)oxy)hexahydrofuro[3,2-*b*]furan 16e**. It is prepared in 37% overall yield from 2-chloro-6-fluorobenzaldehyde following the same procedure used for **16a** (reaction time: 4 h). The diastereomers are not separable by chromatography. The d.r. (determined on the chromatographed product as the mixture of unseparated diastereomers) was = 88:12. Colorless oil. R*_f_*: 0.40 (PE/EtOAc 10:1). In this case, also, the product of bicomponent addition of allyl trimethylsilane to the starting aldehyde is obtained in 48% yield.

**[α]_D_**: + 19.6 (c 0.83, CHCl_3_) (measured on the diastereomeric mixture). **^1^H NMR** (400 MHz, CDCl_3_) (**only the signals of major diastereomer are reported**): δ 7.67–7.55 (m, 4H, C*H* meta of TDBDPS), 7.46–7.31 (m, 6H, C*H* ortho and para of TBDPS), 7.31–7.14 (m, 2H, *H*-3′, *H*-4′), 7.02 (ddd, 1H, *J*= 9.5, 8.0, 1.3, *H*-5′), 5.70 (ddt, 1H, *J* = 17.2, 10.2, 7.3, C*H*=CH_2_), 5.08–4.93 (m, 3H, CH=C*H*_2_, C*H*-CH_2_CH=), 4.34 (t, 1H, *J* = 3.7, *H*-3a), 4.29 (d, 1H, *J* = 3.4, *H*-6a), 4.22 (mc) (m, 1H, *H*-6), 3.97–3.85 (m, 2H, *H*-2, *H*-3), 3.79 (d, 1H, *J* = 10.0, *H*-5), 3.73 (dd, 1H, *J* = 9.8, 3.6, *H*-5), 3.50 (t, 1H, *J* = 7.6, *H*-2), 2.89 (dt, 1H, *J* = 13.5, 6.6, C*H*HC=), 2.69 (dt, 1H, *J* = 14.9, 8.1, C*H*HC=), 1.02 (s, 9H, C(C*H*3)3). **^13^C NMR** (101 MHz, CDCl_3_) (only the signals of major diastereomer are reported): δ 162.3 (d, *J* = 253 Hz, *C*-6′), 135.6 (*C*H ortho to Si), 134.8 (d, *J* = 6.8, *C*-2′), 133.7 (*C*H=CH_2_), 133.5, 133.3 (quat. of TBDPS), 129.86, 129.84 (*C*H para to Si), 129.8 (d, partially covered by the signals of *C*H para to Si) (*C*-3′), 127.76, 127.73 (*C*H meta to Si), 125.6 (*C*-4′), 117.7 (CH=*C*H_2_), 115.2 (d, *J* = 20.2, *C*-5′), 88.2 (*C*-6a), 80.8 (*C*-3a), 79.0 (*C*-3), 78.2 (*C*-6), 77.0 (covered by central CDCl_3_ signal, *C*H-CH_2_C=), 76.1 (*C*-5), 68.6 (*C*-2), 38.6 (*C*H_2_-CH=), 26.8 (C(*C*H3)3), 19.1 (*C*(CH3)3). **HRMS** (ESI+): calcd. for C_32_H_37_ClFO_4_Si^+^ [M + H]+ 567.21282, found 567.2129.

**Selected ^1^H NMR signals of minor diastereomer**: 5.27 (t, 1H, *J* = 7.6, C*H*-CH_2_CH=), 4.76 (t, 1H, *J* = 4.2, *H*-3a), 3.57 (dd, 1H, *J* = 8.4, 7.2, *H*-2), 3.42 (t, 1H, *J* = 8.4, *H*-2), 1.06 (s, 9H, C(C*H*3)3).

**^1^H NMR** data of side product **(*R*,*S*)-1-(2-Chloro-6-fluorophenyl)-but-3-en-1-ol**: ^1^H NMR (400 MHz, CDCl_3_): δ 7.23–7.14 (m, 2H, aromatic C*H*), 7.04–6.95 (m, 1 H, aromatic C*H*), 5.82 (ddtd, 1 H, *J* = 17.3, 10.2, 7.2, 1.0, C*H*=CH_2_), 5.28 (td, 1H, *J* = 8.4, 6.8, C*H*-OH), 5.19–5.07 (m, 2H, CH=C*H*_2_), 2.82–2.72 (m, 1H, C*H*H-CH=), 2.68–2.58 (m, 1H, C*H*H-CH=), 2.50 (dd, 1H, *J* = 9.1, 4.6, O*H*).

**(3*R*,3a*R*,6*S*,6a*S*)-3-[(1-(*R*,*S*)-(4-nitrophenyl)but-3-en-1-yl)oxy]-6-((*tert*-butyldiphenylsilyl)oxy)hexahydrofuro[3,2-*b*]furan 16f**. It is prepared in 30% overall yield from 4-nitrobenzaldehyde following the same procedure used for **16a** (reaction time: 2 h). **Only the pure major diastereomer was isolated in pure form**. Colorless oil. R*_f_*: 0.23 (PE/EtOAc 5:1). Colorless oil. Examination of the crude product showed some unreacted starting aldehyde (25%), as well as some product of a bicomponent reaction ((*R*,*S*)-1-(4-nitrophenyl)-but-3-en-1-ol). Anyway, this side product was not isolated. Also, some peaks of the minor diastereomer were detected. The d.r. (from the crude) is approximately = 92:8. **[α]_D_**: + 11.8 (c 1.07, CHCl_3_) (measured on the diastereomeric mixture). ^1^H NMR (400 MHz, CDCl_3_): δ 8.23 (d, 2H, *J* = 8.8, *H*-3′), 7.63–7.57 (m, 4H, C*H* meta of TDBDPS), 7.55 (d, 1H, *J* = 8.6, *H*-2′), 7.46–7.33 (m, 6H, C*H* ortho and para of TBDPS), 5.67 (ddt, 1H, *J* = 17.2, 10.5, 7.1, C*H*=CH_2_), 5.05–4.94 (m, 2H, CH=C*H*_2_), 4.46 (t, 1H, *J* = 6.5, C*H*-CH_2_CH=), 4.42 (t, 1H, *J* = 4.1, *H*-3a), 4.30 (d, 1H, *J* = 4.0, *H*-6a), 4.26 (mc) (m, 1H, *H*-6), 3.94–3.82 (m, 2H, *H*-2, *H*-3), 3.81 (d, 1H, *J* = 9.7, *H*-5), 3.73 (dd, 1H, *J* = 9.8, 3.5, *H*-5), 3.53 (t, 1H, *J* = 8.1, *H*-2), 2.62 (dtt, 1H, *J* = 13.8, 6.9, 0.9, C*H*HC=), 2.42 (mc, 1H, C*H*HC=), 1.03 (s, 9H, C(C*H*3)3). **^13^C NMR** (101 MHz, CDCl_3_) (only the signals of the major diastereomer are reported): δ 149.2, 147.6 (*C*-1′, *C*-4′), 135.6 (*C*H ortho to Si), 133.3, 133.2 (quat. of TBDPS), 133.1 (*C*H=CH_2_), 129.9 (*C*H para to Si), 127.79, 127.76 (*C*H meta to Si), 127.6 (*C*-2′), 123.7 (*C*-3′) 118.2 (CH=*C*H_2_), 88.3 (*C*-6a), 81.6 (*C*-3a), 80.6 (*C*H-CH_2_C=), 79.2 (*C*-3), 78.1 (*C*-6), 76.1 (*C*-5), 69.1 (*C*-2), 42.0 (*C*H_2_-CH=), 26.8 (C(*C*H3)3), 19.1 (*C*(CH3)3). **HRMS** (ESI+): calcd. for C_32_H_38_NO_6_Si^+^ [M + H]+ 560,24629, found 560.2459.

**(3*R*,3a*R*,6*S*,6a*S*)-3-[(1-(*R*,*S*)-(thien-3-yl)but-3-en-1-yl)oxy]-6-((*tert*-butyldiphenylsilyl)oxy)hexahydrofuro[3,2-*b*]furan 16g**. It is prepared in 95% overall yield from 3-thiophenecarboxyaldehyde following the same procedure used for **16a** (reaction time: 1 h). The d.r. (determined on the crude product) was = 82:17. Colorless oil. R*_f_*: 0.28 (major), 0.35 (minor) (PE/EtOAc 10:1). Only the major diastereomer was isolated in pure form and fully characterized, whereas the minor diastereomer was isolated only as an enriched mixture. Yield: 78% (on the pure diastereomer).

**Major diastereomer (slower running).**

**[α]_D_**: + 12.1 (c 1, CHCl_3_). **^1^H NMR** (400 MHz, CDCl_3_): δ 7.64–7.56 (m, 4H, C*H* meta of TDBDPS), 7.46–7.34 (m, 6H, C*H* ortho and para of TBDPS), 7.33 (dd, 1H, *J* = 5.0, 3.0, *H*-5′), 7.23 (dd, 1H, *J* = 2.9, 1.2, *H*-2′), 7.14 (dd, 1H, *J* = 5.0, 1.2, *H*-4′), 5.71 (ddt, 1H, *J* = 17.2, 10.2, 7.0, C*H*=CH_2_), 5.06–4.96 (m, 2H, CH=C*H*_2_), 4.42 (t, 1H, *J* = 6.8, C*H*-CH_2_CH=), 4.34 (t, 1H, *J* = 3.9, *H*-3a), 4.29 (d, 1H, *J* = 3.7, *H*-6a), 4.24 (mc, 1H, *H*-6), 3.93 (ddd, 1H, *J* = 8.9, 6.9, 4.2, *H*-3), 3.87–3.79 (m, 2H, 1 *H*-2, 1 *H*-5), 3.75 (dd, 1H, *J* = 9.7, 3.7, *H*-5), 3.44 (t, 1H, *H* = 8.6, *H*-2), 2.67 (dtt, 1H, *J* = 14.0, 7.0, 0.9, C*H*HC=), 2.47 (dtt, 1H, *J* = 14.0, 7.0, 0.9, C*H*HC=), 1.03 (s, 9H, C(C*H*3)3). **^13^C NMR** (101 MHz, CDCl_3_) δ 143.0 (*C*-1′), 135.6 (*C*H ortho to Si), 134.4 (*C*H=CH_2_), 133.5, 133.3 (quat. of TBDPS), 129.87, 129.85 (*C*H para to Si), 127.8, 127.7 (*C*H meta to Si), 126.22, 126.17 (*C*-4′, *C*-5′), 122.4 (*C*-2′), 117.2 (CH=*C*H_2_), 88.1 (*C*-6a), 80.8 (*C*-3a), 78.5 (*C*H-CH_2_C=), 78.4 (*C*-3), 78.2 (*C*-6), 76.1 (*C*-5), 68.7 (*C*-2), 41.3 (*C*H_2_-CH=), 26.8 (C(*C*H3)3), 19.1 (*C*(CH3)3). **HRMS** (ESI+): calcd. for C_30_H_37_O_4_SSi^+^ [M + H]+ 521.21763, found 521.2177.

**Selected ^1^H NMR signals of minor diastereomer**: 4.68 (t, 1H, *J* = 4.4, *H*-3a), 4.64 (t, 1H, *J* = 6.8, C*H*-CH_2_CH=), 3.50 (t, 1H, *J* = 7.6, *H*-2), 3.35 (t, 1H, *J* = 8.4, *H*-2), 1.06 (s, 9H, C(C*H*3)3).

**(3*R*,3a*R*,6*S*,6a*S*)-3-[(1-(*R*,*S*)-(3-bromobenzothiophen-2-yl)but-3-en-1-yl)oxy]-6-((*tert*-butyldiphenylsilyl)oxy)hexahydrofuro[3,2-*b*]furan 16h**. It is prepared in 71% overall yield from 3-bromobenzothiophene-2-carboxyaldehyde following the same procedure used for **16a**. However, after 1 h at −78 °C, the temperature is allowed to reach −40 °C in 1 h. The d.r. (determined on the crude product) was = 91:9. R*_f_*: 0.27 (major), 0.35 (minor) (PE/EtOAc 10:1). Also, a small amount (5%) of the bicomponent product ((*R*,*S*)-1-(3-bromobenzothiophen-2-yl)-but-3-en-1-ol) is obtained. Only the major diastereomer was isolated in pure form and fully characterized, whereas the minor diastereomer was isolated only as an enriched mixture. Yield: 65% (on the pure diastereomer).

**[α]_D_**: −0.38 (c 1, CHCl_3_). **^1^H NMR** (400 MHz, CDCl_3_): δ 7.84 (ddd, 1H, *J* = 8.0, 1.2, 0.8, *H*-4′ or *H*-7′), 7.80 (ddd, 1H, *J* = 8.0, 1.2, 0.8, *H*-4′ or *H*-7′), 7.61–7.56 (m, 4H, C*H* meta of TDBDPS), 7.49–7.33 (m, 8H, C*H* ortho and para of TBDPS, *H*-5′, *H*-6′), 5.76 (dddd, 1H, *J* = 17.2, 10.4, 7.6, 6.8, C*H*=CH2), 5.09 (dq, 1H, *J* = 17.2, 1.7, CH=C*H*H), 5.02 (dtd, 1H, *J* = 10.4, 2.0, 1.2, CH=C*H*H), 4.94 (t, 1H, *J* = 6.9, C*H*-CH_2_CH=), 4.51 (t, 1H, *J* = 3.9, *H*-3a), 4.29 (d, 1H, *J* = 3.8, *H*-6a), 4.25 (mc) (m, 1H, *H*-6), 4.02 (ddd, 1H, *J* = 8.2, 6.7, 4.1, *H*-3), 3.97 (dd, 1H, *J* = 8.0, 6.8, *H*-2), 3.85 (d, 1H, *J* = 9.9, *H*-5), 3.76 (dd, 1H, *J* = 9.8, 3.6, *H*-5), 3.52 (t, 1H, *J* = 8.0, *H*-2), 2.79 (dtt, 1H, *J* = 13.8, 6.8, 1.2, C*H*H-CH=), 2.60 (dtt, 1H, *J* = 13.8, 7.2, 1.2, C*H*H-CH=), 1.01 (s, 9H, C(C*H*3)3). **^13^C NMR** (101 MHz, CDCl_3_) δ 141.4 (*C*-7a′), 137.8, 137.7 (*C*-3a′, *C-*2′), 135.6 (*C*H ortho to Si), 133.5, 133.2 (quat. of TBDPS), 133.0 (*C*H=CH_2_), 129.9, 129.8 (*C*H para to Si), 127.8, 127.7 (*C*H meta to Si), 125.7 (*C*-5′), 125.1 (*C*-6′), 123.1 (*C*-4′), 122.9 (*C*-7′), 118.2 (CH=*C*H_2_), 107.1 (*C*-3′), 88.1 (*C*-6a), 81.0 (*C*-3a), 78.4 (*C*-3), 78.1 (*C*-6), 77.4 (*C*H-CH_2_CH=, covered by CDCl_3_, detected at HSQC), 76.2 (*C*-5), 68.7 (*C*-2), 41.6 (*C*H_2_CH=), 26.8 (C(*C*H_3_)_3_), 19.1 (*C*(CH_3_)_3_). **HRMS** (ESI+): calcd. for C_34_H_38_BrO_4_SSi^+^ [M + H]+ 649.14380, found 649.1440.

**Selected ^1^H NMR signals of minor diastereomer**. ^1^H NMR (400 MHz, CDCl_3_): δ 5.81 (ddt, 1H, *J* = 17.2, 10.4, 7.2, C*H*=CH2), 5.26 (t, 1H, *J* = 6.9, C*H*-CH_2_C=), 5.09 (dq, 1H, *J* = 17.2, 1.6, CH=C*H*H), 5.01 (dtd, 1H, *J* = 10.4, 2.0, 1.2, CH=C*H*H), 4.81 (t, 1H, *J* = 4.2, *H*-3a), 4.33 (d, 1H, *J* = 4.1, *H*-6a), 4.28 (mc) (m, 1H, *H*-6), 3.98 (td, 1H, *J* = 7.1, 4.5, *H*-3), 3.90 (d, 1H, *J* = 9.8, *H*-5), 3.79 (dd, 1H, *J* = 9.8, 7.4, *H*- 5), 3.62 (dd, 1H, *J* = 8.8, 3.7, *H*-2), 3.48 (dd, 1H, *J* = 8.8, 7.7, *H*-2), 2.86 (dtt, 1H, *J* = 14.0, 6.8, 1.2, *H*-2′), 2.60 (dtt, 1H, *J* = 13.8, 7.0, 1.2, *H*-2′), 1.07 (s, 9H, C(C*H*3)3).

**(3*R*,3a*R*,6*S*,6a*S*)-3-[(1-(*R*,*S*)-(2-Benzyloxyphenyl)but-3-en-1-yl)oxy]-6-((*tert*-butyldiphenylsilyl)oxy)hexahydrofuro[3,2-*b*]furan 16i**. It is prepared in 45% overall yield from 2-benzyloxybenzaldehyde following the same procedure used for **16a**. However, after 1 h at −78 °C, the temperature is allowed to reach −40 °C in 1 h. The d.r. (determined on the crude product) was = 79:21. The two diastereomers cannot be separated. R*_f_*: 0.56 (PE/EtOAc 10:1). **HRMS** (ESI+): calcd. for C_39_H_45_O_5_Si^+^ [M + H]+ 621.30308, found 621.3028.

**Major diastereomer.**

**^1^H NMR** (400 MHz, CDCl_3_): δ 7.65–7.55 (m, 5H, C*H* meta of TDBDPS, *H*-6′), 7.45–7.28 (m, 11H, C*H* ortho and para of TBDPS, C*H* of benzyl), 7.25 (td, 1H, *J* = 7.8, 2.0, *H*-4′), 7.05 td, 1H, *J* = 7.5, 0.9, *H*-5′), 6.93 (d, 1H, *J* = 8.2, *H*-3′), 5.80 (dddd, 1H, *J* = 17.2, 10.4, 7.6, 6.8, C*H*=CH2), 5.09, 5.07 (AB system, 2H, *J* = 11.8, C*H*2Ph), 5.01–4.92 (m, 2H, CH=C*H*2), 4.86 (dd, 1H, *J* = 7.7, 5.3, C*H*-CH_2_CH=), 4.44 (t, 1H, *J* = 3.8, *H*-3a), 4.30–4.22 (m, 2H, *H*-6a, *H*-6), 3.87–3.79 (m, 3H, *H*-5, *H*-2, *H*-3), 3.77 (dd, 1H, *J* = 9.7, 3.5, *H*-5), 3.47 (mc) (m, 1H, *H*-2), 2.59–2.39 (m, 2H, *H*-2′), 1.03 (s, 9H, C(C*H*3)3). **^13^C NMR** (101 MHz, CDCl_3_) δ 155.9 (*C*-2′), 137.1 (quat. benzyl), 135.6 (*C*H ortho to Si), 135.2 (*C*H=CH2), 133.5, 133.3 (quat. of TBDPS), 130.5 (*C*-1′), 129.9 (*C*H para to Si), 128.6 (*C*H meta of Bn), 128.4 (*C*-4′), 127.8, 127.7 (*C*H meta to Si), 127.2 (*C*H ortho of Bn + *C*-6′), 121.2 (*C*-5′), 116.6 (CH=*C*H2), 111.6 (*C*-3′), 88.1 (*C*-6a), 81.0 (*C*-3a), 78.7 (*C*-3), 78.3 (*C*-6), 76.1 (*C*-5 + *C*H-CH_2_C=C), 70.0 (PhC*H*2), 68.9 (*C*-2), 41.3 (*C*H_2_-CH=), 26.8 (C(*C*H3)3), 19.1 (*C*(CH3)3).

**Selected signals of minor diastereomer**. ^1^H NMR (400 MHz, CDCl_3_): δ 6.96 (td, 1H, *J* = 7.5, 0.9, *H*-5′), 5.10 (s, 2H, C*H*_2_Ph), 4.66 (t, 1H, *J* = 4.0, *H*-3a), 3.54 (dd, 1H, *J* = 8.3, 7.1, *H*-2), 3.39 (t, 1H, *J* = 8.5, *H*-2), 1.05 (s, 9H, C(C*H*3)3). ^13^C NMR (101 MHz, CDCl_3_) δ 88.3 (*C*-6a), 80.0 (*C*-3a), 74.8 (*C*H-CH_2_C=C), 69.4 (*C*-2).

**(3*R*,3a*R*,6*S*,6a*S*)-3-[(1-(*R*,*S*)-cyclohexylbut-3-en-1-yl)oxy]-6-((*tert*-butyldiphenylsilyl)oxy)hexahydrofuro[3,2-*b*]furan 16k**. It is prepared in 60% overall yield from cyclohexanecarboxaldehyde. following the same procedure used for **16a**. However, after 2 h at −78 °C, the temperature is allowed to reach −40 °C in 1 h. The d.r. (determined on the crude product or on the chromatographed product) was = 66:34 (integration of the signals of C*H*=CH_2_ at 5.79 (major) or 5.96 (minor) ppm). The two diastereomers cannot be separated. R*_f_*: 0.42 (PE/EtOAc 9:1). **HRMS** (ESI+): calcd. for C_32_H_45_O_4_Si^+^ [M + H]+ 521.30816, found 521.3089.

**Major diastereomer.**

**^1^H NMR** (400 MHz, CDCl_3_): δ 7.67–7.60 (m, 4 H), 7.46–7.34 (m, 6H), 5.79 (ddt, 1H, *J* = 17.2, 10.2, 7.0 Hz, C*H*=CH_2_), 5.08–4.98 (m, 2H, CH=C*H*_2_), 4.62 (t, 1H, *J* = 4.0 Hz, *H*-3a), 4.37 (d, 1H, *J* = 4.0, *H*-6a), 4.26 (mc) (m, 1H, *H*-6), 4.03–3.96 (m, 1H, *H*-3), 3.90–3.77 (m, 2H, *H*-2 + *H*-5), 3.75 (dd, 1H, *J* = 9.7, 3.6, *H*-5), 3.37 (dd, 1H, *J* = 9.6, 8.2, *H*-2), 3.18 (q, 1H, *J* = 6.0 Hz, C*H*-CH_2_CH=), 2.30–2.15 (m, 2H, C*H*_2_CH=CH_2_), 1.98–1.85 (m, 1 H, C*H cy*Hex), 1.82–1.58 (m, 4 H, C*H*_2_ *cy*Hex), 1.55–1.09 (m, 6H, C*H*_2_ *cy*Hex), 1.06 (s, 9H, (C*H*_3_)_3_C).**^13^C NMR** (101 MHz, CDCl_3_) δ 135.7 (*C*H ortho to Si), 134.6 (*C*H=CH_2_), 133.3 (quat.), 129.88, 129.85 (*C*H para to Si), 127.8, 127.7 (*C*H meta to Si), 116.8 (CH=*C*H_2_), 88.1 (*C*-6a), 85.1 (*C*-1′), 80.6 (*C*-3), 80.3 (*C*-3a), 78.3 (*C*-6), 76.2 (*C*-5), 69.0 (*C*-2), 41.5 (*C*H of *cy*Hex), 35.7 (*C*H_2_CH=CH_2_), 28.9, 28.7, 26.6, 26.3, 26.2 (*C*H_2_ *cy*Hex), 26.8 (C(*C*H_3_)_3_), 19.1 (*C*(CH_3_)_3_).

**Selected signals of the minor diastereomer**.**^1^H NMR** (400 MHz, CDCl_3_): δ 5.96 (ddt, 1H, *J* = 17.2, 10.2, 7.1 Hz, C*H*=CH_2_), 4.63 (t, 1H, *J* = 4.0 Hz, *H*-3a), 4.37 (d, 1H, *J* = 4.0, *H*-6a), 4.26 (mc) (m, 1H, *H*-6), 4.03–3.96 (m, 1H, *H*-3), 3.90–3.77 (m, 3H, *H*-2 + *H*-5), 3.40 (t, 1H, *J* = 8.8, *H*-2), 3.14 (q, 1H, *J* = 6.0 Hz, C*H*-CH_2_CH=C), 2.40–2.27 (m, 2H, C*H*_2_CH=CH_2_), 1.06 (s, 9H, (C*H*_3_)_3_C).

**(3*R*,3a*R*,6*S*,6a*S*)-3-[(1-(*R*,*S*)-(2-methylprop-1yl)but-3-en-1-yl)oxy]-6-((*tert*-butyldiphenylsilyl)oxy)hexahydrofuro [3,2-*b*]furan 16l**. A solution of TMS ether **11** (245.2 mg, 537 µmol) in dry CH_2_Cl_2_ (2.5 mL) is treated with freshly distilled isovaleraldehyde (57 µL, 531 µmol) and with allyl trimethylsilane (127 µL, 800 µmol). The solution is cooled to −78 °C, and a freshly prepared 0.3 M solution of TMS-OTf in CH_2_Cl_2_ (716 µL, 215 µmol) is added. After 1 h, more isovaleraldehyde (28 µL, 261 µmol) and TMS-OTf solution (716 µL, 215 µmol) are added, and the solution is allowed to warm to −30 °C during 1 h. The reaction is quenched by the addition of Et_3_N (226 µL, 1.62 mmol). After warming, the mixture is diluted with a saturated aqueous solution of NaHCO_3_, extracted with AcOEt, dried, and evaporated to dryness. The crude product is chromatographed (PE/EtOAc 95:5) to give the diastereomeric mixture of **16l** (157 mg, 59%). The ratio (determined on the chromatographed product or on the crude product by ^1^H NMR) was = 88:12. Further elution with CH_2_Cl_2_/EtOAc 1:1 furnishes recovered alcohol **6** (63 mg, 30.5%). The yield based on non-recovered starting material (calculated as (µmol **16l**/(µmol starting **11** − µmol recovered **6**))) is = 85%. R*_f_*: 0.30 (PE/EtOAc 9:1). **[α]_D_**: + 44.3 (c 1, CHCl_3_). **HRMS** (ESI+): calcd. for C_30_H_43_O_4_Si^+^ [M + H]+ 495.29251, found 495.2928.

**Major diastereomer.**

**^1^H NMR** (400 MHz, CDCl_3_): δ 7.67–7.60 (m, 4 H), 7.46–7.34 (m, 6H), 5.78 (ddt, 1H, *J* = 17.4, 10.3, 7.1, C*H*=CH_2_), 5.08–5.00 (m, 2H, CH=C*H*_2_), 4.63 (t, 1H, *J* = 3.8, *H*-3a), 4.39 (d, 1H, *J* = 3.7, *H*-6a), 4.27 (mc) (m, 1H, *H*-6), 4.03 (ddd, 1H, *J* = 9.3, 7.2, 4.1, *H*-3), 3.86 (d, 1H, *J* = 9.3, *H*-5), 3.82 (dd, 1H, *J* = 8.0, 7.6, *H*-2), 3.75 (dd, 1H, *J* = 9.8, 3.6, *H*-5), 3.55 (dq, 1H, *J* = 8.2, 5.5 Hz, C*H*-CH_2_CH=), 3.39 (t, 1H, *J* = 8.6, *H*-2), 2.27–2.20 (m, 2H, C*H*_2_CH=CH_2_), 1.82 (t of oct, 1H, *J* = 8.0, 6.5), 1.52 (ddd, 1H, *J* = 14.1, 8.2, 5.8, C*H*H-*i*Pr), 1.29–1.21 (m, 1H, C*H*H-*i*Pr), 1.06 (s, 9H, (C*H*_3_)_3_C), 0.93 (d, 3H, *J* = 6.4, C*H*_3_CH), 0.91 (d, 3H, *J* = 6.4, C*H*_3_CH). **^13^C NMR** (101 MHz, CDCl_3_) δ 135.67, 135.65 (*C*H ortho to Si), 134.6 (*C*H=CH_2_), 133.5, 133.3 (quat.), 129.90, 129.87 (*C*H para to Si), 127.80, 127.75 (*C*H meta to Si), 117.2 (CH=*C*H_2_), 88.2 (*C*-6a), 80.4 (*C*-3a), 79.5 (*C*-3), 78.4 (*C*-6), 76.1 (*C*-5), 69.3 (*C*-2), 43.8 (*C*H_2_*i*Pr), 39.1 (*C*H_2_CH=CH_2_), 26.9 (C(*C*H_3_)_3_), 24.3 (*C*H(CH_3_)_2_), 23.2, 22.2 (*C*H_3_CH), 19.1 (*C*(CH_3_)_3_).

**Selected peaks of minor diastereomer.**

**^1^H NMR** (400 MHz, CDCl_3_): δ 5.87 (ddt, 1H, *J* = 16.8, 10.4, 7.2, C*H*=CH_2_), 4.40 (d, 1H, *J* = 3.7, *H*-6a), 3.82 (dd, 1H, *J*= 8.0, 7.6, *H*-2), 3.50–3.43 (m, 1H, C*H*-CH_2_CH=), 3.39 (t, 1H, *J*= 8.6, *H*-2), 2.42–2.25 (m, 2H, C*H*_2_CH=CH_2_), 0.88 (d, 3H, *J*= 6.8, C*H*_3_CH), 0.85 (d, 3H, *J*= 6.4, C*H*_3_CH). **^13^C NMR** (101 MHz, CDCl_3_) δ 135.67, 135.65 (*C*H ortho to Si), 134.8 (*C*H=CH_2_), 133.5, 133.3 (quat.), 129.90, 129.87 (*C*H para to Si), 127.80, 127.75 (*C*H meta to Si), 117.1 (CH=*C*H_2_), 88.3 (*C*-6a), 80.8 (*C*-3a), 79.9 (*C*-3), 78.4 (*C*-6), 76.1 (*C*-5), 69.0 (*C*-2), 43.4 (*C*H_2_*i*Pr), 39.6 (*C*H_2_CH=CH_2_), 26.9 (C(*C*H_3_)_3_), 24.3 (*C*H(CH_3_)_2_), 23.2, 22.4 (*C*H_3_CH), 19.1 (*C*(CH_3_)_3_).

**(3*R*,3a*R*,6*S*,6a*R*)-3-[(1-(*S*)-(5-bromobenzo[*d*][1,3]dioxole-5-yl)but-3-en-1-yl)oxy]-hexahydrofuro [3,2-*b*]furan-6-ol 18a**. A solution of **16a** (pure major diastereomer) (125.2 mg, 196 µmol) in dry THF (1 mL) is treated dropwise with tetrabutylammonum fluoride (TBAF) (1.0 M solution in THF) (392 µL, 392 µmol). The resulting brownish solution is stirred until the reaction is complete by TLC (50 min.). The solution is treated with saturated aqueous NH_4_Cl and extracted thrice with EtOAc. The organic extracts are washed with brine, dried and evaporated to give a crude product. Chromatography (PE/EtOAc 2:3) affords pure **18a** as an oil (58.9 mg, 75%). R*_f_*: 0.40 (PE/EtOAc 2:3). **[α]_D_**: −14.2 (c= 0.67, CHCl_3_) **^1^H NMR** (400 MHz, CDCl_3_): δ 7.12 (s, 1H, *H*-6′), 6.96 (s, 1H, *H*-3′), 6.00 (d, 1H, *J* = 1.0, O-C*H*H-O), 5.98 (d, 1H, *J* = 1.0, O-C*H*H-O), 5.81 (ddt, 1H, *J* = 17.2, 10.2, 7.0, C*H*=CH_2_), 5.11–4.99 (m, 2H, CH=C*H*_2_), 4.73 (dd, 1H, *J* = 7.8, 5.3, C*H*-CH_2_C=), 4.42 (t, 1H, *J* = 4.3, *H*-3a), 4.33 (d, 1H, *J* = 4.1, *H*-6a), 4.29 (d, 1H, *J* = 3.2, *H*-6), 4.00 (dd, 1H, *J* = 10.2, 3.5, *H*-5), 3.96–3.82 (m, 3H, *H*-3 + 1 *H*-2 + 1 *H*-5), 3.63 (t, 1H, *J* = 7.6, *H*-2), 2.49 (dt, 1H, *J* = 14.3, 7.1, C*H*HC=), 2.38 (dt, 1H, *J* = 14.1, 6.3, C*H*HC=). **^13^C NMR** (101 MHz, CDCl_3_) δ 147.92, 147.87 (*C*-2a′, *C*-6a′), 134.1 (*C*-4′, *C*H=CH_2_), 117.4 (CH=*C*H_2_), 113.1 (*C*-5′), 112.2 (*C*-3′), 107.8 (*C*-6′), 101.8 (O-*C*H_2_O), 88.0 (*C*-6a), 80.8 (*C*H-CH_2_C=), 80.6 (*C*-3a), 78.5 (*C*-3), 76.8 (*C*-6), 75.9 (*C*-5), 69.5 (*C*-2), 41.3 (*C*H_2_-CH=). **HRMS** (ESI+): calcd. for C_17_H_20_BrO_6_Si^+^ [M + H]+ 399.04378, found 399.0437.

## Figures and Tables

**Table 1:** Results of the multicomponent Hosomi–Sakurai reaction of bromopiperonal with TMS ethers **10–13** (see Scheme 4) ^1^.

Entry	TMS Ether	Product	Time	Yield ^2^	D.r. ^3^
1	**10**	**14a**	3 h	50%	57:43 ^4^
2	**11**	**16a**	2 h	89%	86:14 ^5^
3	**12**	**17a**	2 h	71%	87:13 ^5^
4	**13**	**15a**	3 h	53%	53:47 ^5^

^1^ All reactions were carried out at −78 °C in CH_2_Cl_2_ using 1 eq. of aldehyde (0.2 M), 1.4 equiv. of TMS ether, 1.5 equiv. of allyl-TMS, and 0.3 equiv. of TMS-OTf. ^2^ Isolated yield after chromatography. ^3^ The diastereomeric ratio was determined by ^1^H NMR on the crude product. Relative configuration not determined. ^4^ Diastereomers non-separated by chromatography. ^5^ Diastereomers separable by chromatography.

**Table 2:** Scope of multicomponent Hosomi–Sakurai reaction with TMS ether **11** (see Scheme 4) ^1^.

Entry	R	Product	Temp	Time	Conditions ^2^	Yield ^3^	D.r. ^4^
1	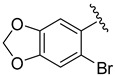	**16a**	−78 °C	2 h	A	89%	86:14 ^5^
2	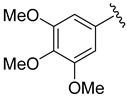	**16b**	−78 °C	1 h	A	96%	74:26 ^6^
3	4-ClC_6_H_4_	**16c**	−78 °C	1.5 h	A	76%	88:12 ^5^
4	3-MeOC_6_H_4_	**16d**	−78 °C	2 h	A	80%	90:10 ^5^
5	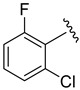	**16e**	−78 °C	4 h	A	37% ^7^	88:12 ^6^
6	4-O_2_NC_6_H_4_	**16f**	−78 °C	2 h	A	33% ^7^	92:8 ^5^
7	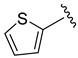	**16g**	−78 °C	1 h	A	95%	83:17 ^5^
8	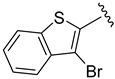	**16h**	−78 °C to −40 °C	2 h	A	71%	91:9 ^5^
9	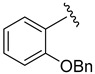	**16i**	−78 °C to −40 °C	2 h	A	45% ^7^	79:21 ^6^
10	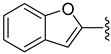	**16j**	−78 °C to −40 °C	2 h	A	<10% ^8^	-
11	*cy*Hex	**16k**	−78 °C to −40 °C	3 h	A	60%	66:34 ^6^
12	(H_3_C)_2_CH−CH_2_−	**16l**	−78 °C to −40 °C	3 h	A	38%	88:12 ^6^
13	(H_3_C)_2_CH−CH_2_−	**16l**	−78 °C to −40 °C	3 h	B	40% (84%)	88:12 ^6^
14	(H_3_C)_2_CH−CH_2_−	**16l**	−78 °C to −30 °C	3 h	C	59% (85%)	88:12 ^6^

^1^ All reactions were carried out in CH_2_Cl_2_ (0.2 M), using 1.5 equiv. of allyl-TMS. ^2^ Conditions A: 1.0 equiv. of aldehyde, 1.4 equiv. of TMS ether **11**, 0.3 equiv. of TMSOTf. Conditions B: 1.0 equiv. of **11**, 1.2 equiv. of aldehyde, 0.3 equiv. of TMSOTf. Conditions C: 1.0 equiv. of **11**, 1.0 + 0.5 equiv. of aldehyde, 0.4 + 0.4 equiv. of TMSOTf (the second aliquots added after 1 h). ^3^ Isolated yield after chromatography, based on the limiting agent, which was always the aldehyde, except for entries 13–14, where it was TMS ether **11**. For these two entries, yields based on not recovered starting material are shown in brackets. ^4^ The diastereomeric ratio was determined by ^1^H NMR on the crude product. For the relative configuration of the major diastereomer, see further discussion in the text. ^5^ Diastereomers separable by chromatography. ^6^ Diastereomers non-separable by chromatography. ^7^ Also, substantial amounts of bicomponent adducts were obtained. ^8^ Extensive decomposition of the starting aldehyde was observed.

## Data Availability

The original contributions presented in this study are included in the article/Supplementary Materials. Further inquiries can be directed to the corresponding author.

## Supplementary Materials

The following supporting information can be downloaded at: https://www.mdpi.com/article/10.3390/molecules31122155/s1, Discussion on the relative configuration of the main diastereomers; Table S1: Characteristic Chemical shifts (^1^H NMR) of the two diastereomers; Figure S1: Figure S2: Preferred conformation for the (*S*) simplified product 16a; Figure S3: Preferred conformation for the (*R*) simplified product **16a**. Copy of all ^1^H and ^13^C NMR spectra.

## Abbreviations

The following abbreviations are used in this manuscript:

Bn	Benzyl
DMAP	4-Dimethylaminopyridine
DMF	*N*,*N*-dimethylformamide
MCR	Multicomponent Reaction
MHS	Multicomponent Hosomi–Sakurai
PE	Petroleum ether 40–60
TBAF	Tetrabutylammonium fluoride
TBDPS	*tert*-Butildiphenylsilyl
Tf	Trifluoromethanesulfonyl
THF	Tetrahydrofuran
TMS	Trimethylsilyl
