# Supplementary material for: Multicomponent Hosomi–Sakurai Reaction on Isosorbide Derivatives
Source: Molecules. 2026 Jun 18;31(12):2155. doi: 10.3390/molecules31122155 (PMC13305661; doi:10.3390/molecules31122155)
Supplement: Supplementary file 1 [file molecules-31-02155-s001.zip › molecules-4359281-supplementary.pdf]

Multicomponent Hosomi-Sakurai reaction on Isosorbide Derivatives

Luca Banfi,* Lucia Garcia de la Parte, Chiara Lambruschini, Lisa Moni, Daniel Rufo Perez, and Renata Riva.

SUPPORTING INFORMATION

Summary:

Discussion on relative configuration of the main diastereomers 2
Copies of ^{1}H and ^{13}C NMR spectra 7

Discussion on relative configuration of the main diastereomers

First of all, we noticed some ^{1}H NMR trends that were constant along the whole series, at least for the products **16a**-**16j**, derived from aromatic aldehydes. As shown in the following Table S1, we noticed regular significant differences in chemical shift for *H*-3a and *H*-2. *H*-3a is shifted upfield in the major isomer. On the contrary, both *H*-2, but especially the one falling at higher δ, are shifted upfield for the minor isomer. The two *H*-2 may be differentiated by the coupling constant with *H*-3. In both the major and minor isomers, the upfield (lower δ) *H*-2 has *J*≈8.4 Hz, whereas the downfield (higher δ) *H*-2 has *J*≈ 7.0 Hz. The downfield *H*-2 (lower *J*) is the one that experiences the highest difference. It is probably the one placed on the opposite side of the oxygen bound at *C*-3 (down, coloured in red in the following Figure S1).

Table S1. Characteristic Chemical shifts (^{1}H NMR) of the two diastereomers

Compound	Major	Minor	Diff.	Major	Minor	Diff.
	***H*-3a**	***H*-3a**		***H*-2**	***H*-2**	
**16a**	4.45	4.73	0.28	3.86, 3.49	3.58, 3.39	0.28, 0.10
**16b**	4.32[a]	4.68	0.36	3.88,[a] 3.47	3.53, 3.40	0.35, 0.07
**16c**	4.35	4.67	0.32	3.82, 3.47	3.52, 3.39	0.30, 0.08
**16d**	4.36	4.68	0.32	3.86, 3.47	3.52, 3.40	0.34, 0.07
**16e**	4.34	4.76	0.32	3.89,[a] 3.50	3.57, 3.42	0.32, 0.08
**16f**	4.42	4.76	0.34	3.83, 3.53	3.56,[b] 3.45[b]	0.37, 0.08
**16g**	4.34	4.68	0.34	3.84, 3.44	3.50, 3.35	0.34, 0.09
**16h**	4.51	4.81	0.30	3.97, 3.52	3.62, 3.48	0.35, 0.04
**16i**	4.44	4.66	0.22	3.82,[a] 3.47	3.54, 3.39	0.28, 0.08
**16k**	4.62	4.61	-0.01	3.79, 3.37	3.79, 3.40	0, -0.03
**16l**	4.63	4.63	0	3.82, 3.39	3.82, 3.39	0, 0

[a] Determined by HSQC. b Determined on the spectrum of the crude product

The other protons of the bicyclic system show little differences in δ among the two isomers.
Interestingly, **this trend is only valid for the products derived from aromatic aldehydes**, and it is not present in the two products derived from aliphatic aldehydes (**16k** and **16j**). Thus, this difference is surely due to anisotropic effects given by the aromatic ring.
If we compare the chemical shifts in the aromatic products with those in the aliphatic ones, it is clear that ***H*-3a is shielded in the major diastereomer, whereas one of the two *H*-2 is shielded in the minor isomer**. Thus, they should fall in the shielding cone of the aromatic ring.

Figure S1

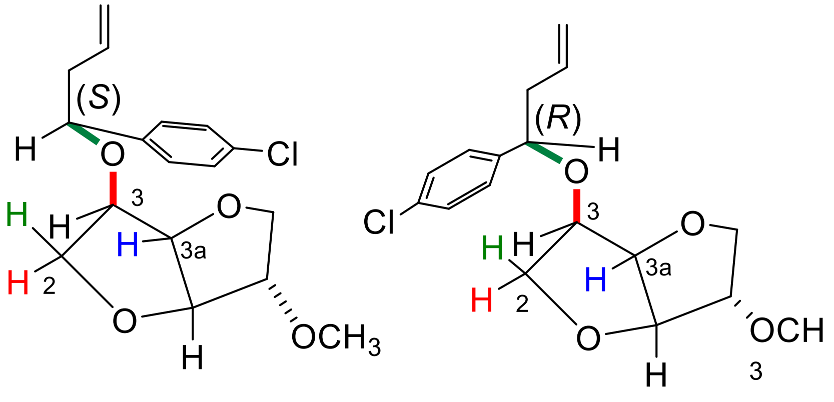

simplified analogue of (***S***) **16c**   simplified analogue of (***R***) **16c**

In order to try to understand this behaviour, we used Chem3D™ with the AM1 algorithm (MOPAC) to search for the likely more stable conformations of the products. For the sake of simplicity (in order to avoid too many local minima) and for a clearer visualization, we carried out minimization on a simplified analog of **16c**, by substituting the TBDPS group with a methyl group, since it is reasonable that this would not influence the conformational equilibrium, being this group very far away form the new stereogenic center.

Fortunately, the bicyclic scaffold is pretty rigid and thus the only two important rotatable bonds are the ones coloured in red and green in Figure S1.
As far as it regards the red bond, the preferred conformations, without any doubt, are those that place the bulky substituent outside the bicyclic ring, in order to minimize steric strain. Less obvious are the preferred conformations deriving from rotation of the green bond. Considering only the three staggered conformations, one of the three substituents at the new stereogenic centre should be *anti* (180° dihedral angle) with C3-O bond.
The ones placing the hydrogen in this position were definitely less favoured by our minimizations, as expected, being "doubly gauche" conformations. Moreover, the presence of a nOe between the "blue" hydrogen and the "purple" one are against this possibility.
Also conformations placing the aryl group upside are less favoured by our minimization, but, most of all, they are in contrast with the absence of any nOe between the protons of the allyl group and the various protons of the bicyclic system.
Therefore, the best ones turned out to be, for both isomers, those placing the allyl group at 180° dihedral angle with the red bond and upside. These conformations, shown in Figures S1, are in agreement with the absence of nOe between the allyl group hydrogens and any of the bicyclic system, and with nOe experiments showing a close distance between blue and purple hydrogens in both isomers (the distance is similar).
If these are the preferred conformations, the shielding anisotropic effect of the aryl group would act differently in the two isomers. In the (*S*) isomer this effect is expected to move upfield *H*-3a, whereas in the (*R*) isomer this effect will act on both *H*-2 hydrogens, but especially the lower one (drawn in red). This may be better seen by the following images of the minimized conformations (Figures S2 and S3). In this hypothesis, the major diastereoisomer would be the (*S*) one.
Arguments based on the shielding effect of aromatic rings have been often used in assigning relative configurations, for example in the well known Mosher's method.
It should be stressed that this is just an hypothesis, based on the accordance between experimental spectroscopic data and conformational minimization with MOPAC, and thus we can not state with 100% certainty that this assignment is correct.

Figure S2. **Preferred conformation for the (*S*) simplified product 16a.** These are simply different views of the same conformation. grey: carbon; yellow: hydrogen; red: oxygen; green: chlorine

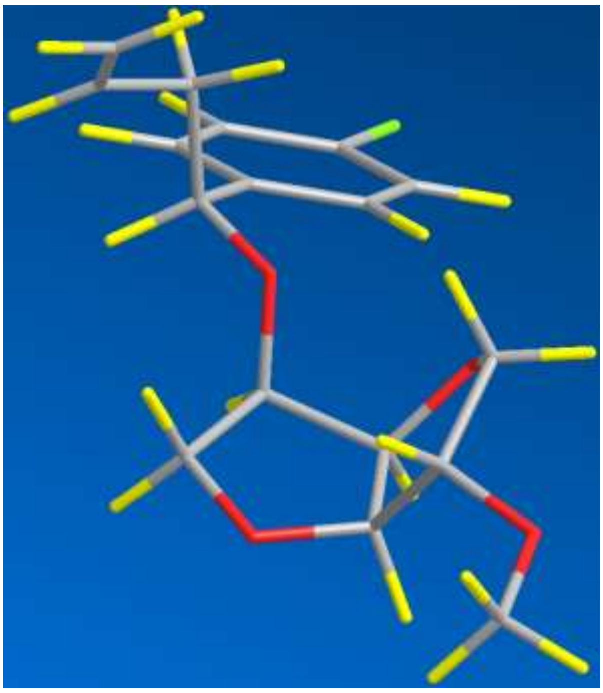

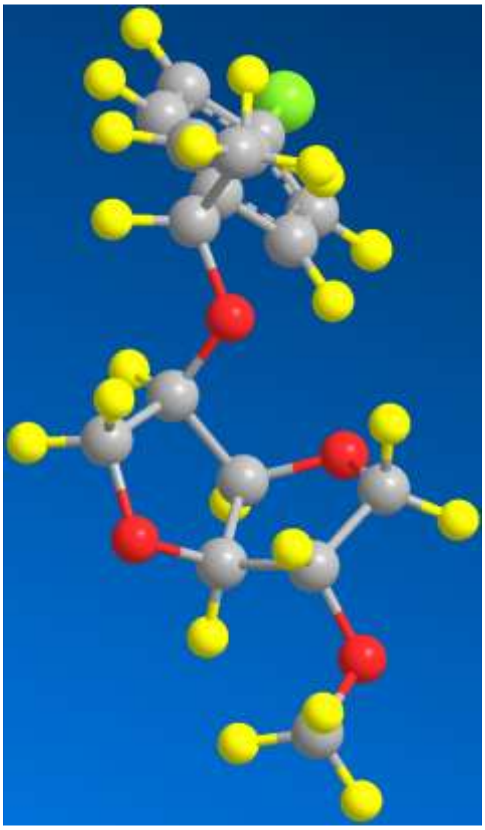

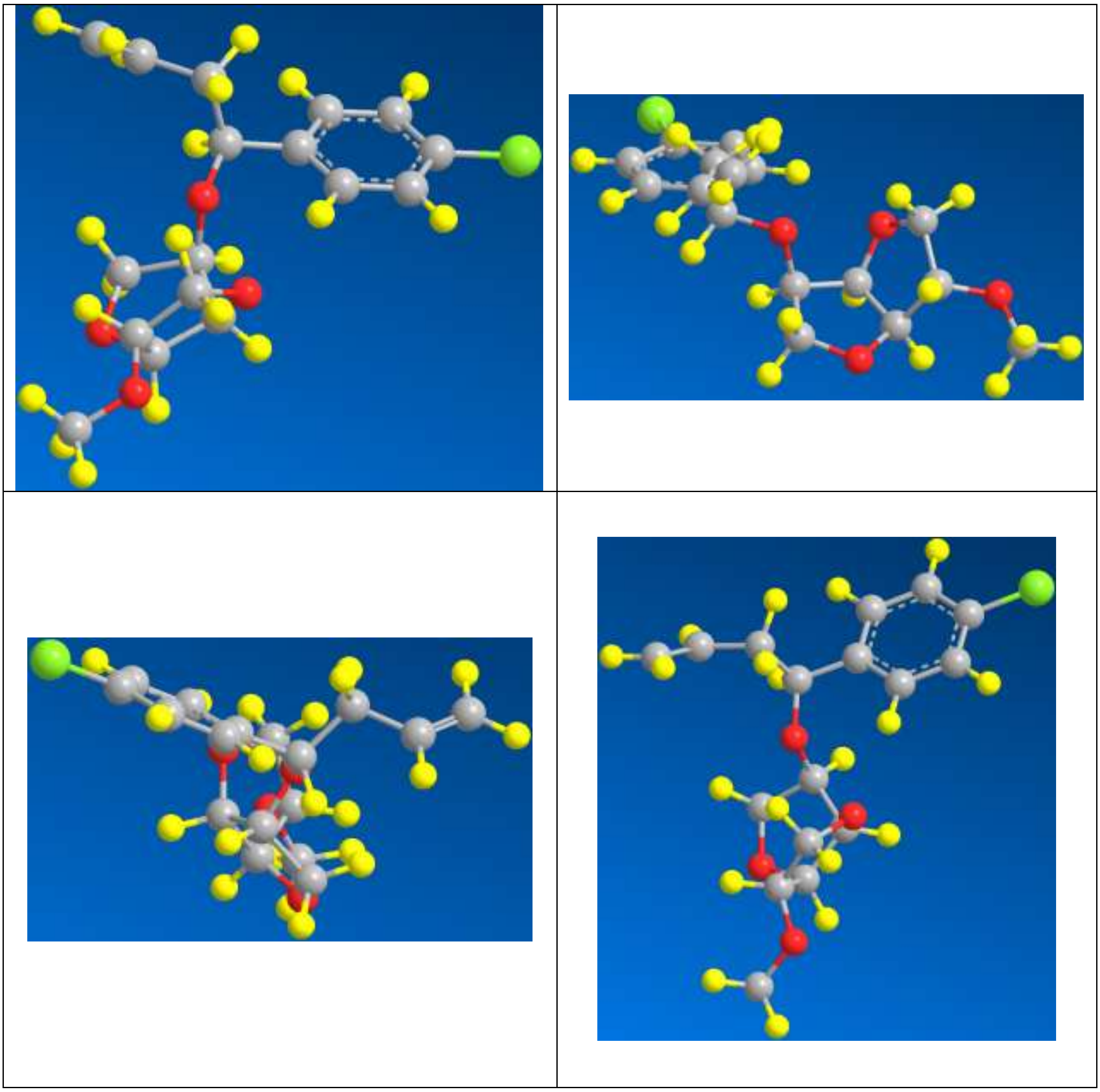

**Figure S3**. **Preferred conformation for the (*R*) simplified product 16a.** These are simply different views of the same conformation. grey: carbon; yellow: hydrogen; red: oxygen; green: chlorine	
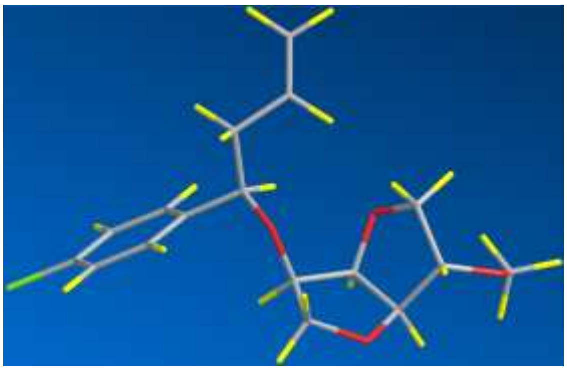	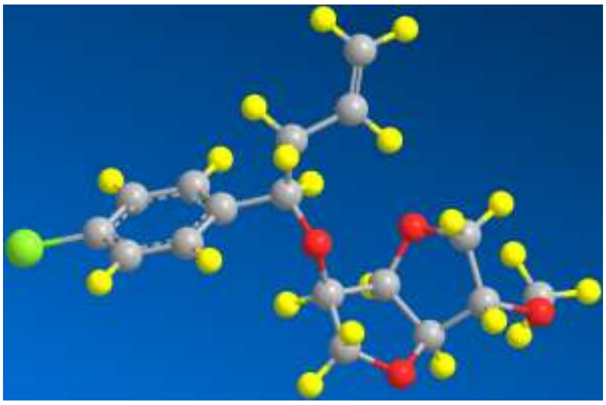
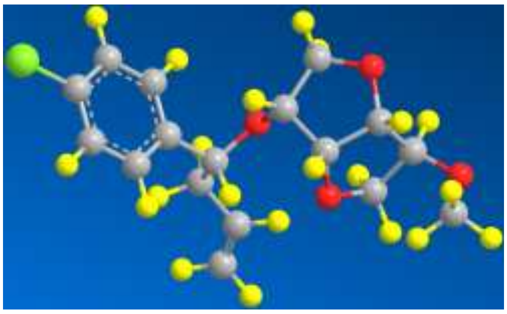	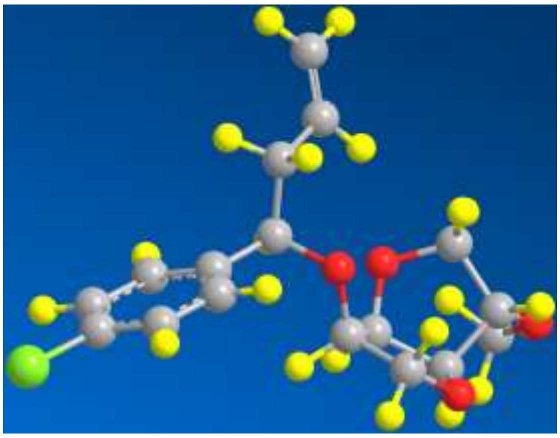

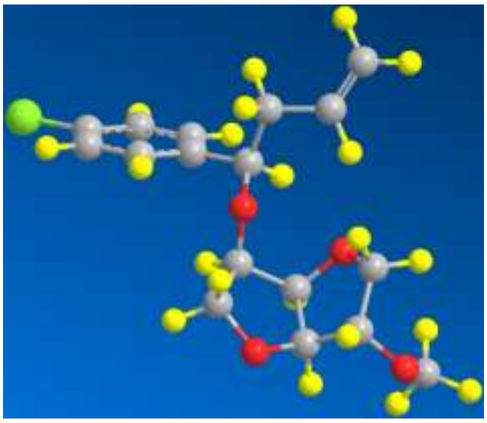	

Copies of ^{1}H and ^{13}C NMR spectra

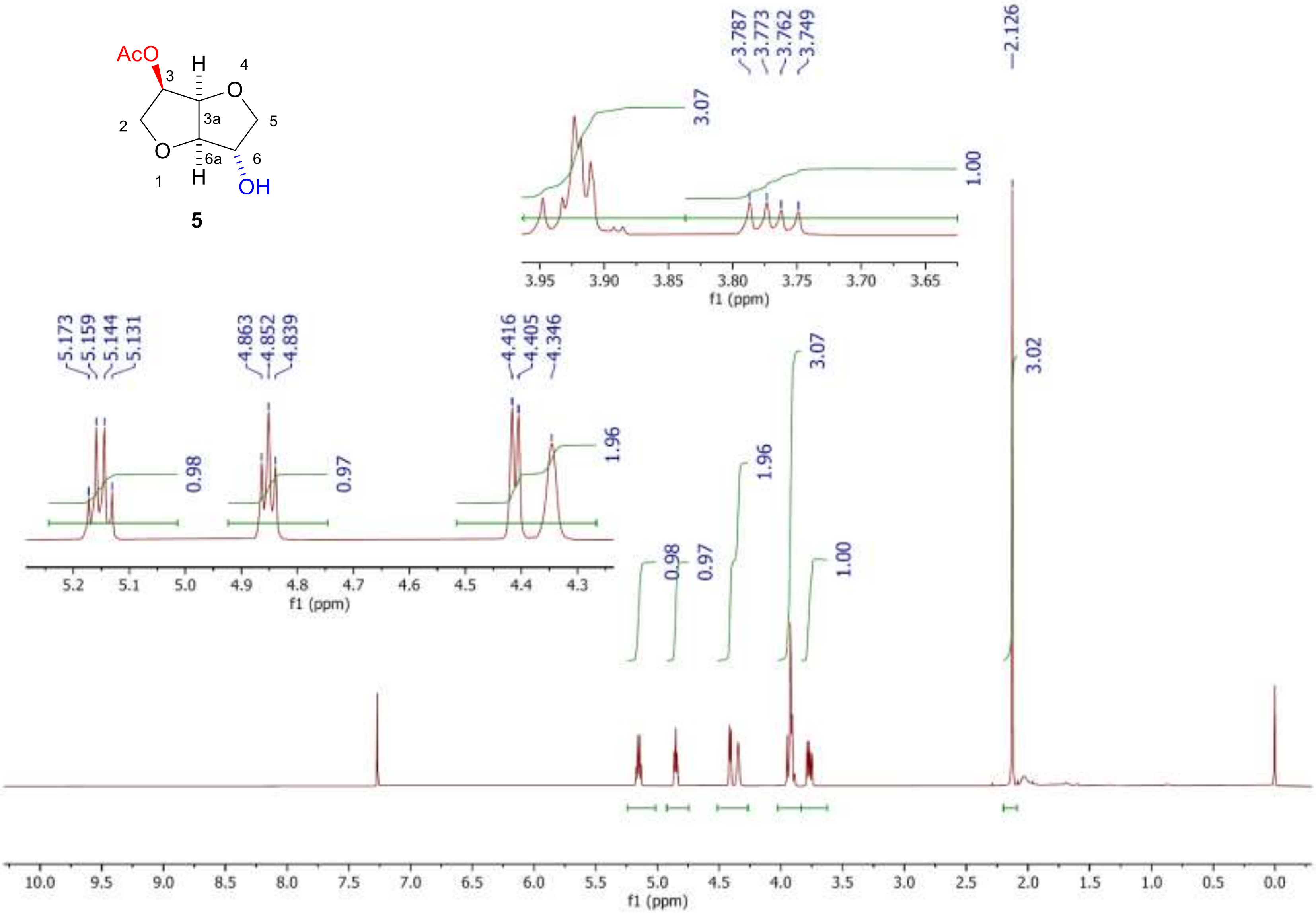

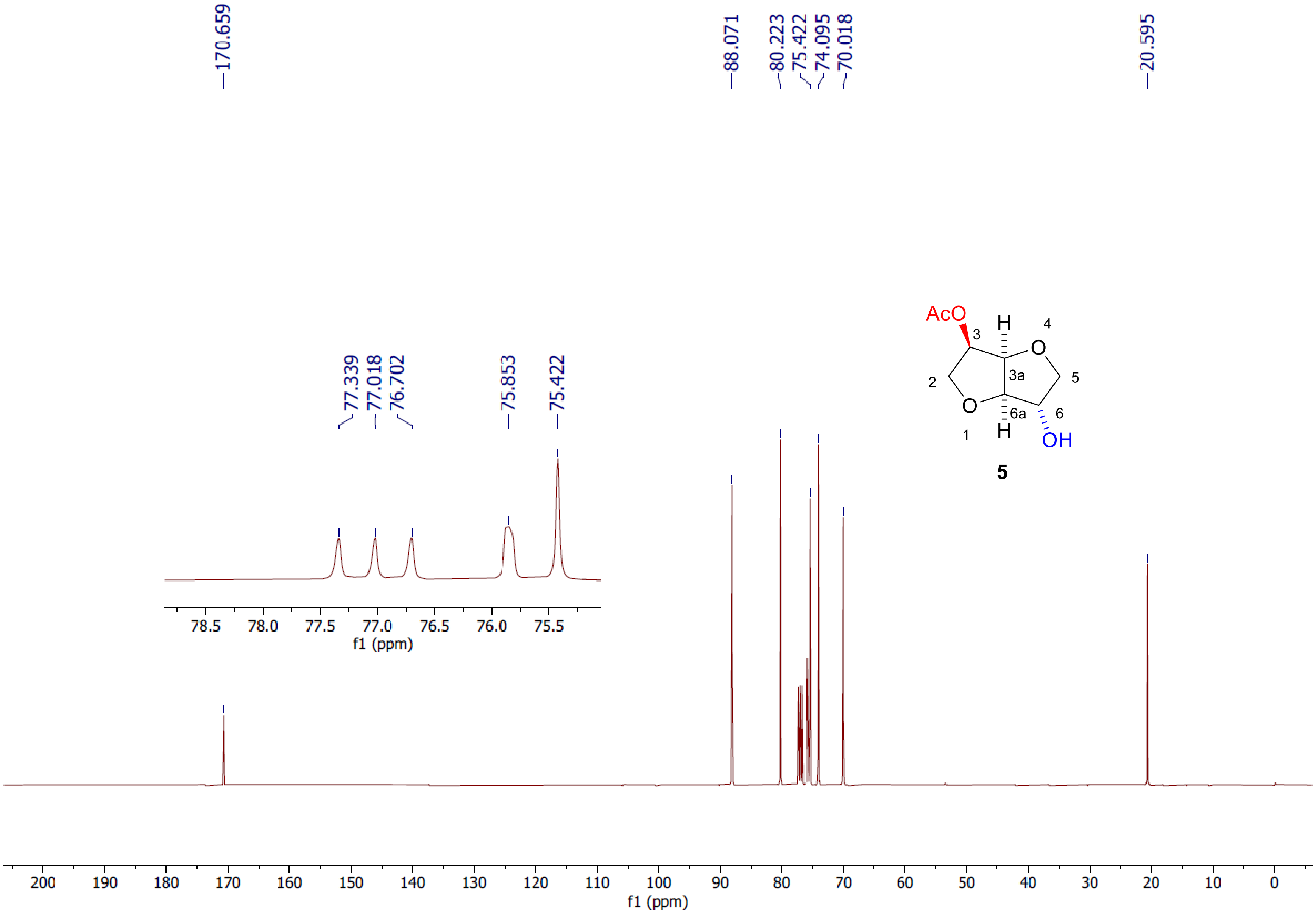

170.659
88.071
80.223
75.422
74.095
70.018
20.595
77.339
77.018
76.702
75.853
75.422
AcO
H
4
3
O
3a
5
2
O
6a
6
1
H
OH
5
f1 (ppm)
f1 (ppm)

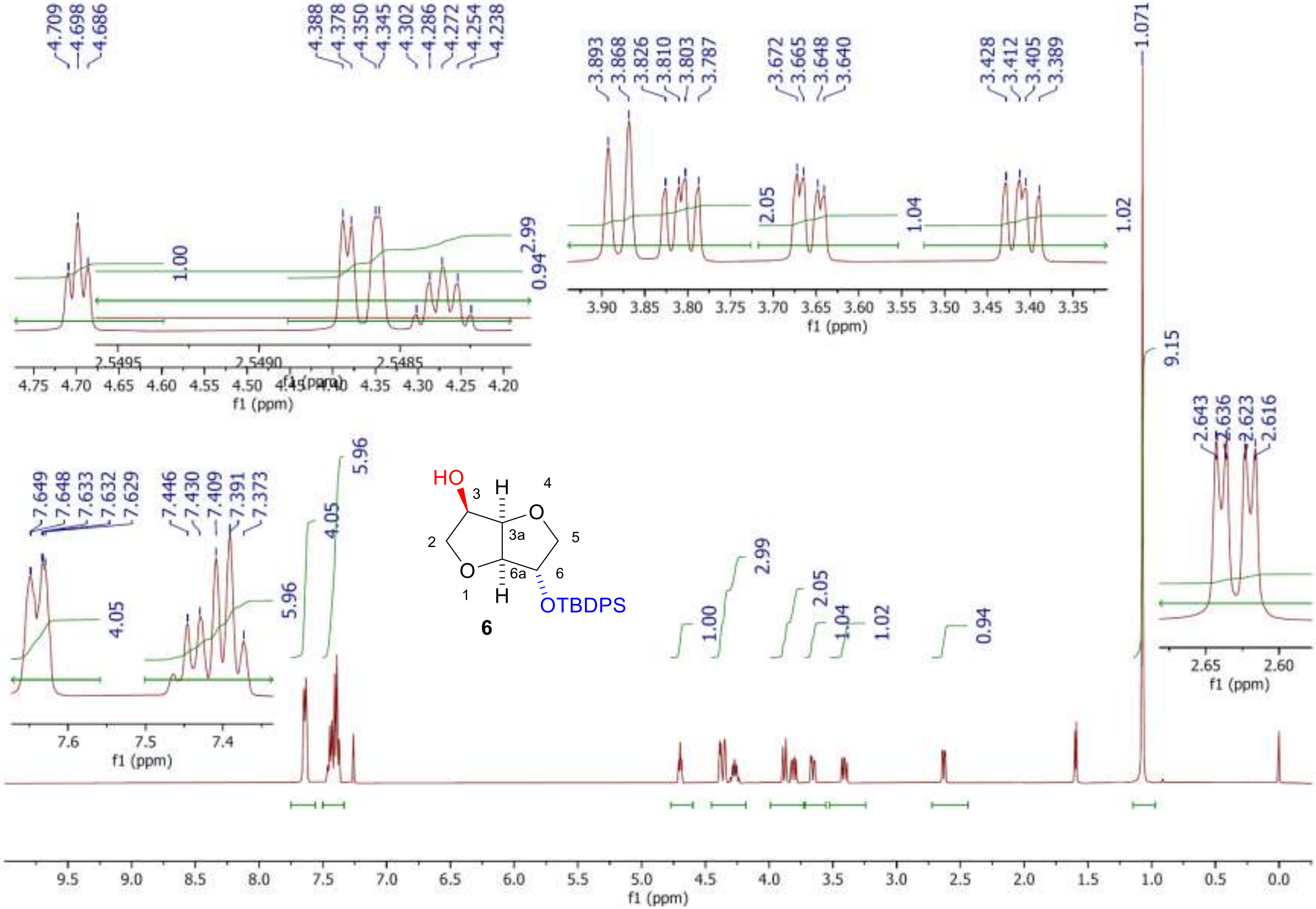
4.709
4.698
4.686
4.388
4.378
4.350
4.345
4.302
4.286
4.272
4.254
4.238
1.00
0.94
2.99
f1 (ppm)
3.893
3.868
3.826
3.810
3.803
3.787
3.672
3.665
3.648
3.640
3.428
3.412
3.405
3.389
2.05
1.04
1.02
f1 (ppm)
1.071
9.15
2.643
2.636
2.623
2.616
f1 (ppm)
7.649
7.648
7.633
7.632
7.629
7.446
7.430
7.409
7.391
7.373
4.05
5.96
f1 (ppm)
HO
H
OTBDPS
6
f1 (ppm)

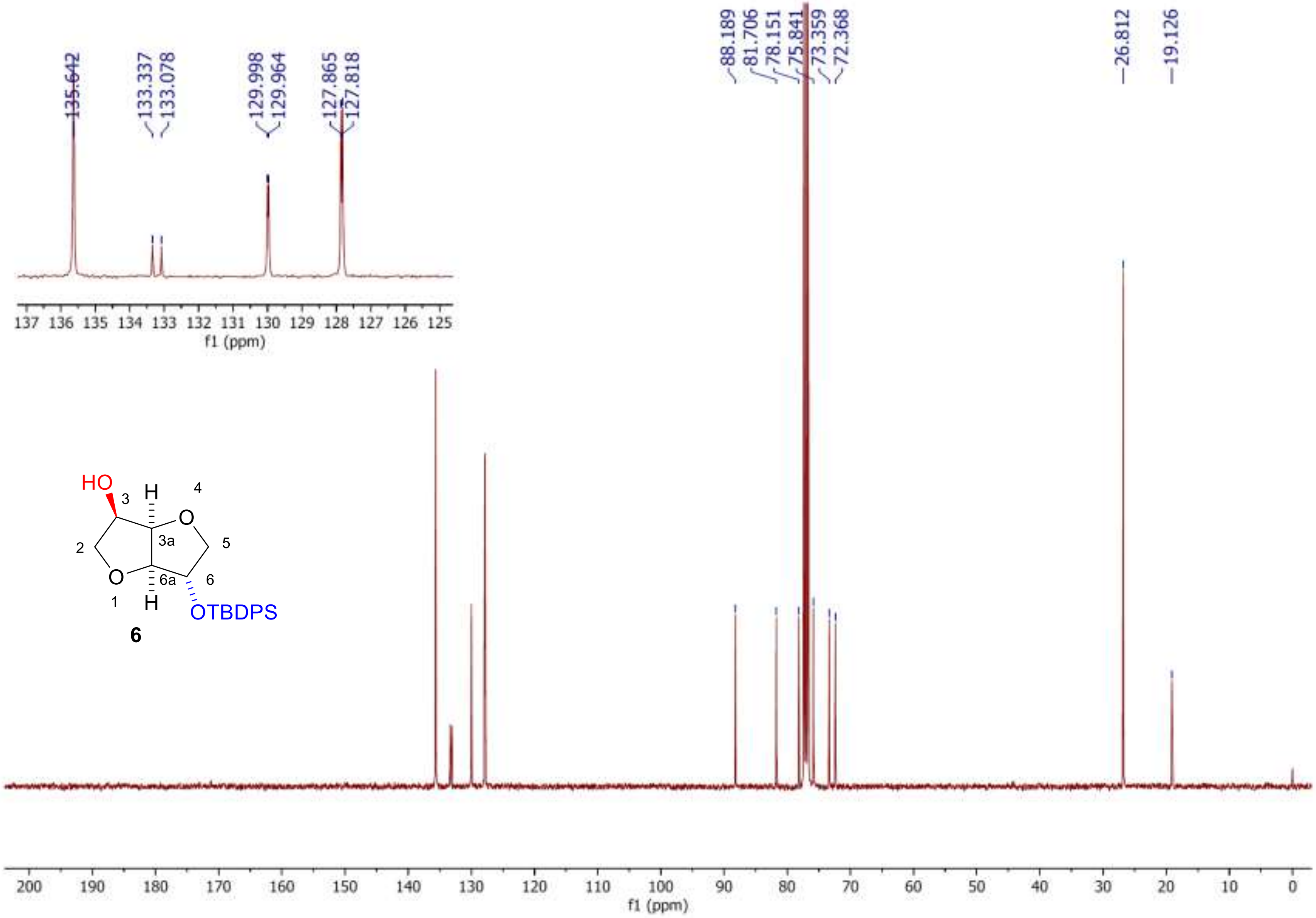
135.642
133.337
133.078
129.998
129.964
127.865
127.818
137 136 135 134 133 132 131 130 129 128 127 126 125
f1 (ppm)
88.189
81.706
78.151
75.841
73.359
72.368
26.812
19.126
HO
3
H
4
O
3a
2
5
O
6a
6
1
H
OTBDPS
6
200 190 180 170 160 150 140 130 120 110 100 90 80 70 60 50 40 30 20 10 0
f1 (ppm)

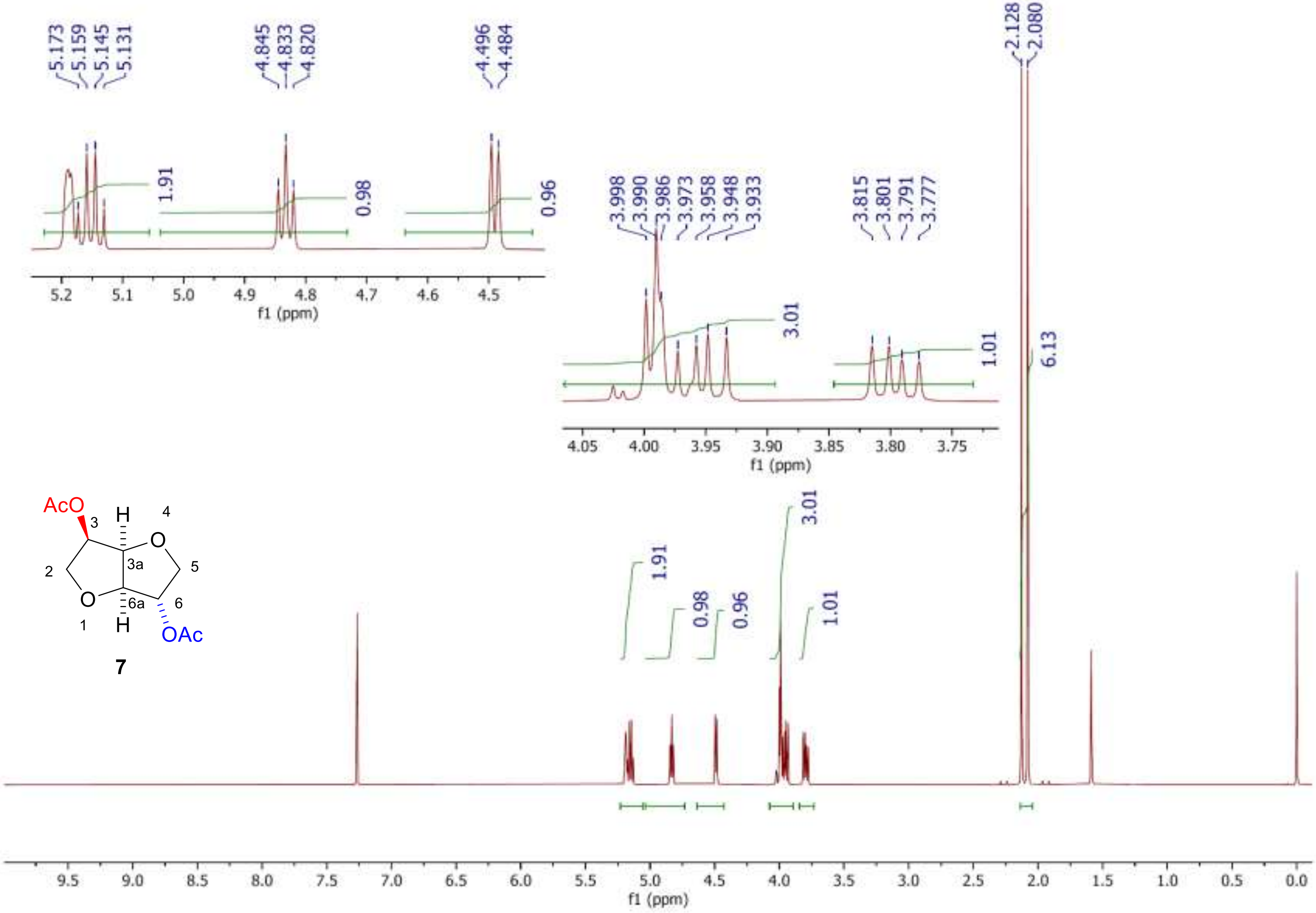
AcO
OAc
H
H
O
O
3
3a
4
5
6
6a
2
1
7
5.173
5.159
5.145
5.131
4.845
4.833
4.820
4.496
4.484
1.91
0.98
0.96
3.998
3.990
3.986
3.973
3.958
3.948
3.933
3.815
3.801
3.791
3.777
3.01
1.01
2.128
2.080
6.13
f1 (ppm)

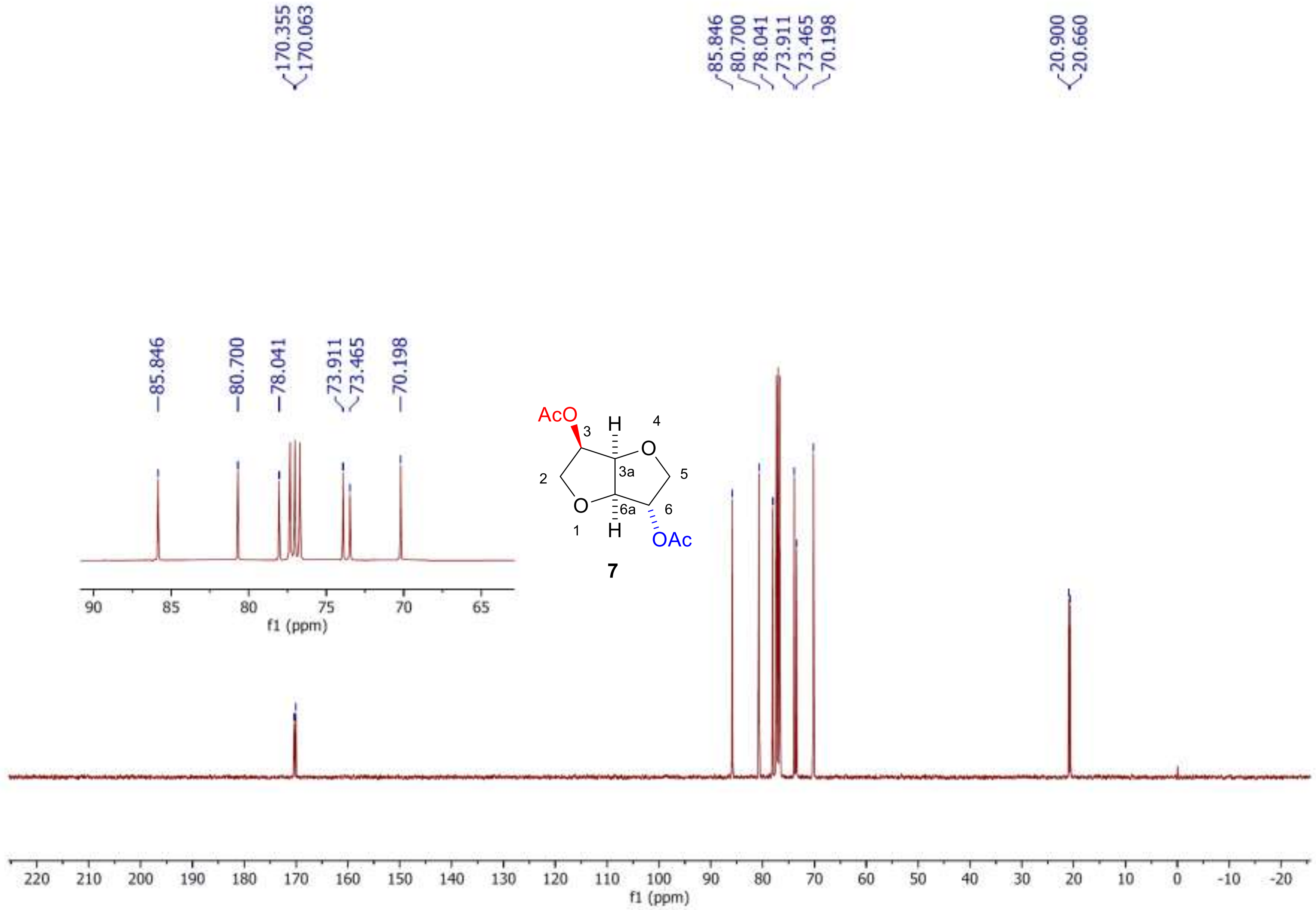
170.355
170.063
85.846
80.700
78.041
73.911
73.465
70.198
20.900
20.660
AcO
H
O
3
4
3a
5
2
6a
6
O
H
1
OAc
7
f1 (ppm)
f1 (ppm)

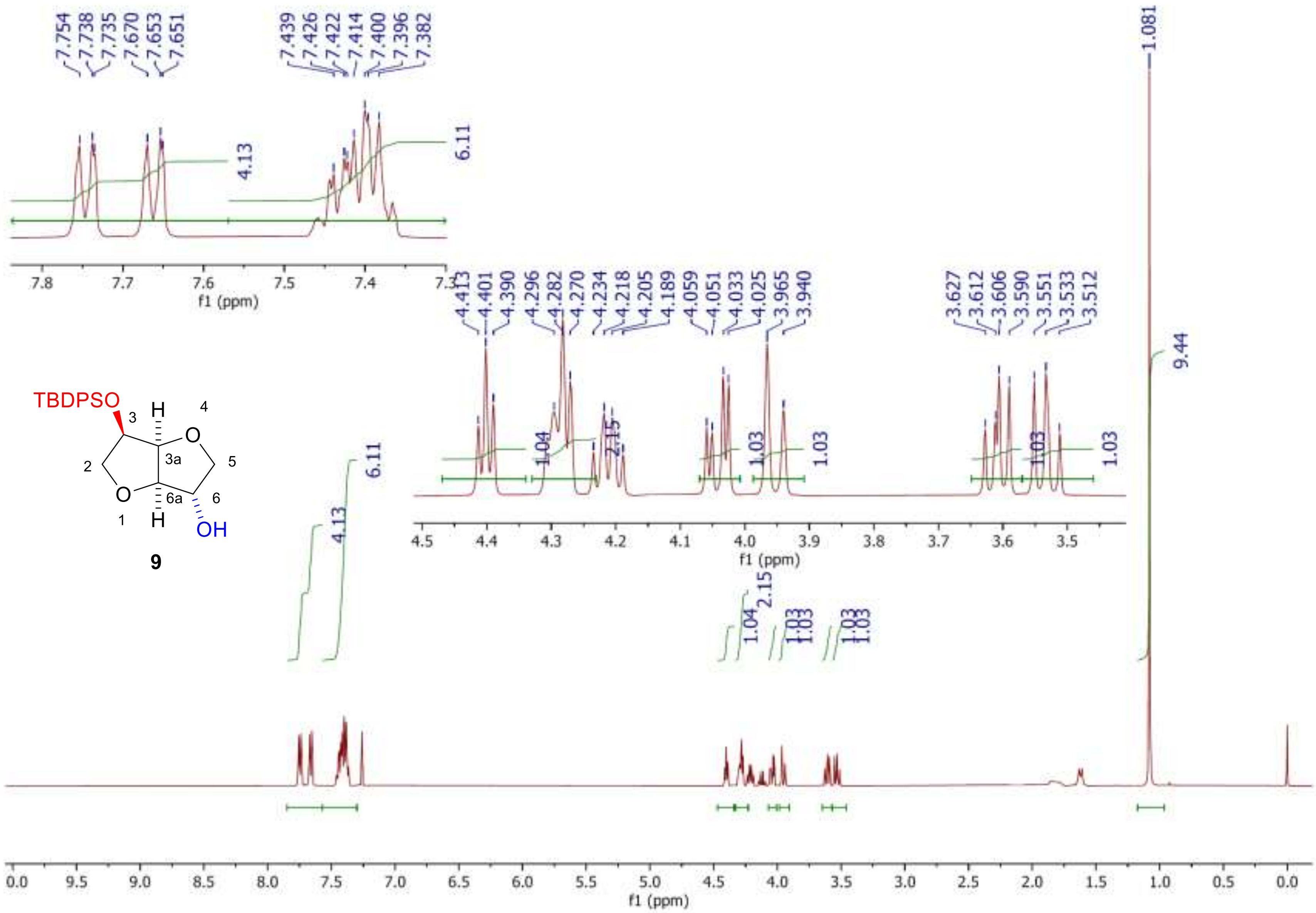
TBDPSO
OH
9
f1 (ppm)
7.754
7.738
7.735
7.670
7.653
7.651
7.439
7.426
7.422
7.414
7.400
7.396
7.382
1.081
4.413
4.401
4.390
4.296
4.282
4.270
4.234
4.218
4.205
4.189
4.059
4.051
4.033
4.025
3.965
3.940
3.627
3.612
3.606
3.590
3.551
3.533
3.512
4.13
6.11
1.04
2.15
1.03
9.44

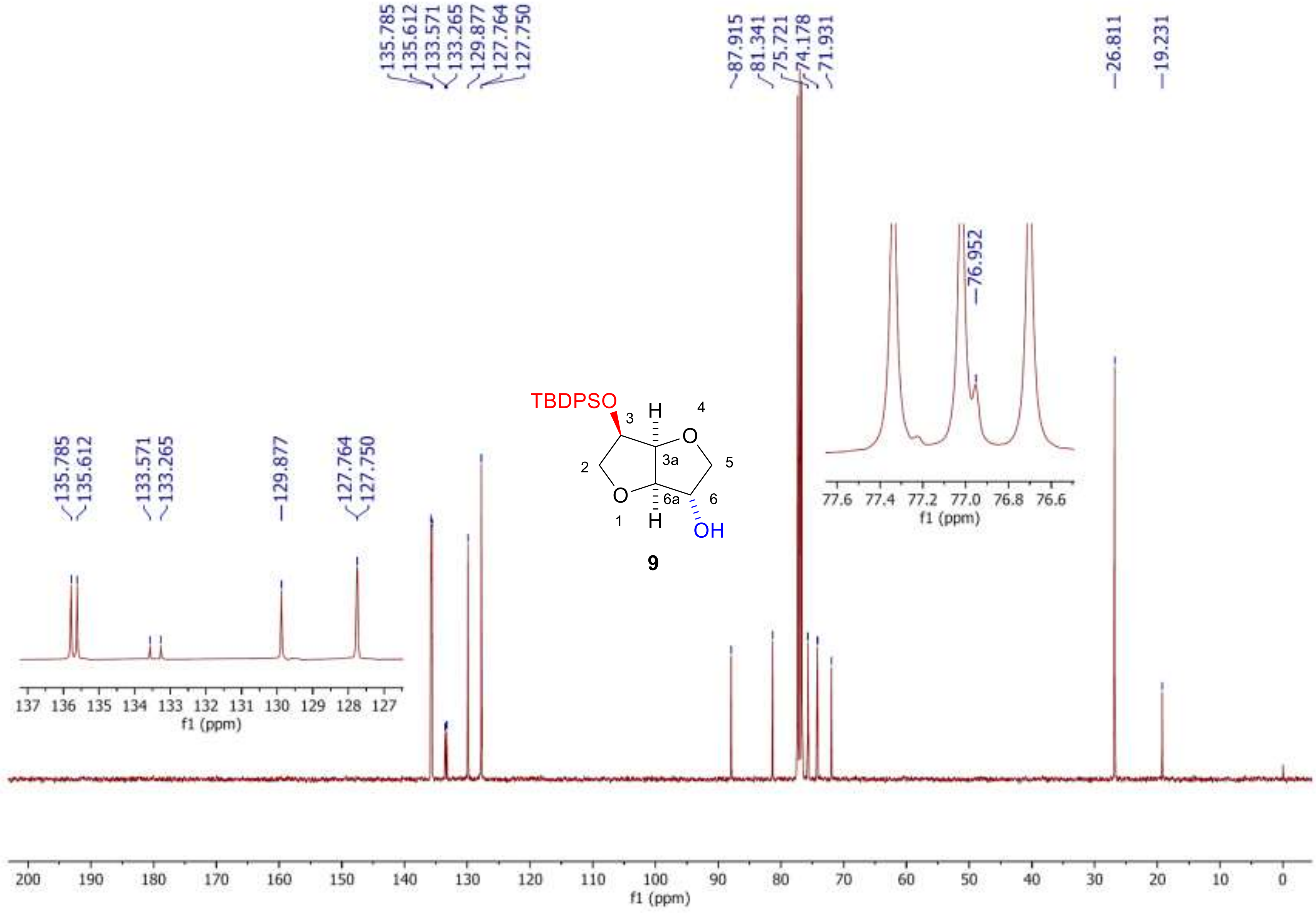
135.785
135.612
133.571
133.265
129.877
127.764
127.750
87.915
81.341
75.721
74.178
71.931
26.811
19.231
76.952
TBDPSO
H
O
3
4
2
3a
5
O
6a
6
1
H
OH
9
137 136 135 134 133 132 131 130 129 128 127
f1 (ppm)
77.6 77.4 77.2 77.0 76.8 76.6
f1 (ppm)
200 190 180 170 160 150 140 130 120 110 100 90 80 70 60 50 40 30 20 10 0
f1 (ppm)

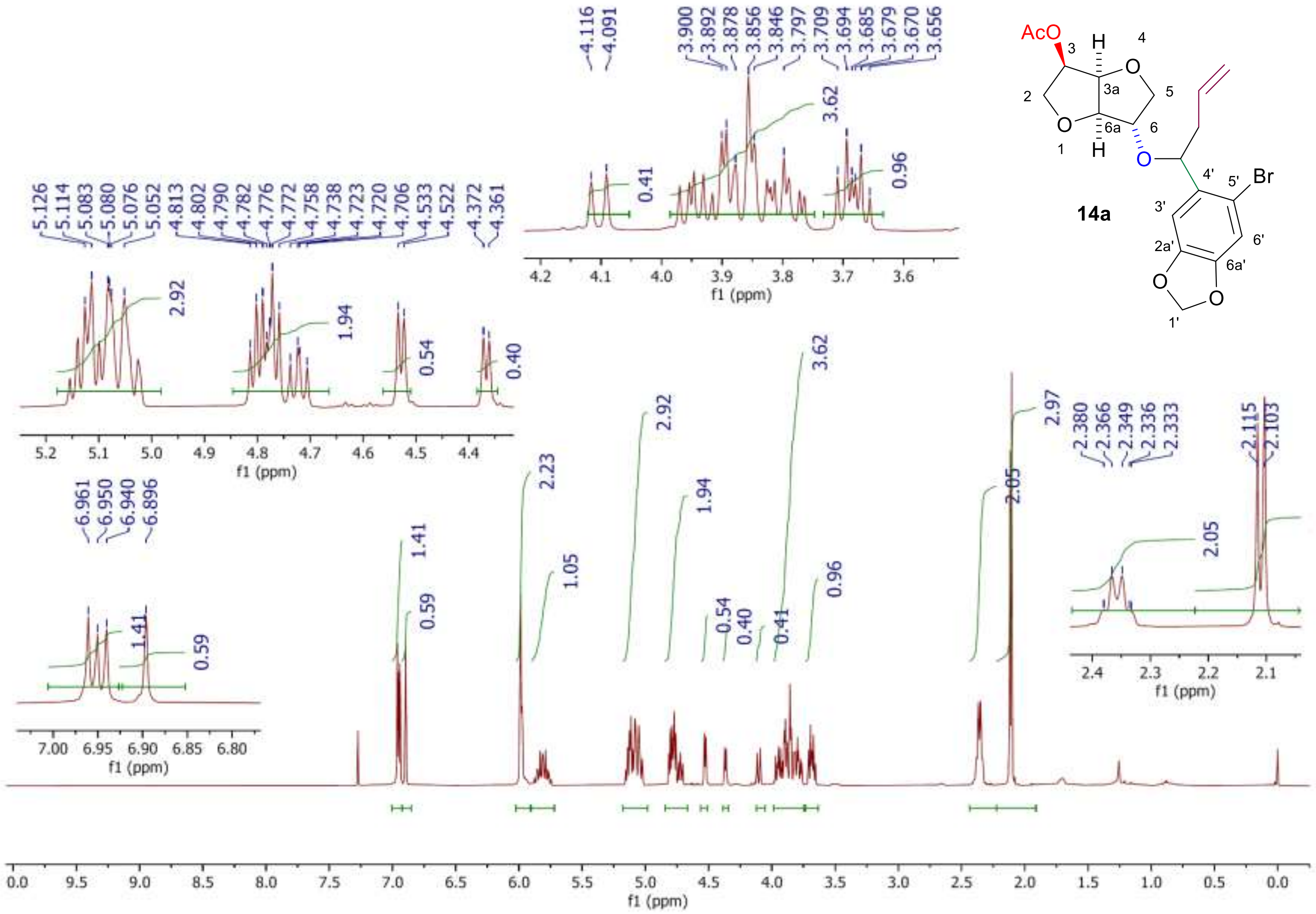

AcO
3
H
4
O
2
3a
5
O
6a
6
1
H
O
4'
5'
Br
3'
6'
2a'
6a'
O
O
1'

14a

5.126
5.114
5.083
5.080
5.076
5.052
4.813
4.802
4.790
4.782
4.776
4.772
4.758
4.738
4.723
4.720
4.706
4.533
4.522
4.372
4.361
2.92
1.94
0.54
0.40
f1 (ppm)
4.116
4.091
3.900
3.892
3.878
3.856
3.846
3.797
3.709
3.694
3.685
3.679
3.670
3.656
0.41
3.62
0.96
6.961
6.950
6.940
6.896
1.41
0.59
2.380
2.366
2.349
2.336
2.333
2.115
2.103
2.05
2.97
2.23
1.05
f1 (ppm)

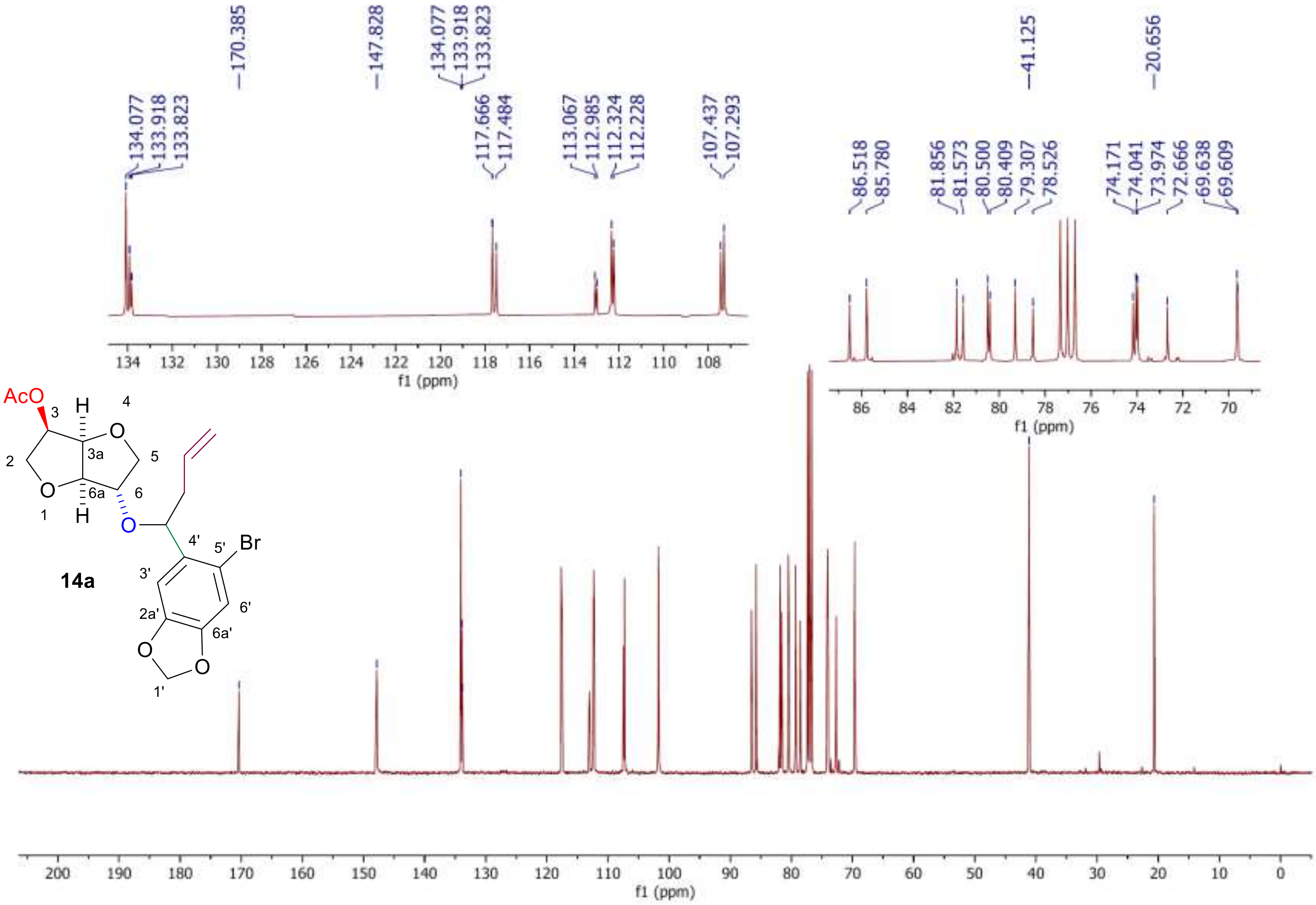
170.385
147.828
134.077
133.918
133.823
41.125
20.656
117.666
117.484
113.067
112.985
112.324
112.228
107.437
107.293
86.518
85.780
81.856
81.573
80.500
80.409
79.307
78.526
74.171
74.041
73.974
72.666
69.638
69.609
f1 (ppm)
AcO
H
H
O
O
O
O
O
Br
1
2
3
3a
4
5
6
6a
1'
2a'
3'
4'
5'
6'
6a'

14a

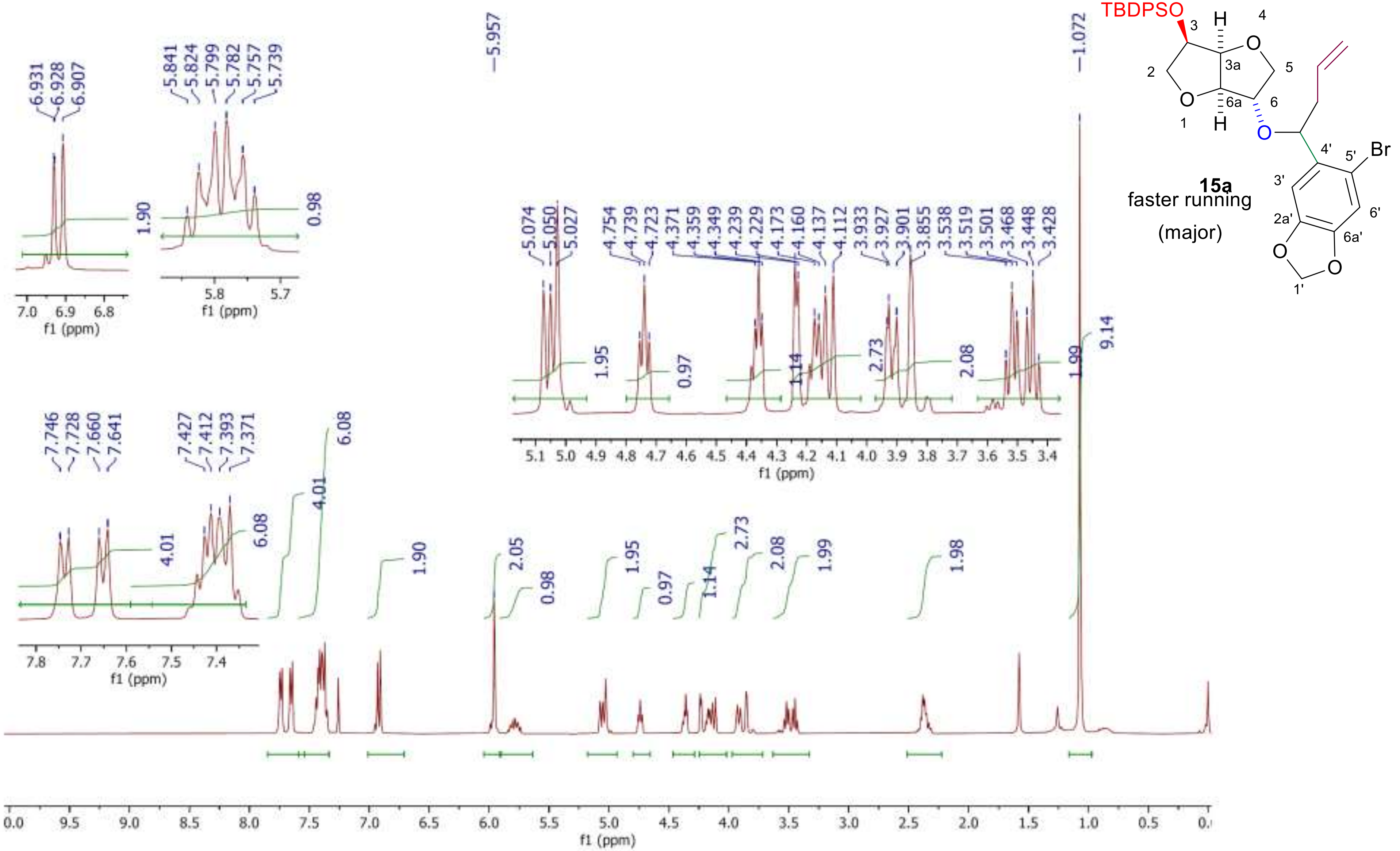
TBDPSO
15a
faster running
(major)
Br
f1 (ppm)

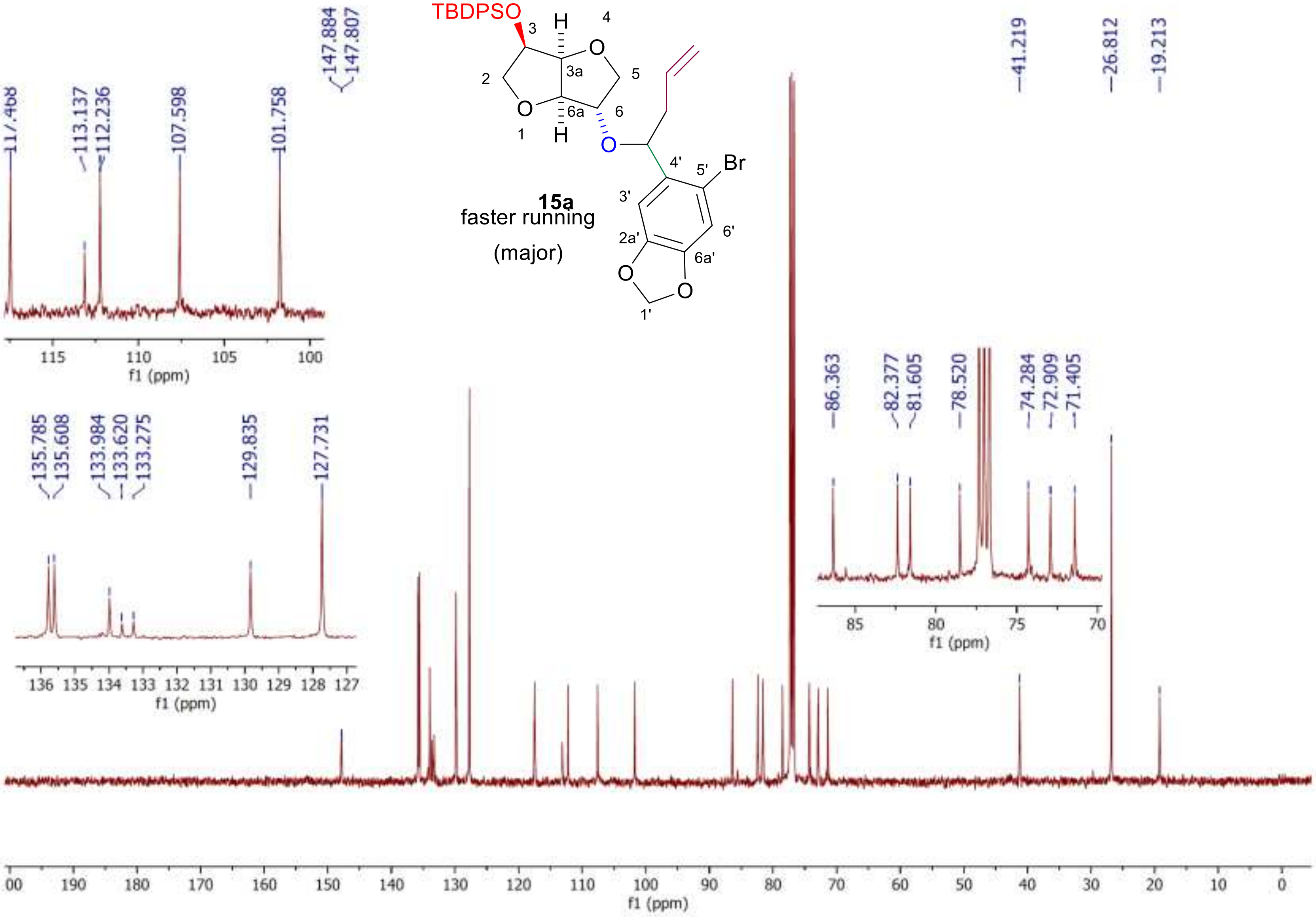
TBDPSO
15a
faster running
(major)
Br
117.468
113.137
112.236
107.598
101.758
147.884
147.807
41.219
26.812
19.213
135.785
135.608
133.984
133.620
133.275
129.835
127.731
86.363
82.377
81.605
78.520
74.284
72.909
71.405
f1 (ppm)

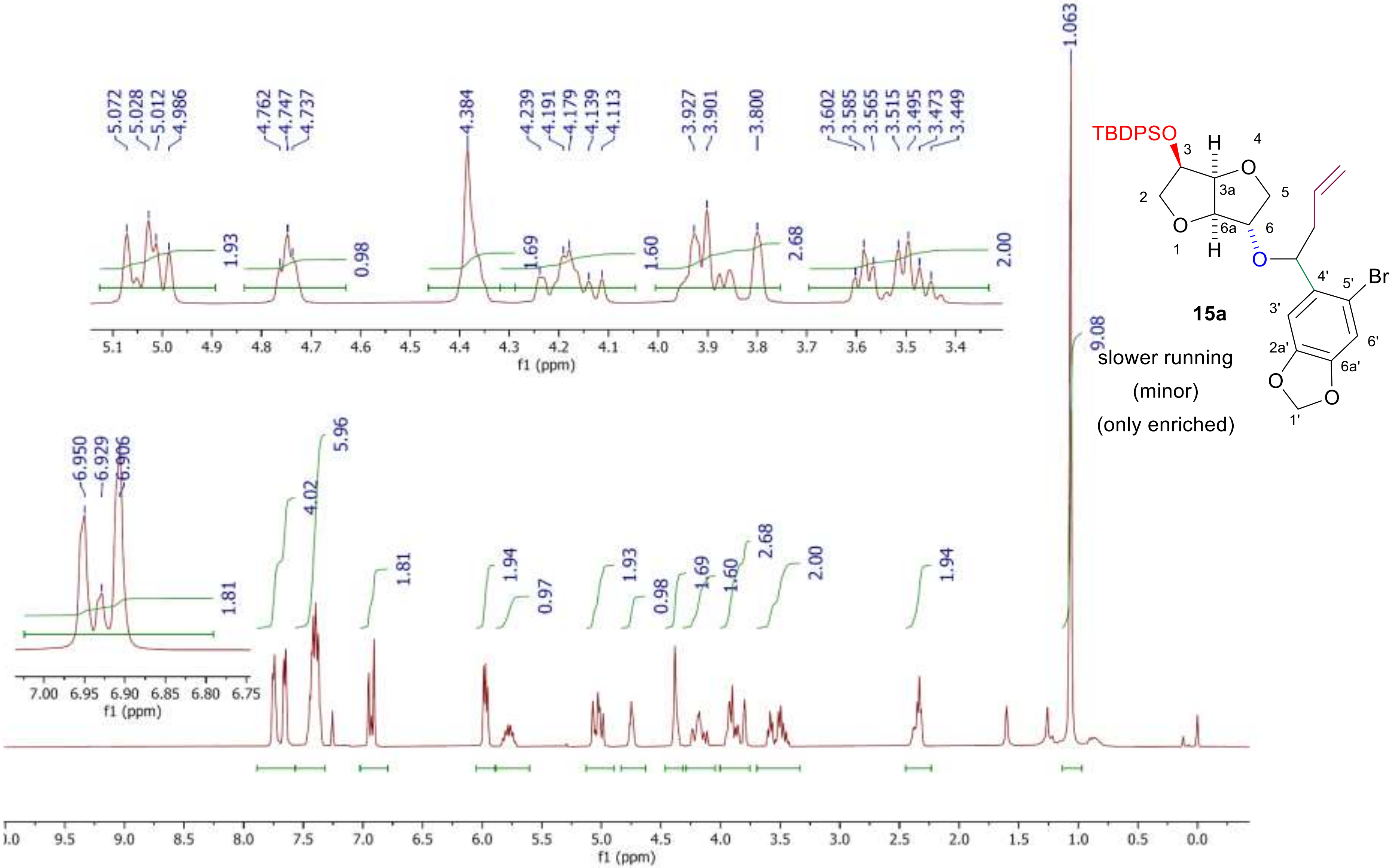
TBDPSO
Br
15a
slower running
(minor)
(only enriched)
f1 (ppm)
f1 (ppm)

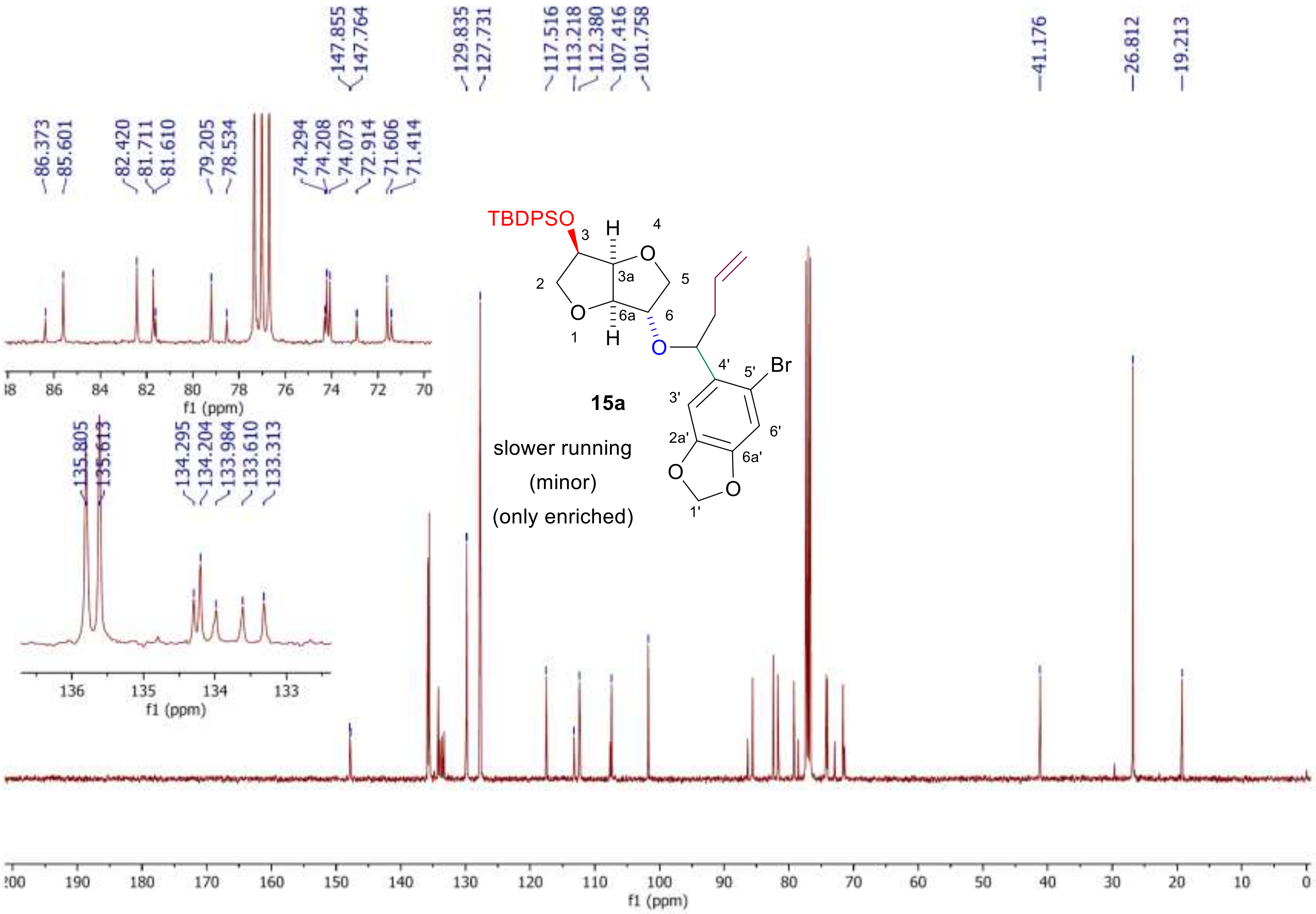
147.855
147.764
129.835
127.731
117.516
113.218
112.380
107.416
101.758
41.176
26.812
19.213
86.373
85.601
82.420
81.711
81.610
79.205
78.534
74.294
74.208
74.073
72.914
71.606
71.414
135.805
135.613
134.295
134.204
133.984
133.610
133.313
TBDPSO
Br
15a
slower running
(minor)
(only enriched)
f1 (ppm)

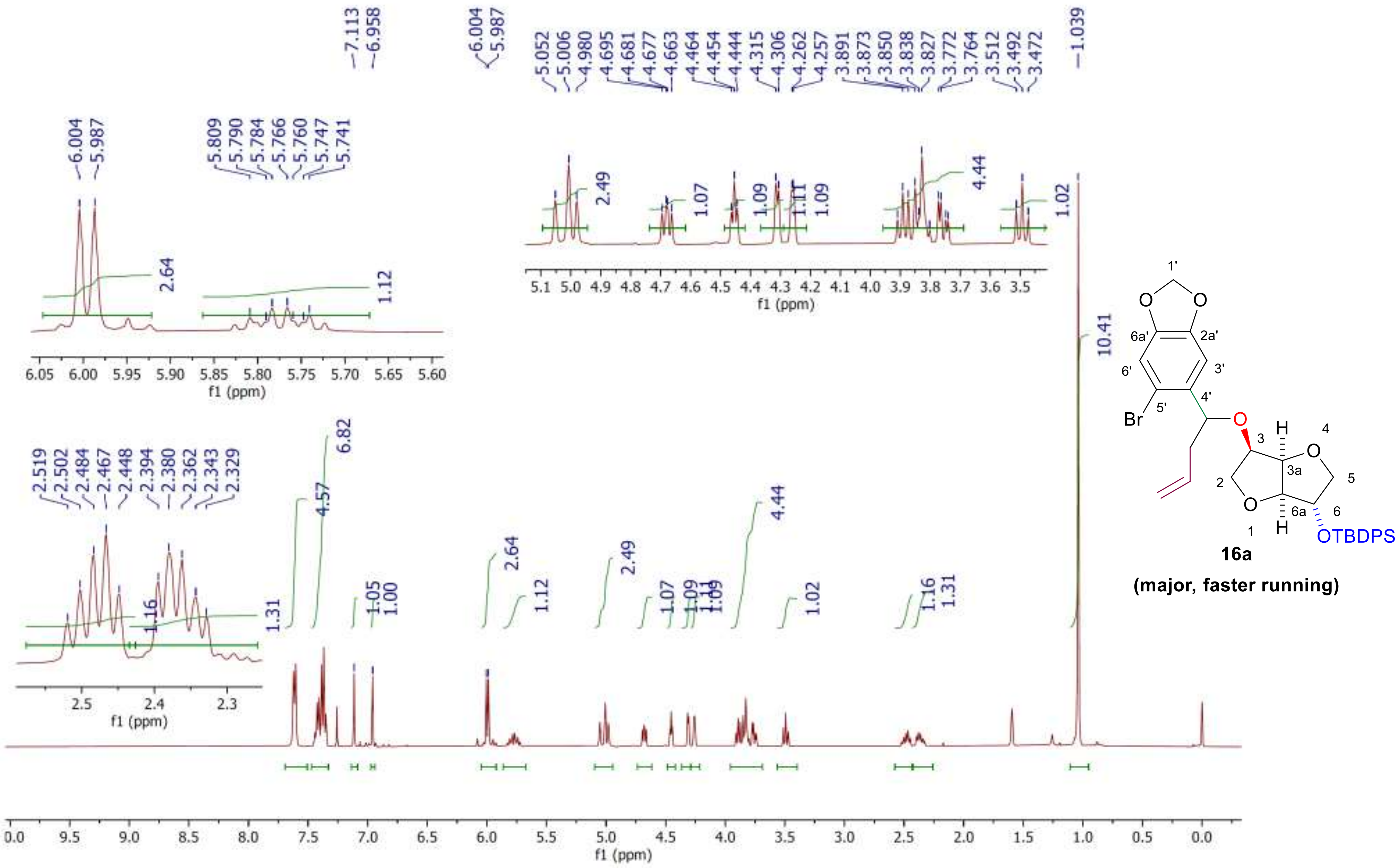

16a

(major, faster running)

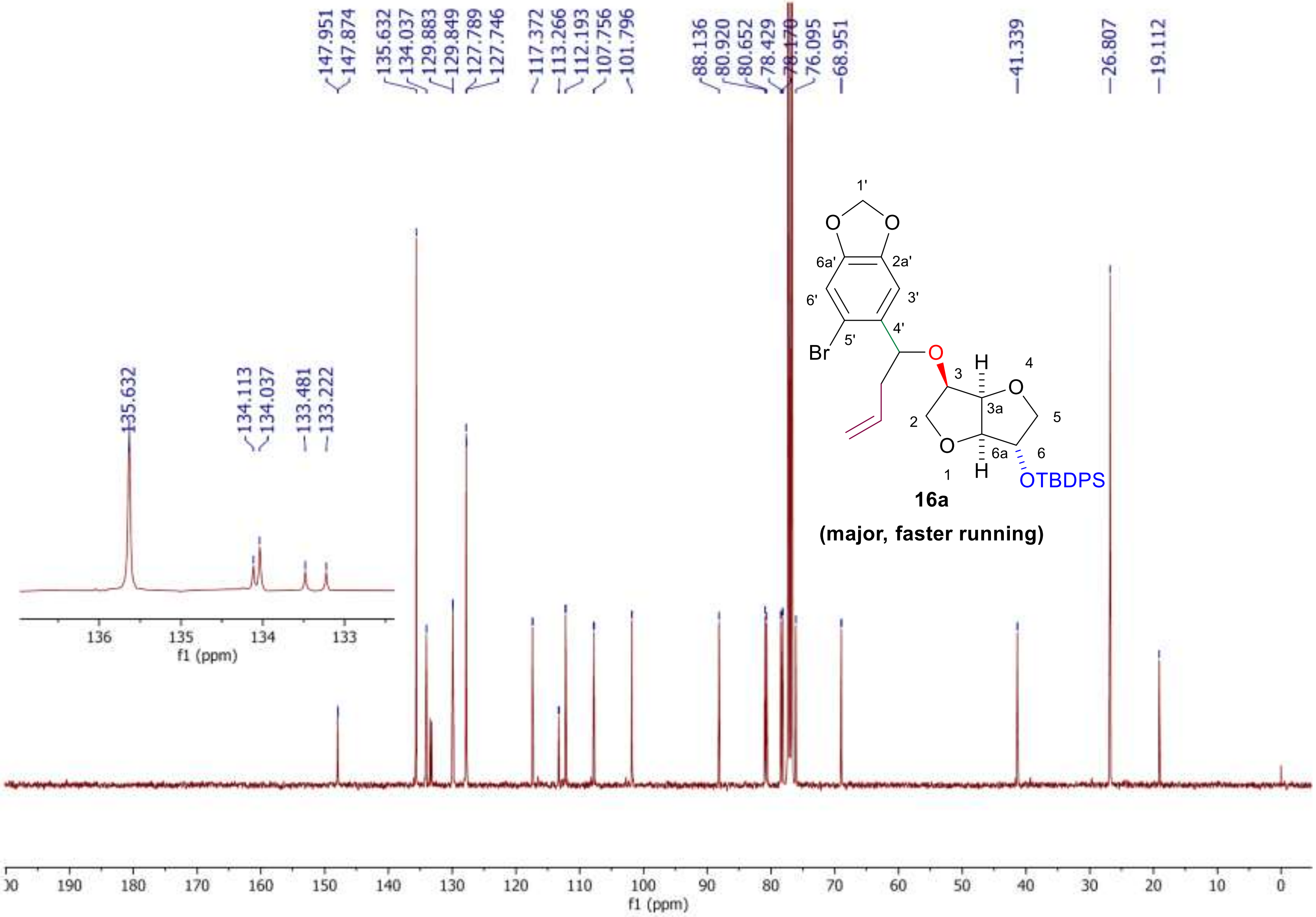

147.951
147.874
135.632
134.037
129.883
129.849
127.789
127.746
117.372
113.266
112.193
107.756
101.796
88.136
80.920
80.652
78.429
78.170
76.095
68.951
41.339
26.807
19.112
135.632
134.113
134.037
133.481
133.222
f1 (ppm)
Br
OTBDPS
16a
(major, faster running)

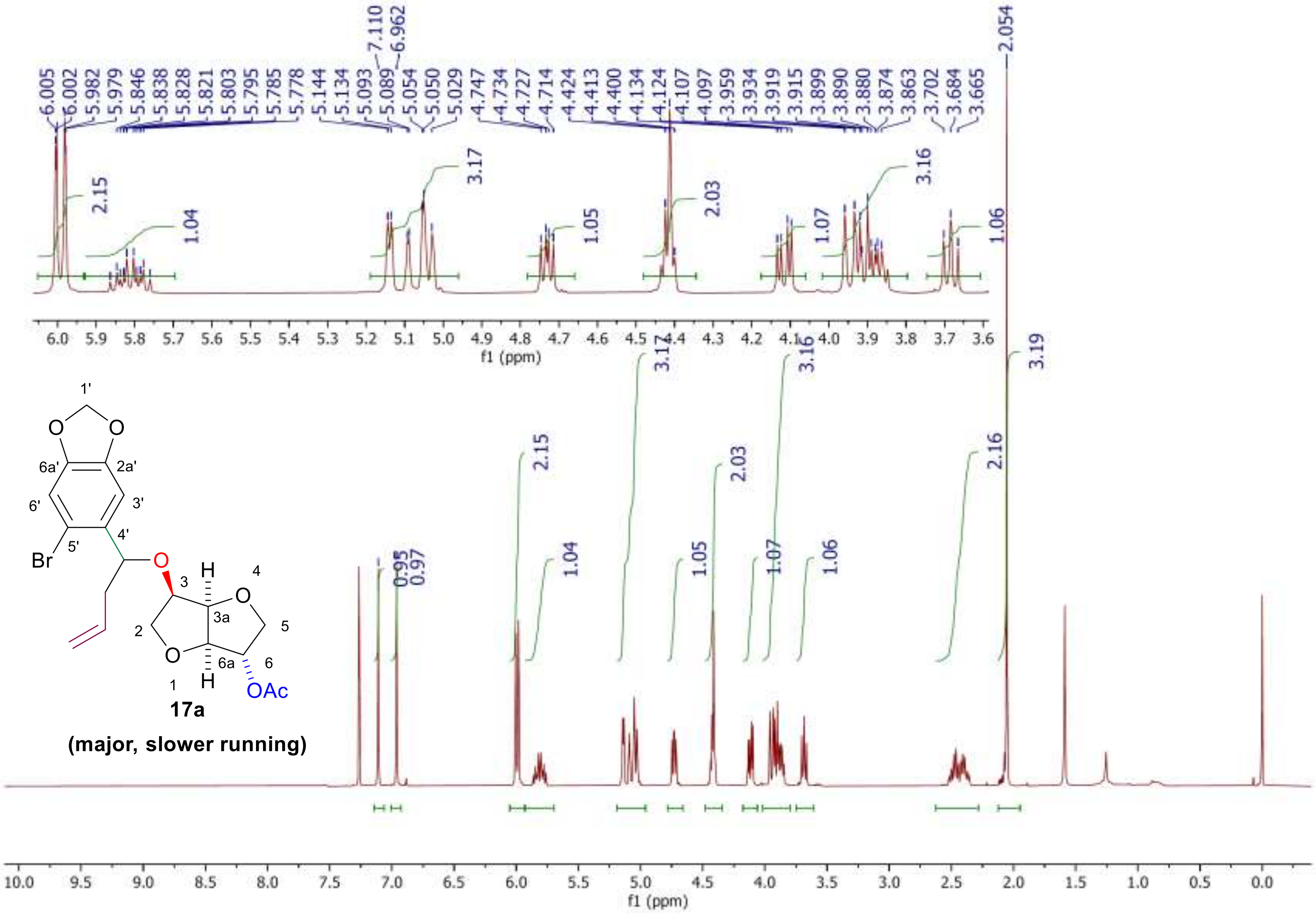

17a

(major, slower running)

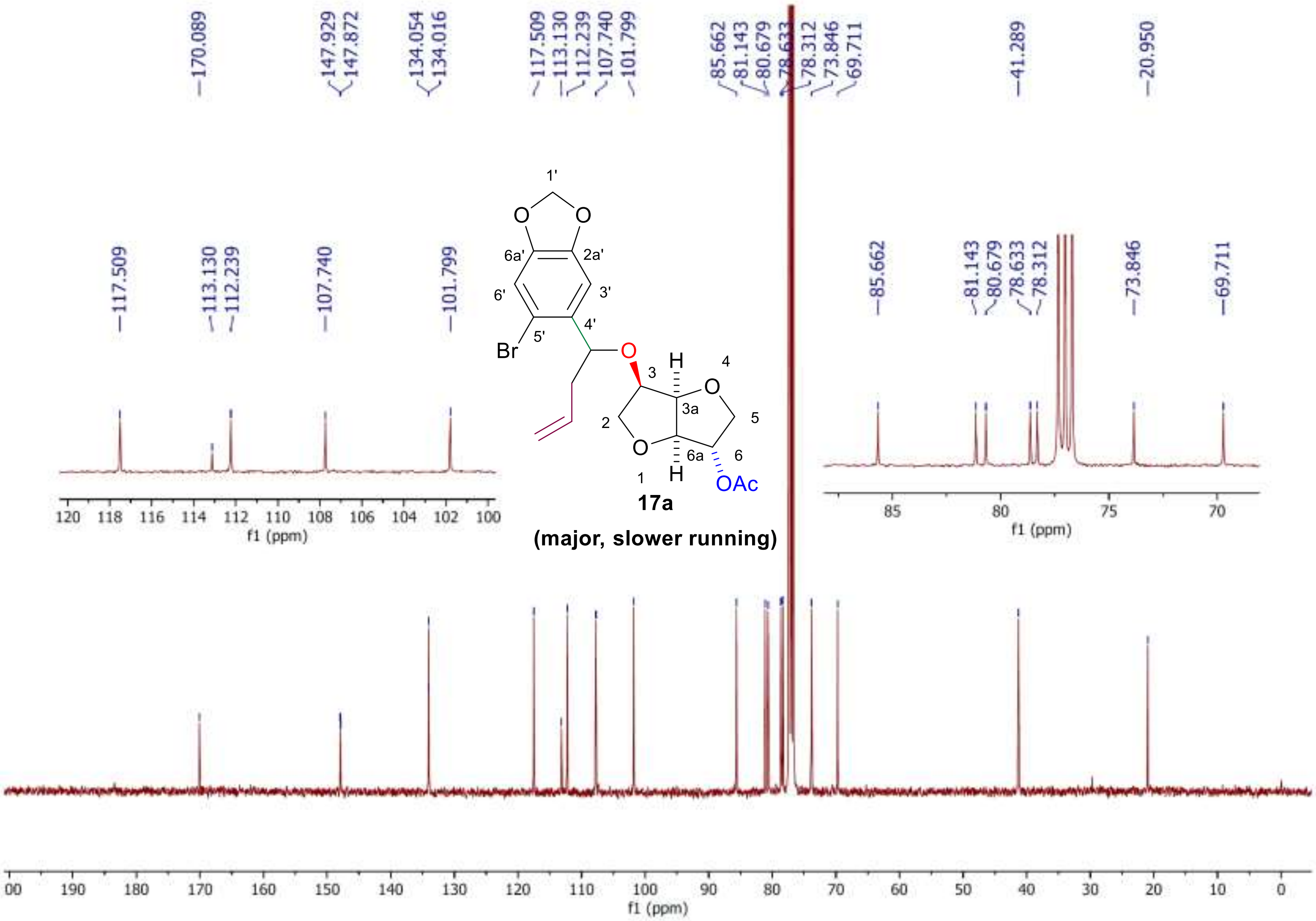

17a

(major, slower running)

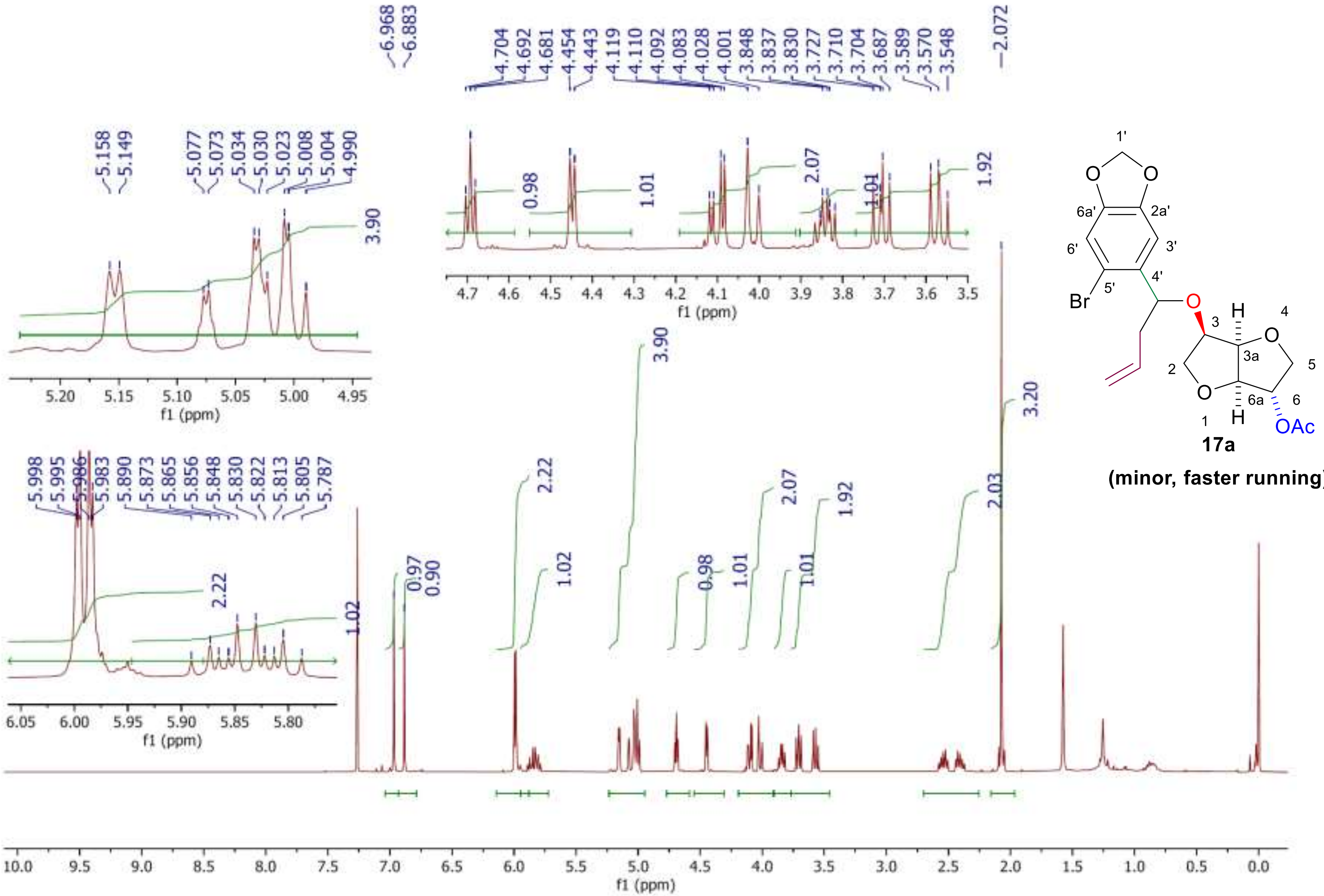

17a

(minor, faster running)

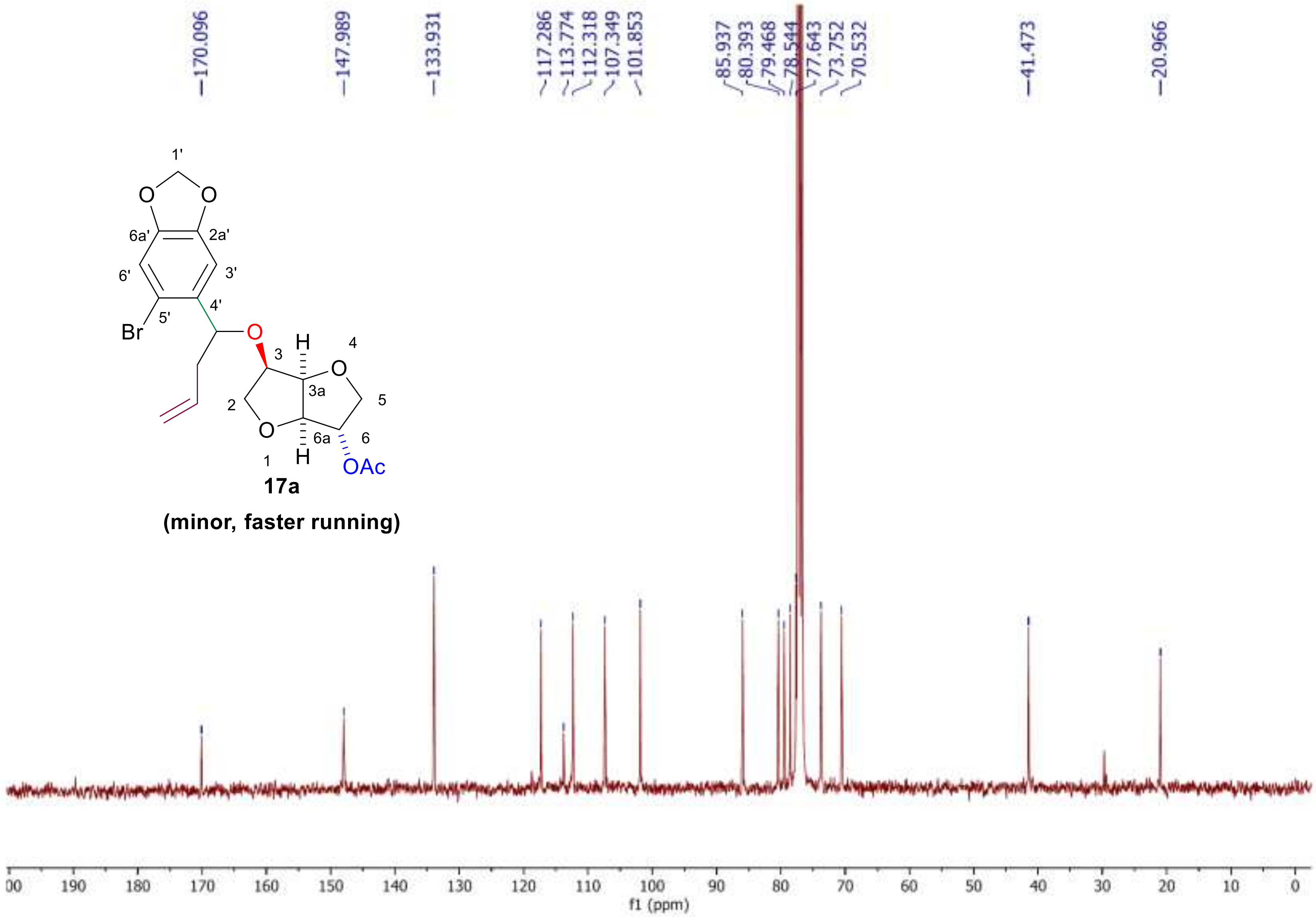

170.096
147.989
133.931
117.286
113.774
112.318
107.349
101.853
85.937
80.393
79.468
78.544
77.643
73.752
70.532
41.473
20.966
1'
6a'
2a'
6'
3'
4'
5'
Br
O
H
4
3
3a
2
5
6a
6
1
H
OAc
17a
(minor, faster running)
f1 (ppm)

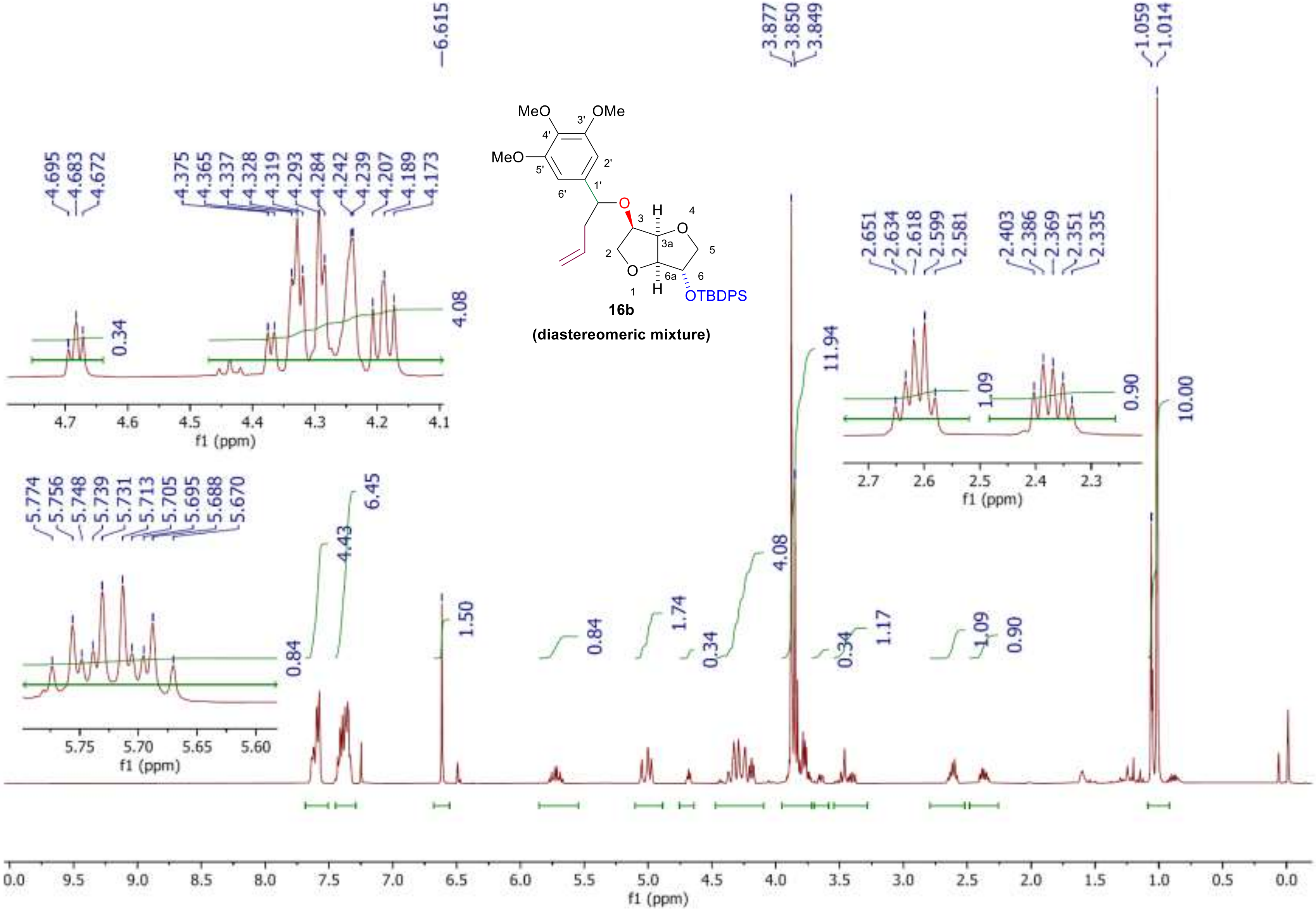
MeO
OMe
MeO
3'
4'
5'
2'
6'
1'
O
3
H
4
O
3a
2
5
O
6a
6
H
1
OTBDPS
16b
(diastereomeric mixture)
f1 (ppm)

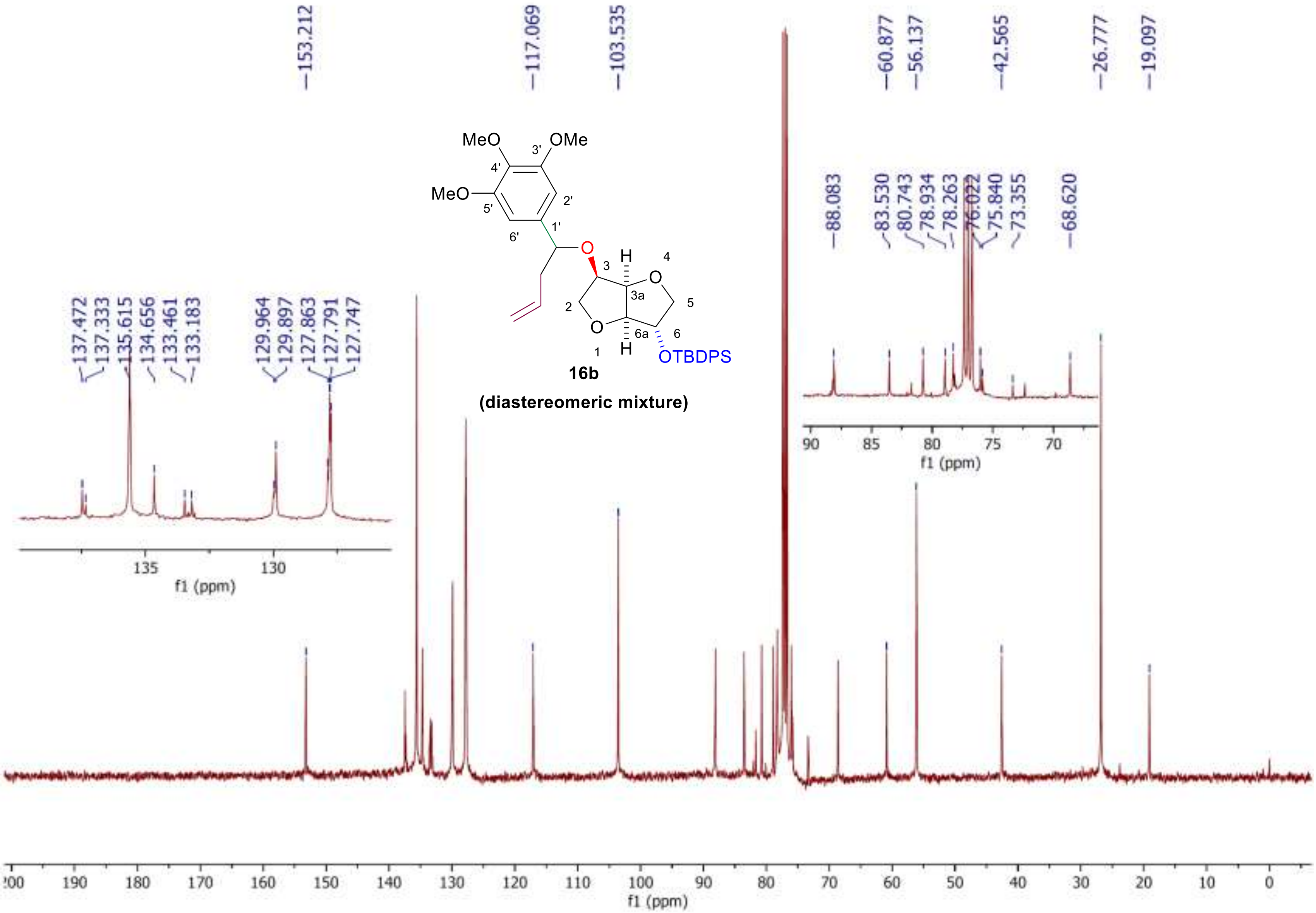

153.212
117.069
103.535
60.877
56.137
42.565
26.777
19.097
MeO
OMe
MeO
3'
4'
5'
2'
6'
1'
3
4
3a
2
5
6
6a
1
H
H
O
O
O
OTBDPS
16b
(diastereomeric mixture)
137.472
137.333
135.615
134.656
133.461
133.183
129.964
129.897
127.863
127.791
127.747
88.083
83.530
80.743
78.934
78.263
76.022
75.840
73.355
68.620
f1 (ppm)

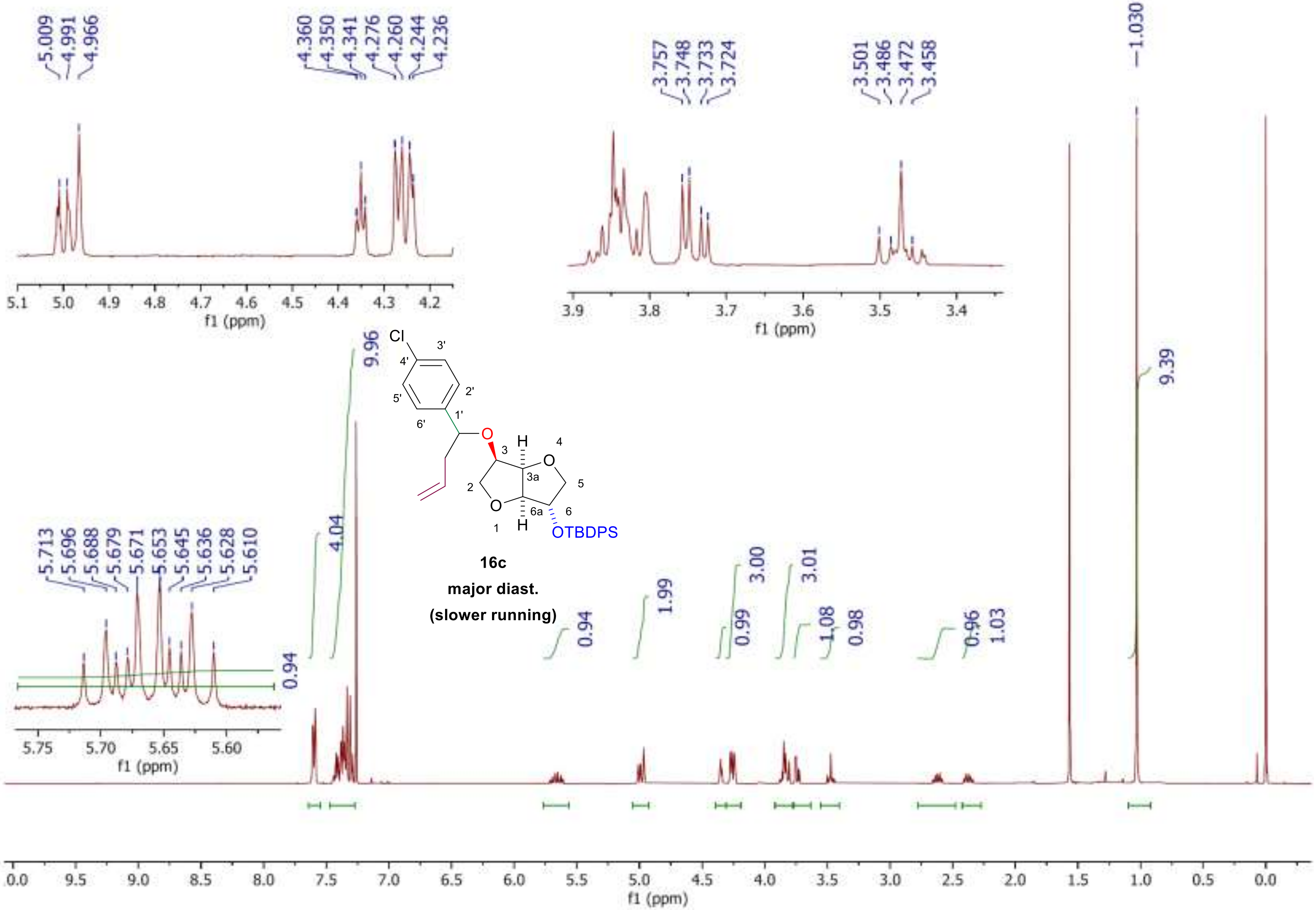

5.009
4.991
4.966
4.360
4.350
4.341
4.276
4.260
4.244
4.236
3.757
3.748
3.733
3.724
3.501
3.486
3.472
3.458
1.030
5.713
5.696
5.688
5.679
5.671
5.653
5.645
5.636
5.628
5.610
9.96
4.04
0.94
0.94
1.99
0.99
3.00
3.01
1.08
0.98
0.96
1.03
9.39
f1 (ppm)
Cl
OTBDPS
16c
major diast.
(slower running)

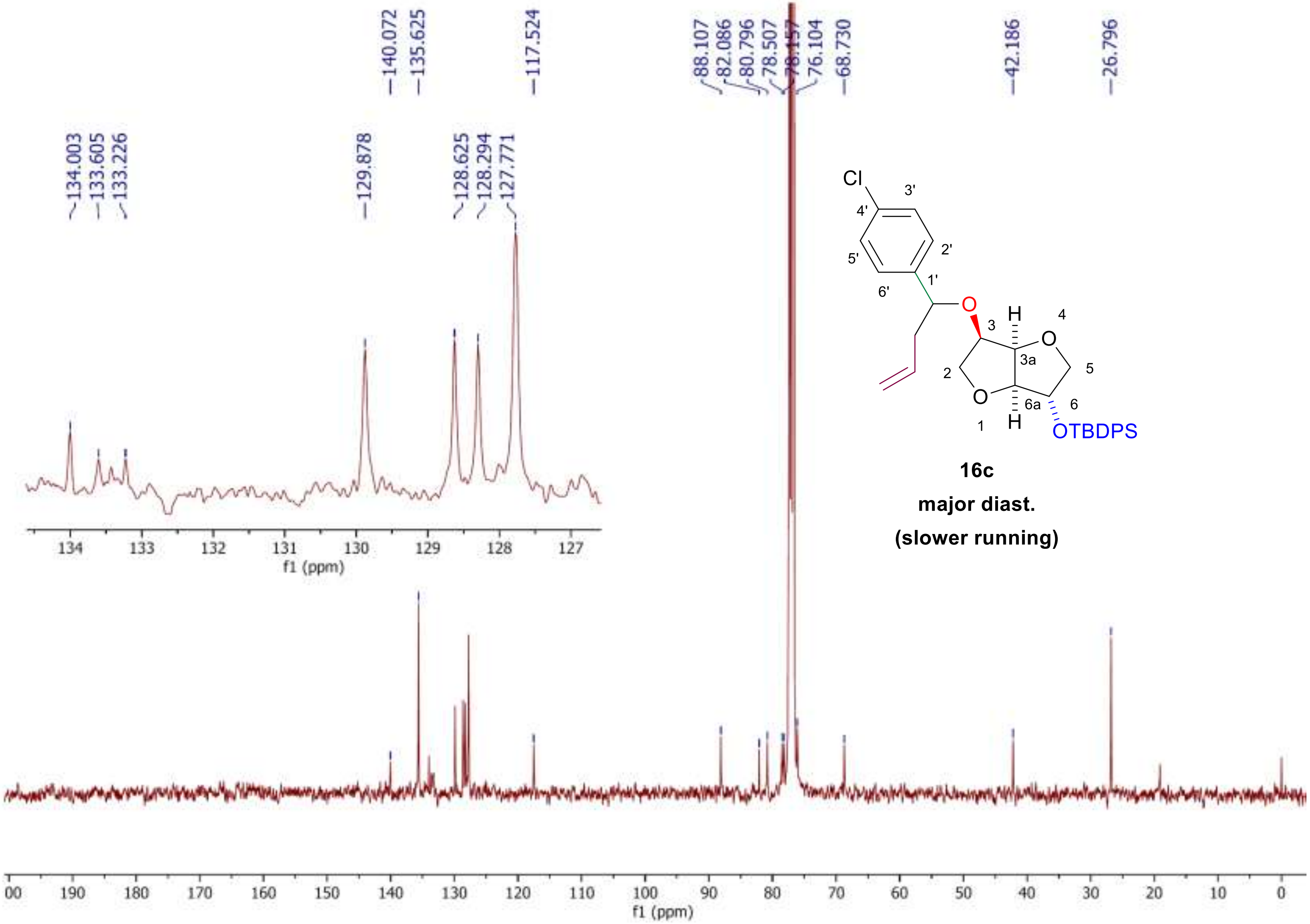

134.003
133.605
133.226
129.878
140.072
135.625
128.625
128.294
127.771
117.524
88.107
82.086
80.796
78.507
78.157
76.104
68.730
42.186
26.796
f1 (ppm)
Cl
OTBDPS
16c
major diast.
(slower running)

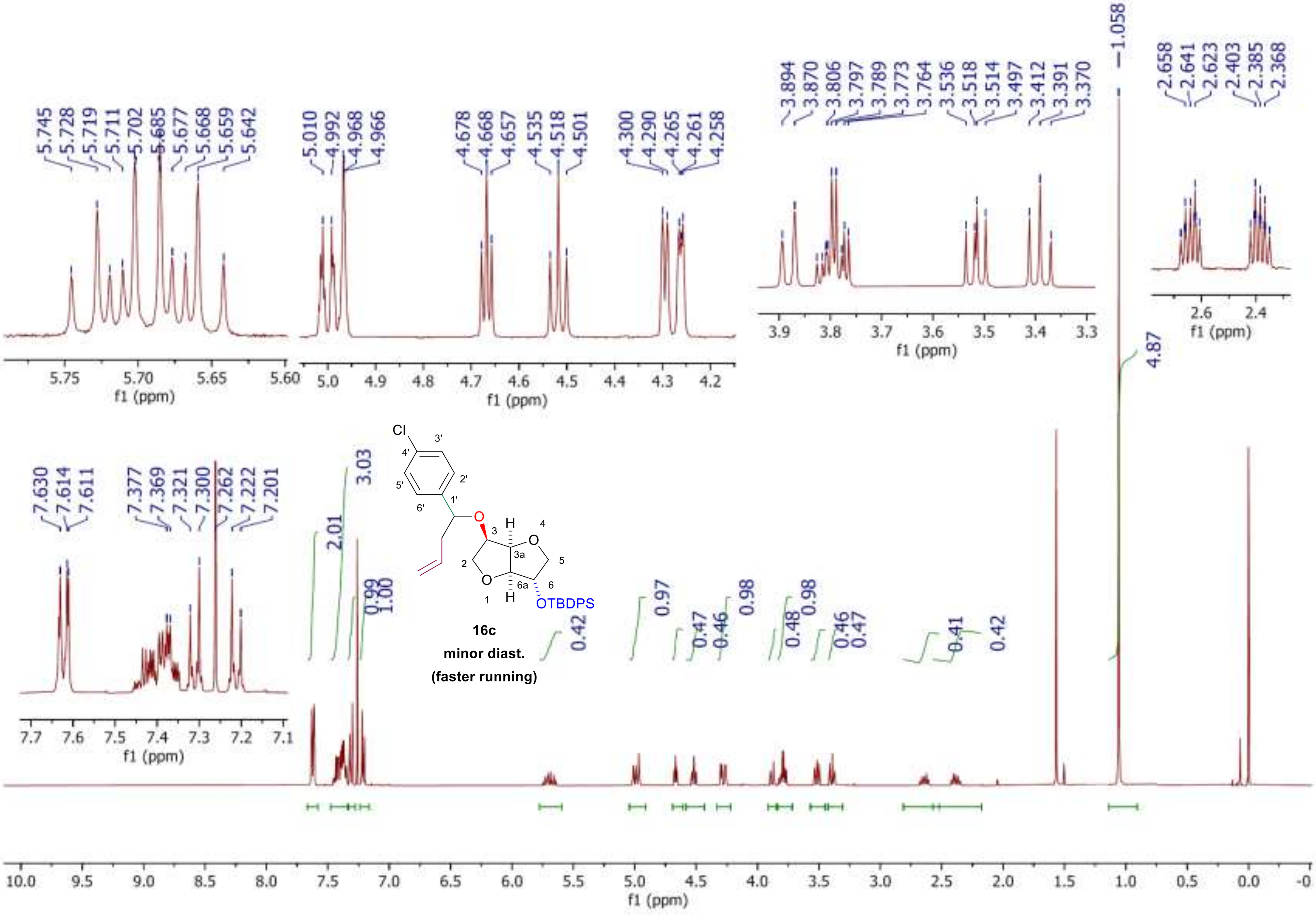

16c
minor diast.
(faster running)

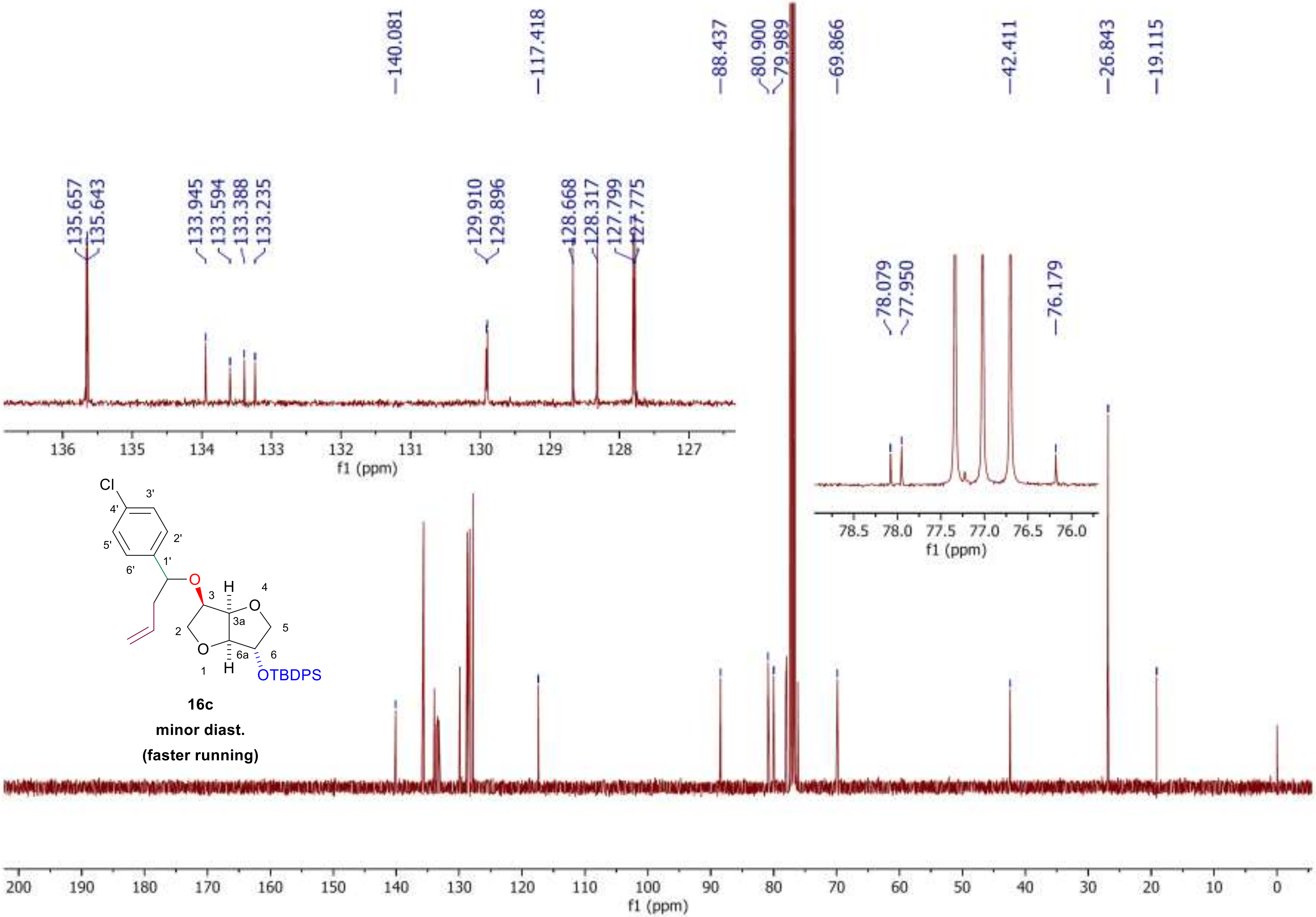

16c
minor diast.
(faster running)

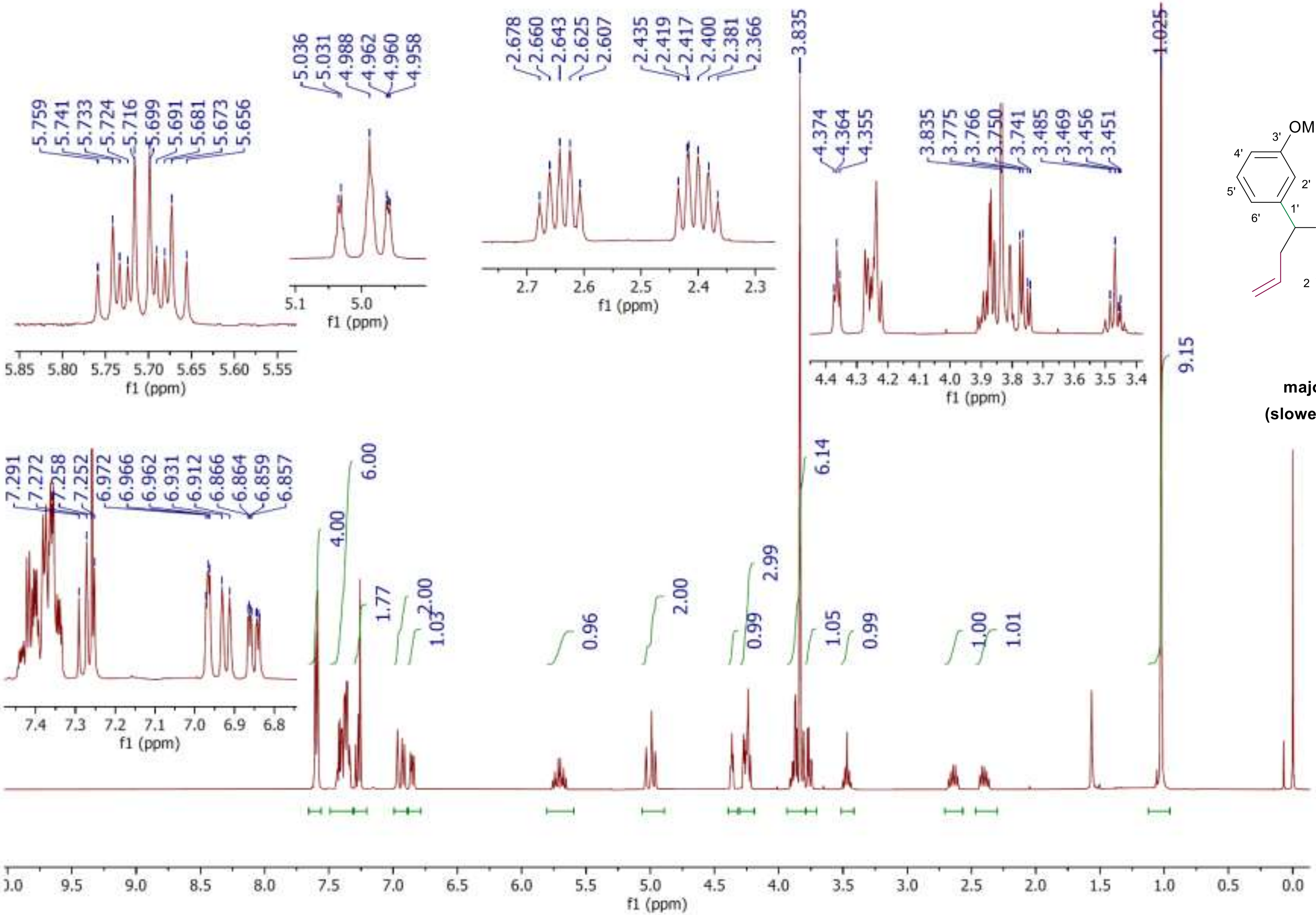

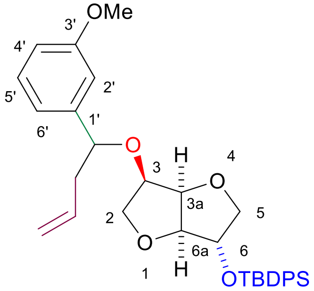

16d

major diast.

(slower running)

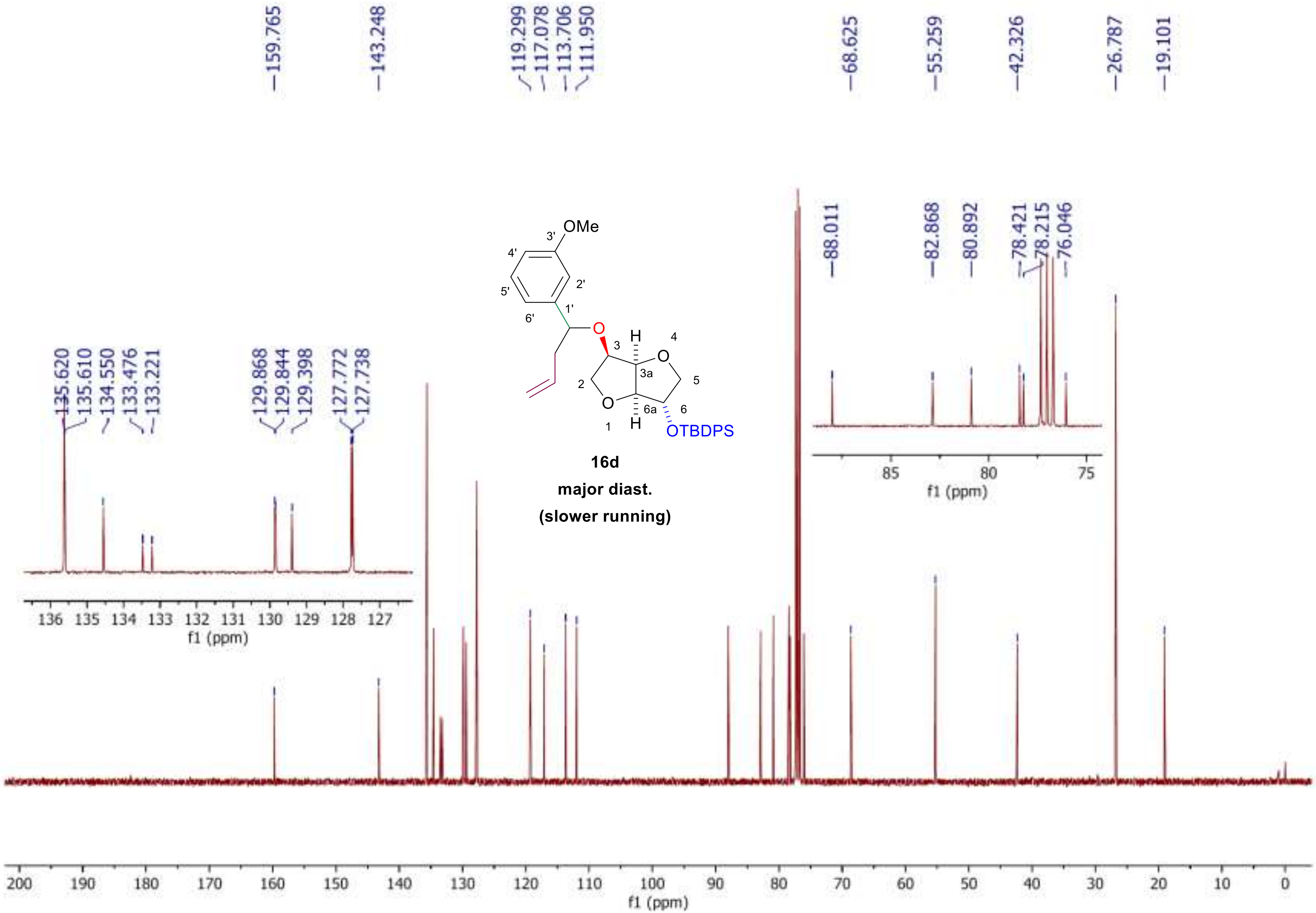

159.765
143.248
119.299
117.078
113.706
111.950
68.625
55.259
42.326
26.787
19.101
135.620
135.610
134.550
133.476
133.221
129.868
129.844
129.398
127.772
127.738
88.011
82.868
80.892
78.421
78.215
76.046
OMe
3'
4'
2'
5'
6'
1'
O
H
4
3
3a
2
5
O
6a
6
1
H
OTBDPS
16d
major diast.
(slower running)
f1 (ppm)

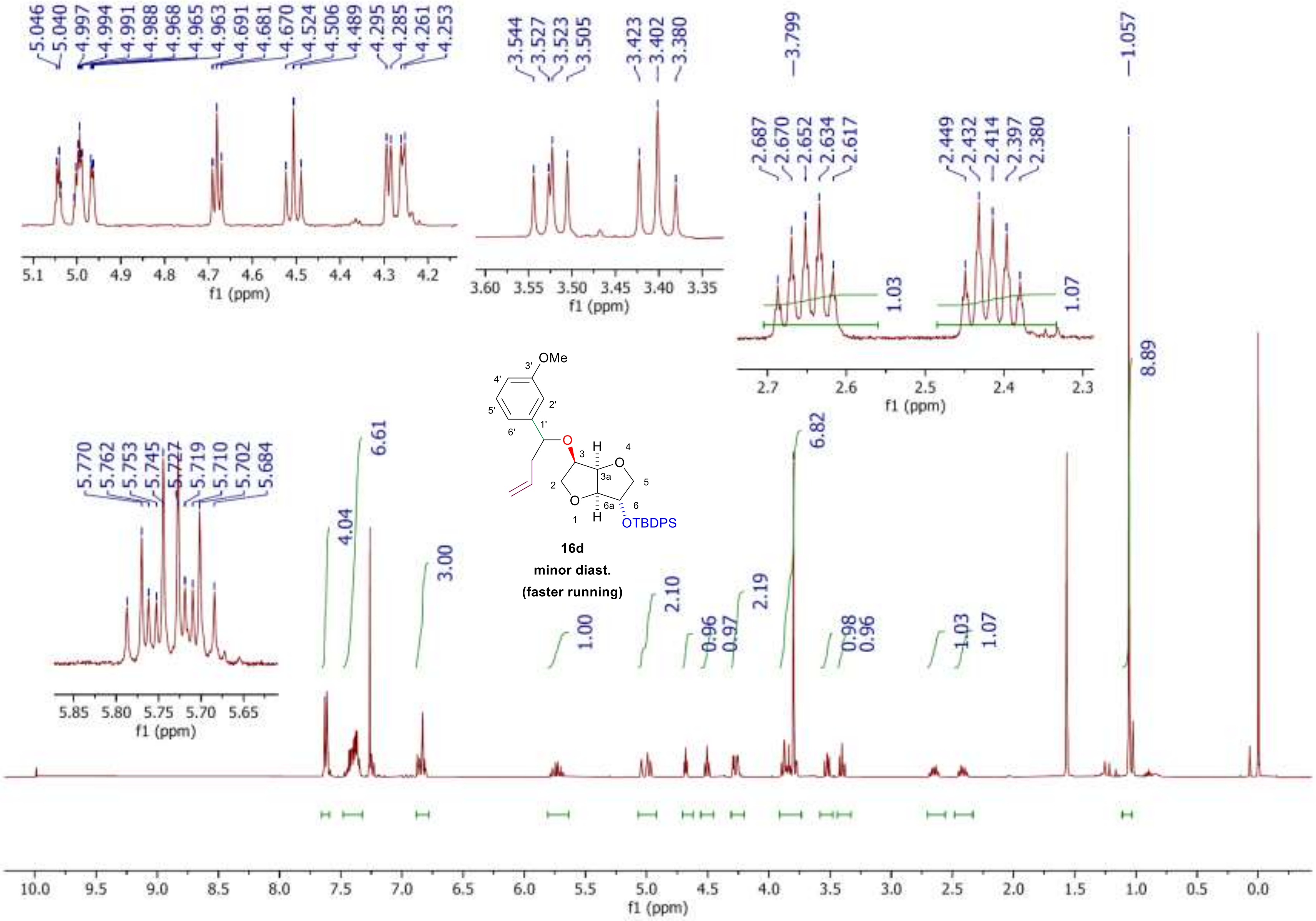
OMe
OTBDPS
16d
minor diast.
(faster running)
f1 (ppm)

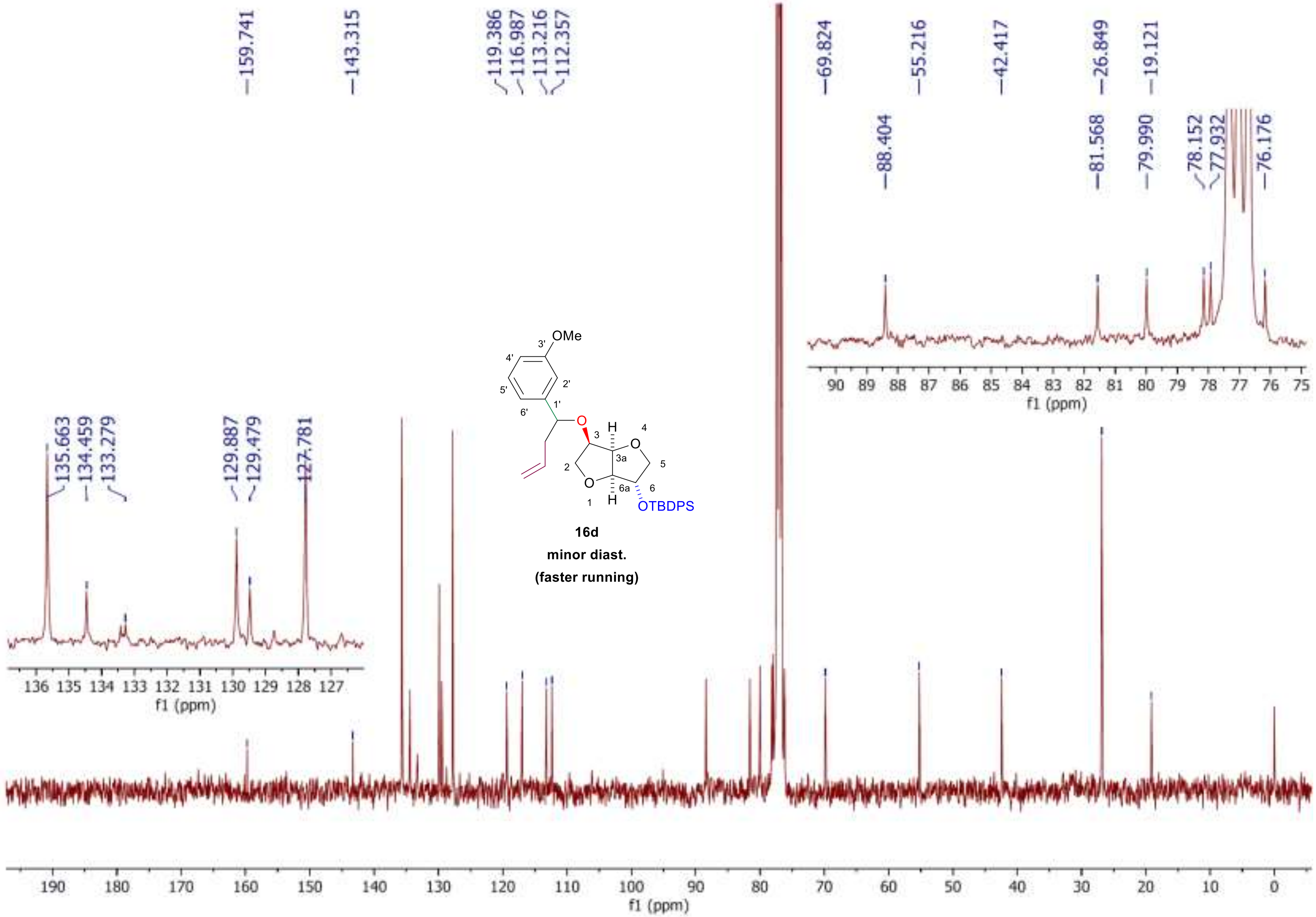
159.741
143.315
119.386
116.987
113.216
112.357
69.824
55.216
42.417
26.849
19.121
88.404
81.568
79.990
78.152
77.932
76.176
135.663
134.459
133.279
129.887
129.479
127.781
OMe
3'
4'
2'
5'
1'
6'
O
3
H
4
O
3a
5
2
O
6a
6
H
1
OTBDPS
16d
minor diast.
(faster running)
f1 (ppm)

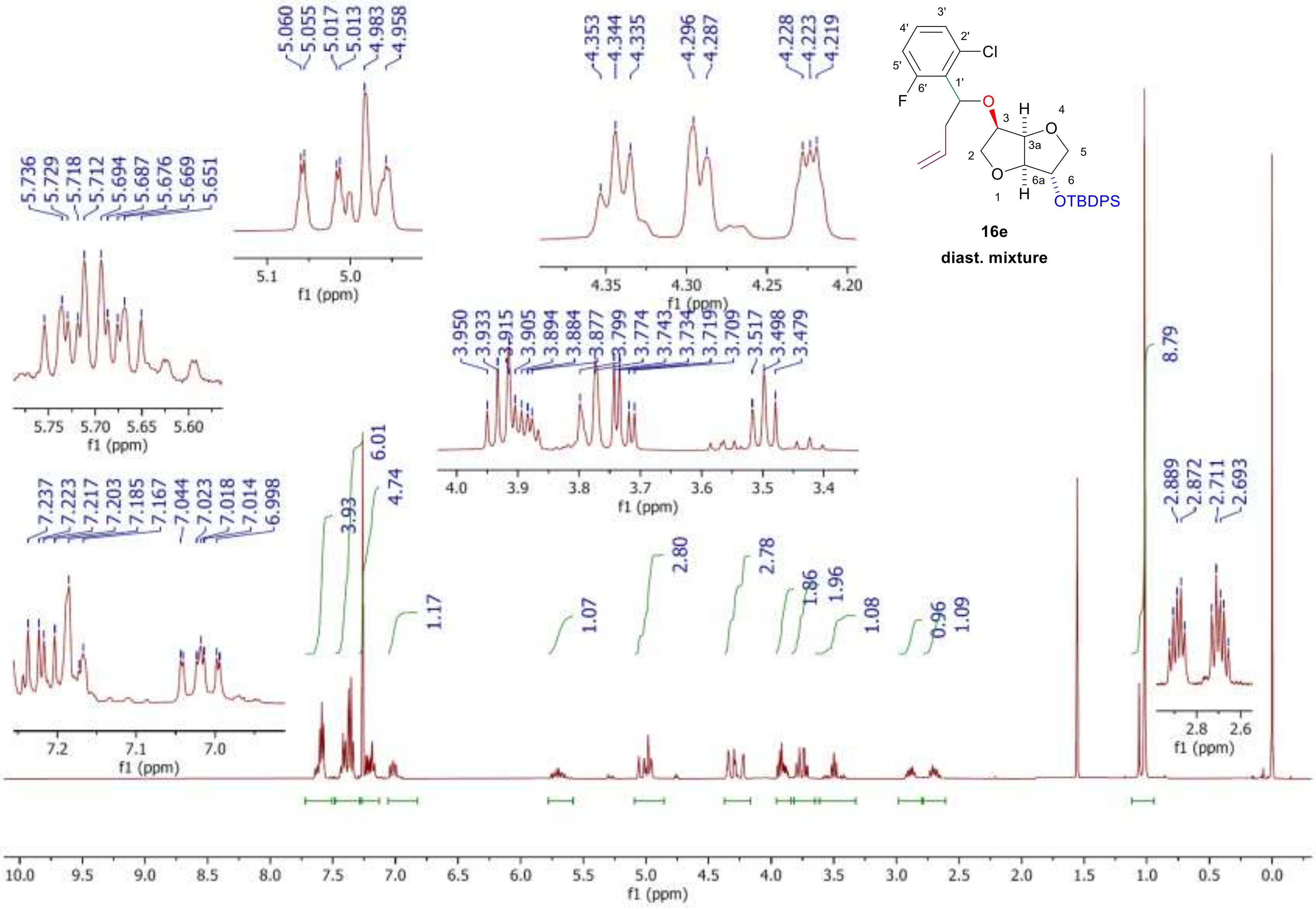

16e
diast. mixture
f1 (ppm)

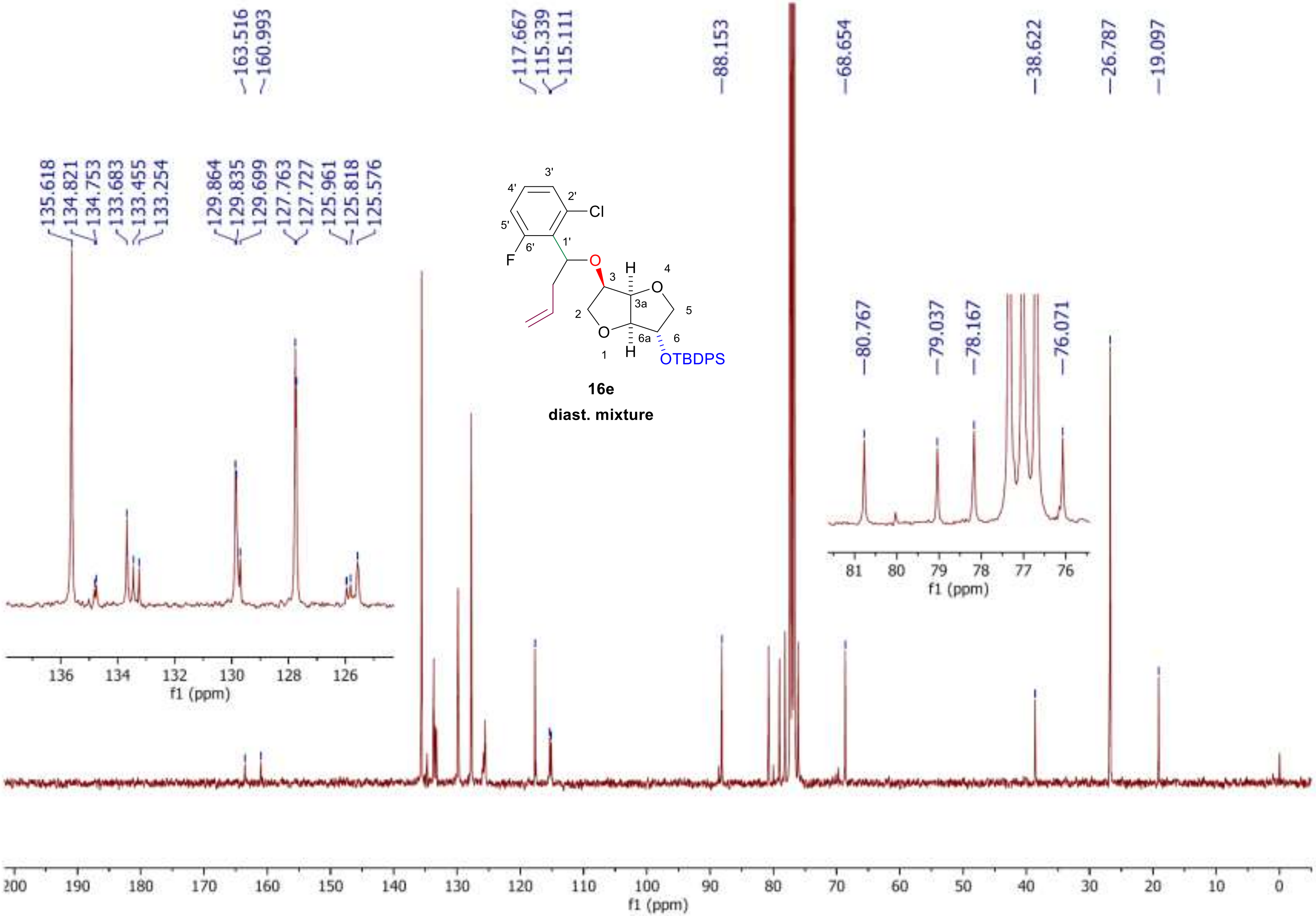
163.516
160.993
117.667
115.339
115.111
88.153
68.654
38.622
26.787
19.097
135.618
134.821
134.753
133.683
133.455
133.254
129.864
129.835
129.699
127.763
127.727
125.961
125.818
125.576
Cl
F
OTBDPS
16e
diast. mixture
80.767
79.037
78.167
76.071
f1 (ppm)

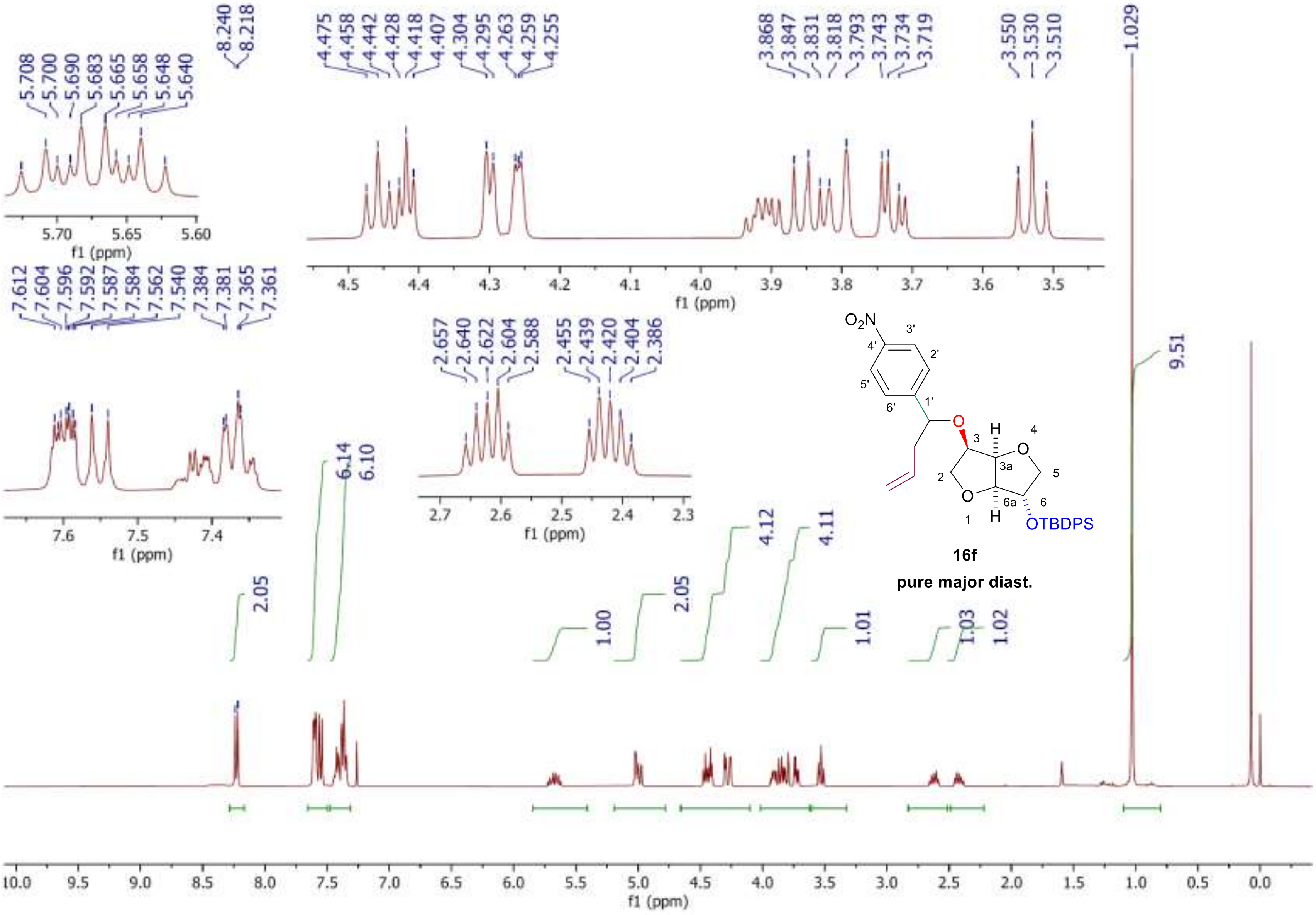
5.708
5.700
5.690
5.683
5.665
5.658
5.648
5.640
8.240
8.218
4.475
4.458
4.442
4.428
4.418
4.407
4.304
4.295
4.263
4.259
4.255
3.868
3.847
3.831
3.818
3.793
3.743
3.734
3.719
3.550
3.530
3.510
1.029
7.612
7.604
7.596
7.592
7.587
7.584
7.562
7.540
7.384
7.381
7.365
7.361
2.657
2.640
2.622
2.604
2.588
2.455
2.439
2.420
2.404
2.386
f1 (ppm)
O2N
OTBDPS
16f
pure major diast.
2.05
6.14
6.10
1.00
2.05
4.12
4.11
1.01
1.03
1.02
9.51

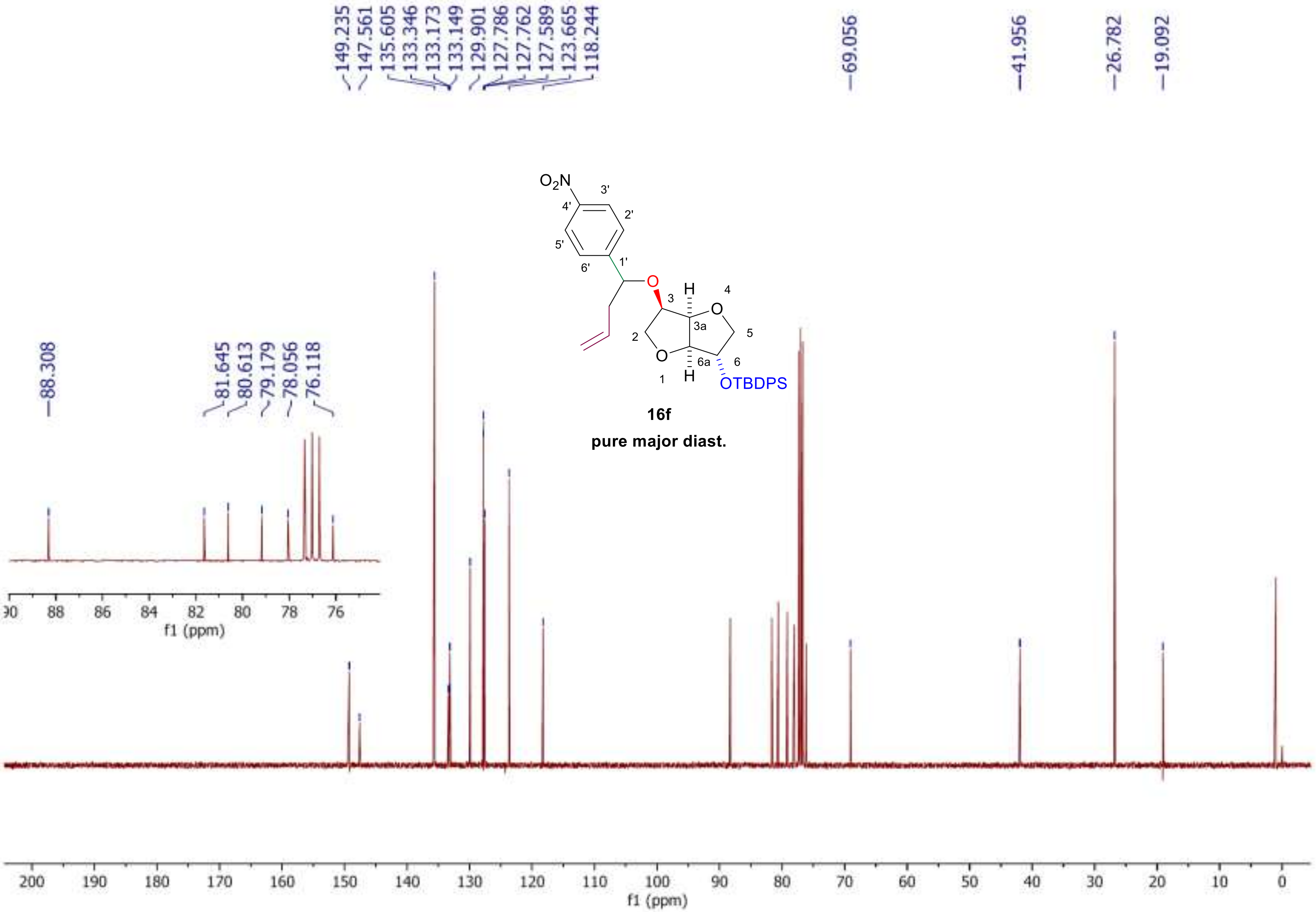
149.235
147.561
135.605
133.346
133.173
133.149
129.901
127.786
127.762
127.589
123.665
118.244
69.056
41.956
26.782
19.092
88.308
81.645
80.613
79.179
78.056
76.118
O_2N
OTBDPS
16f
pure major diast.
f1 (ppm)

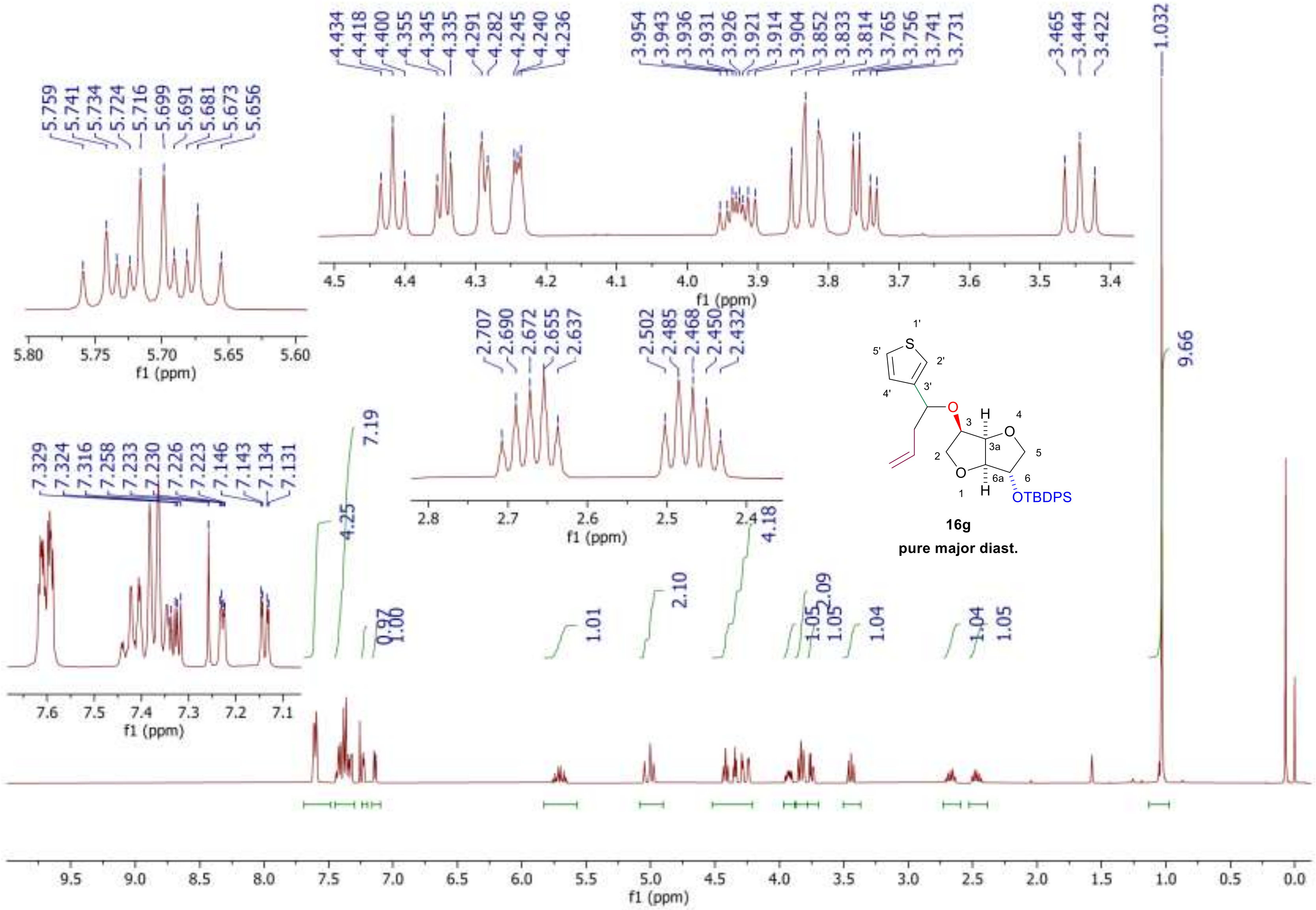

16g
pure major diast.
OTBDPS
f1 (ppm)

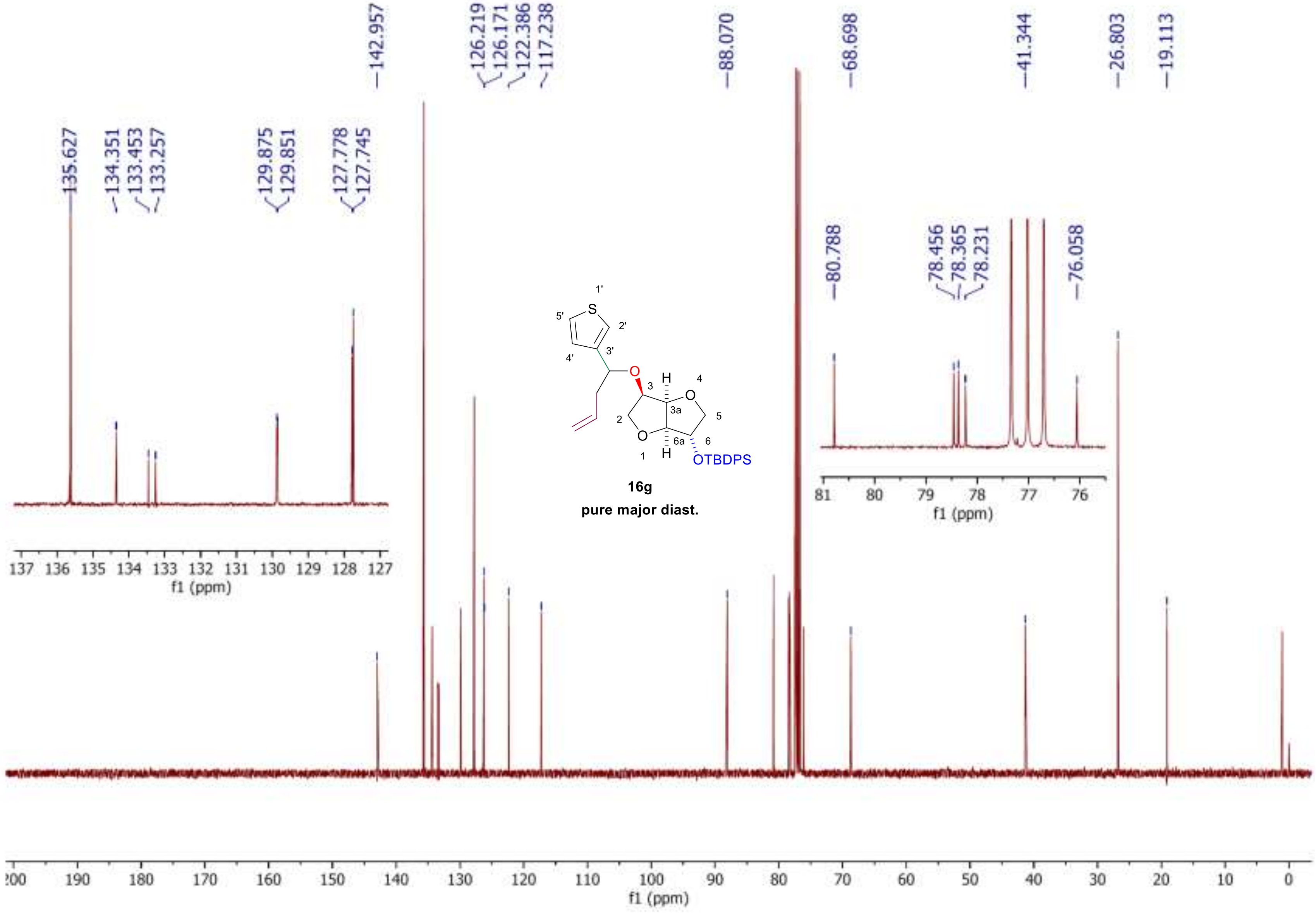

142.957
126.219
126.171
122.386
117.238
88.070
68.698
41.344
26.803
19.113
135.627
134.351
133.453
133.257
129.875
129.851
127.778
127.745
80.788
78.456
78.365
78.231
76.058
16g
pure major diast.
OTBDPS
f1 (ppm)

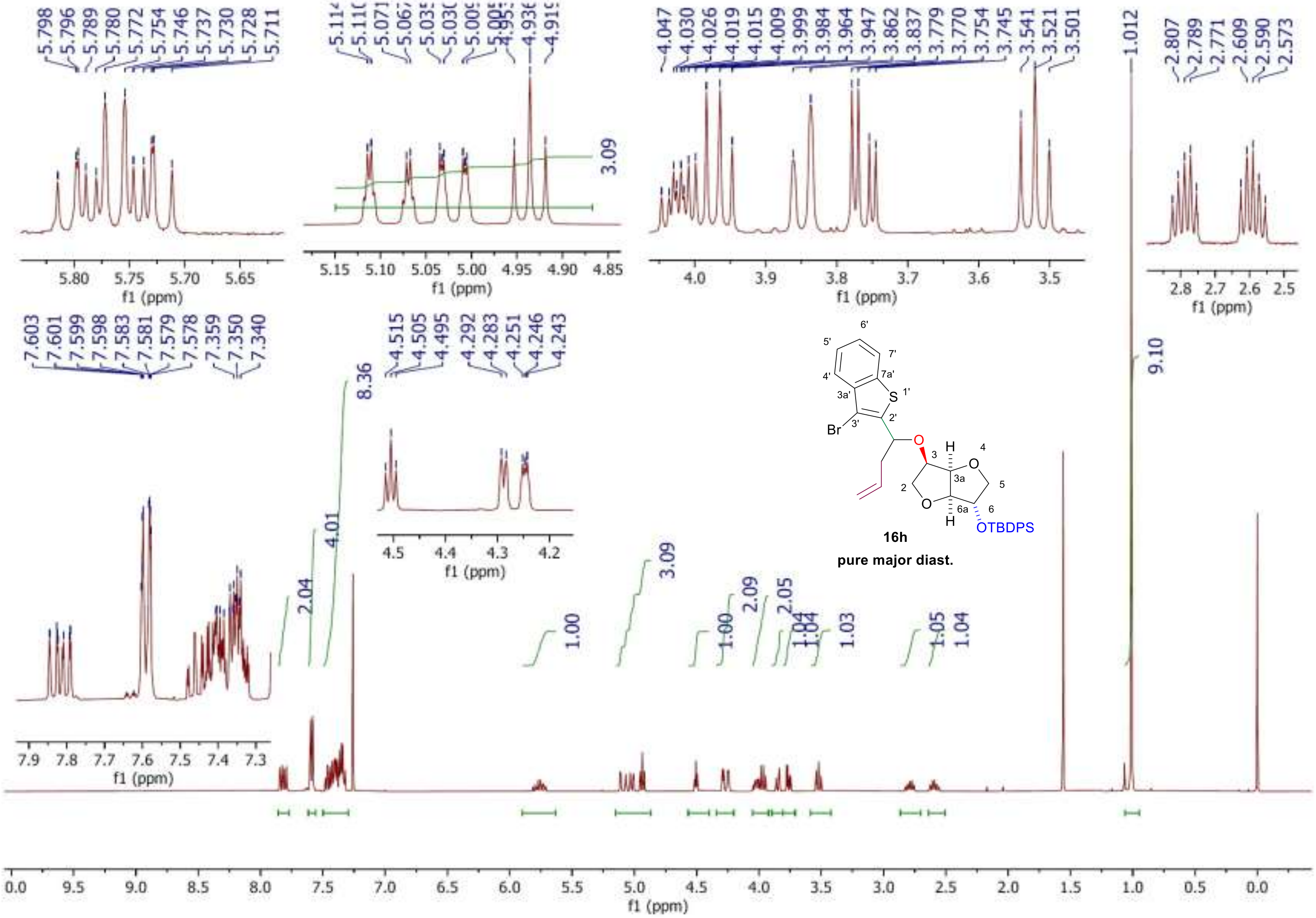
16h
pure major diast.
OTBDPS
Br
f1 (ppm)

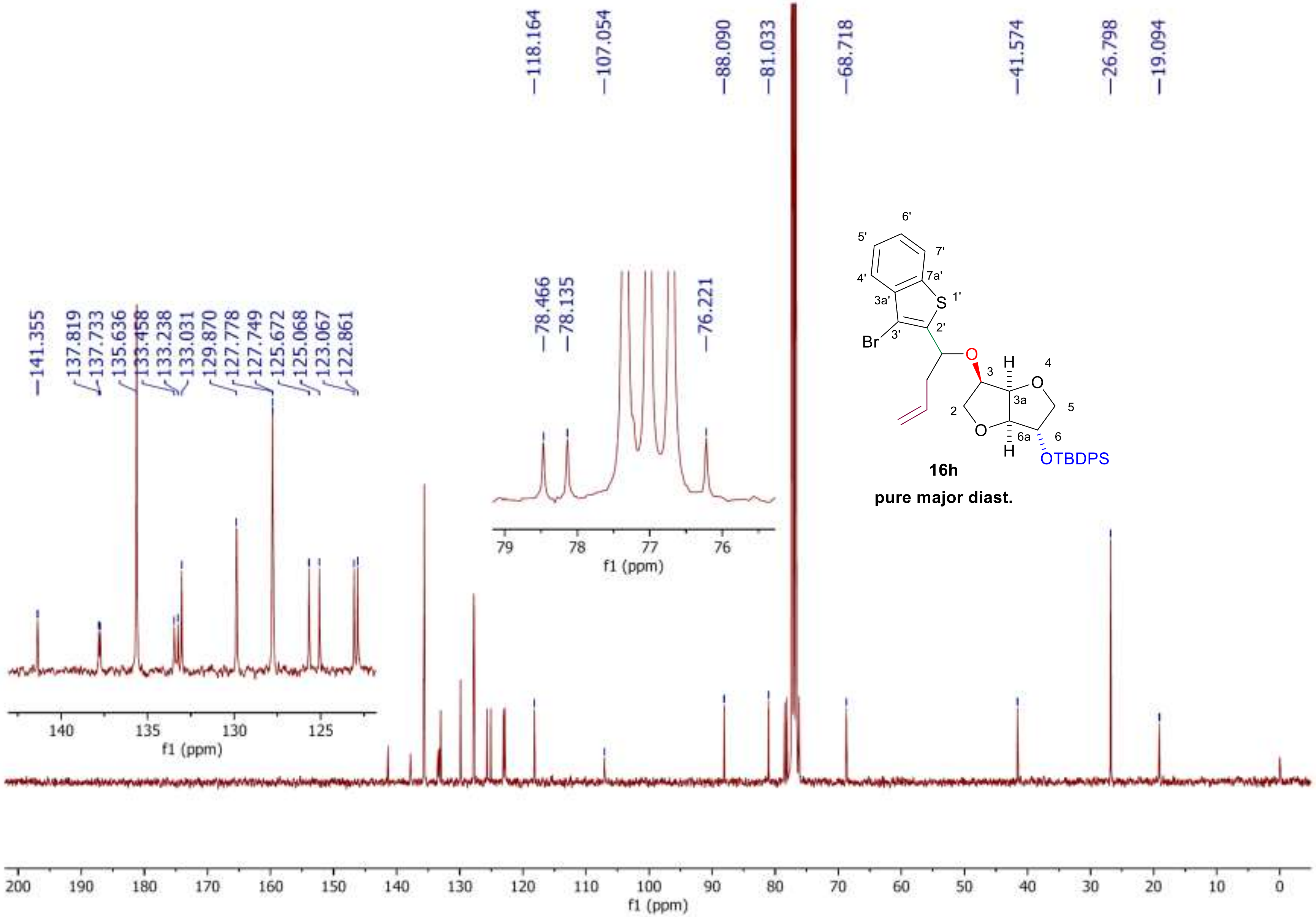

118.164
107.054
88.090
81.033
68.718
41.574
26.798
19.094
141.355
137.819
137.733
135.636
133.458
133.238
133.031
129.870
127.778
127.749
125.672
125.068
123.067
122.861
78.466
78.135
76.221
f1 (ppm)
16h
pure major diast.
OTBDPS

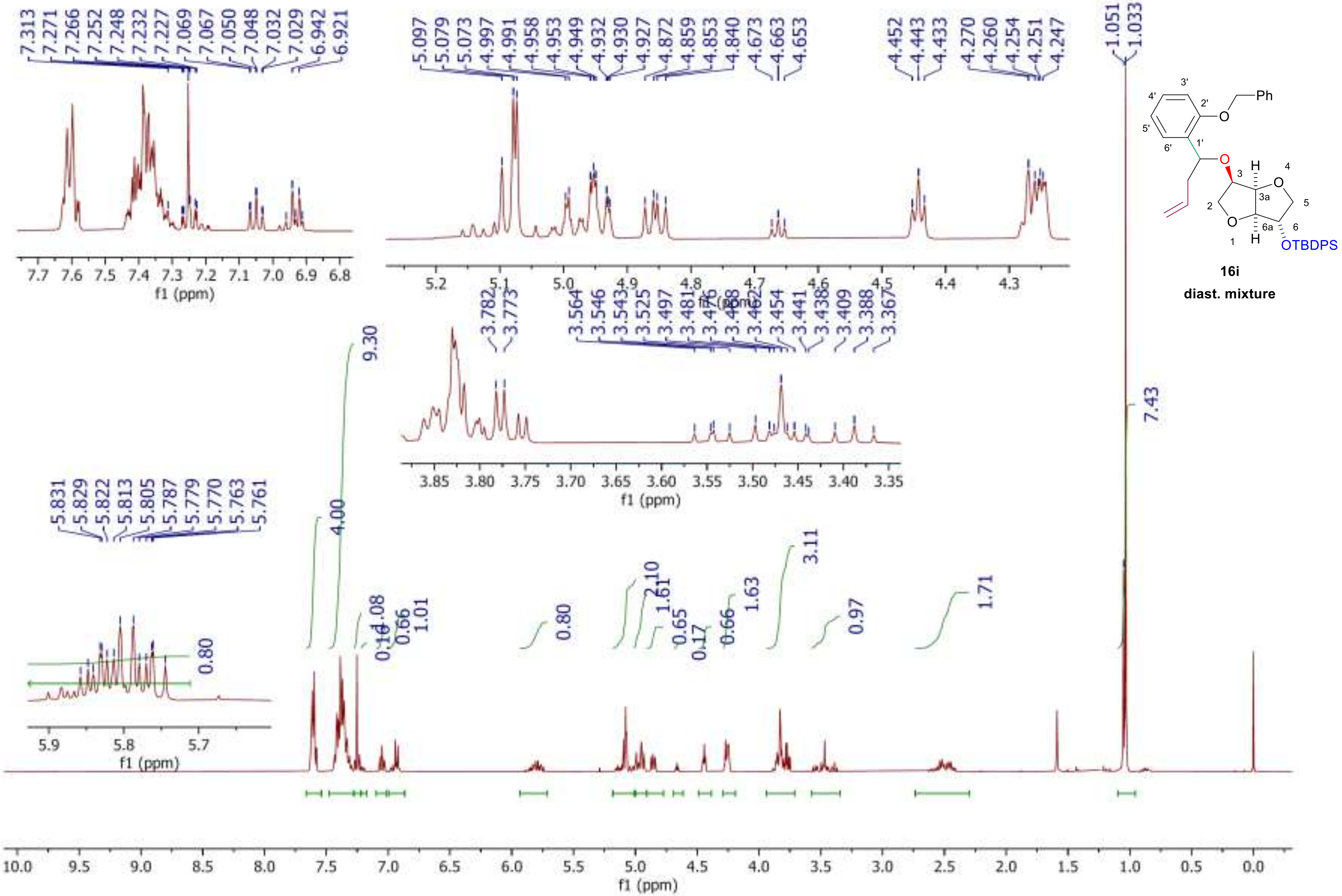

16i
diast. mixture

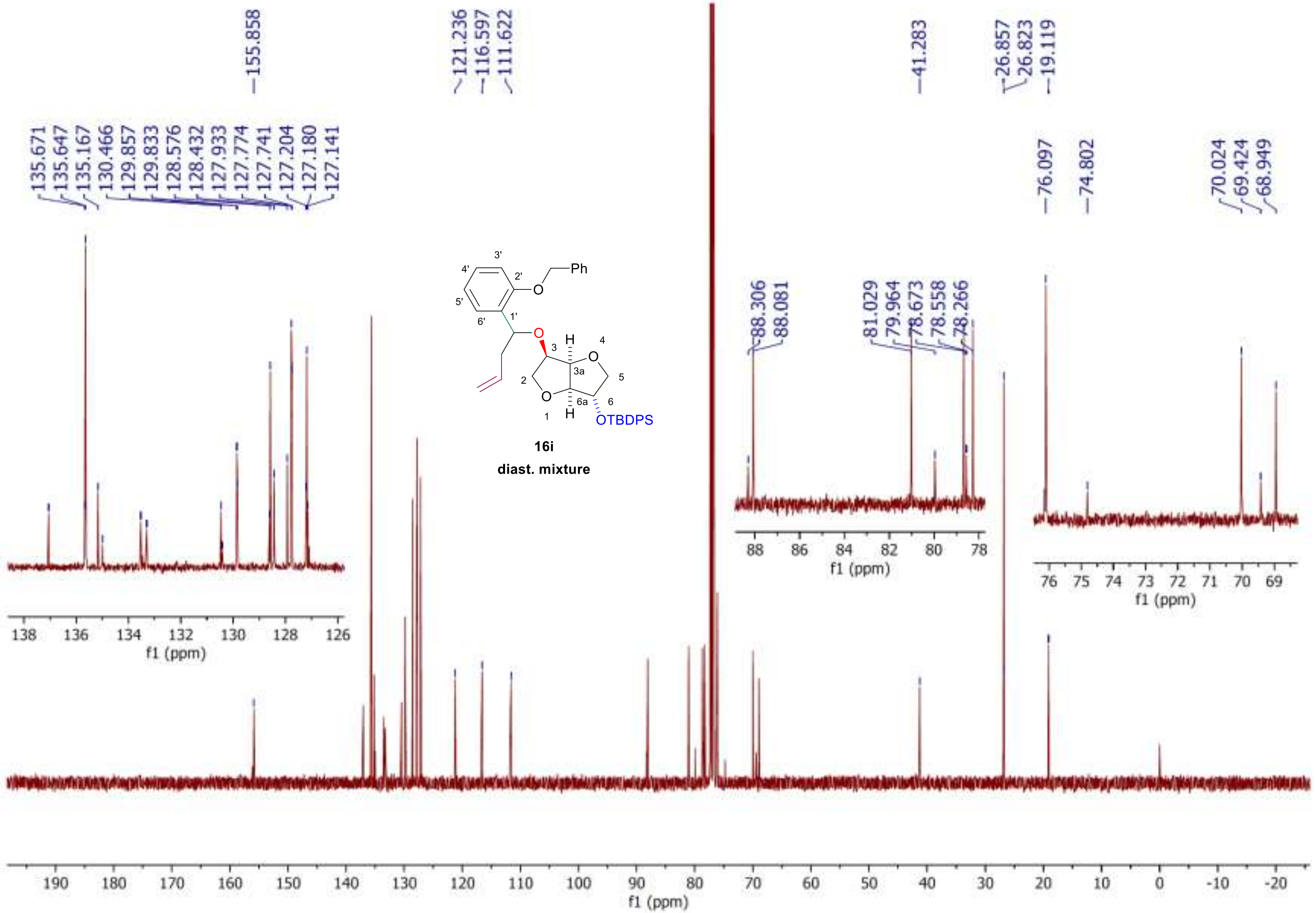

135.671
135.647
135.167
130.466
129.857
129.833
128.576
128.432
127.933
127.774
127.741
127.204
127.180
127.141
155.858
121.236
116.597
111.622
41.283
26.857
26.823
19.119
76.097
74.802
70.024
69.424
68.949
88.306
88.081
81.029
79.964
78.673
78.558
78.266
16i
diast. mixture
OTBDPS
Ph
f1 (ppm)

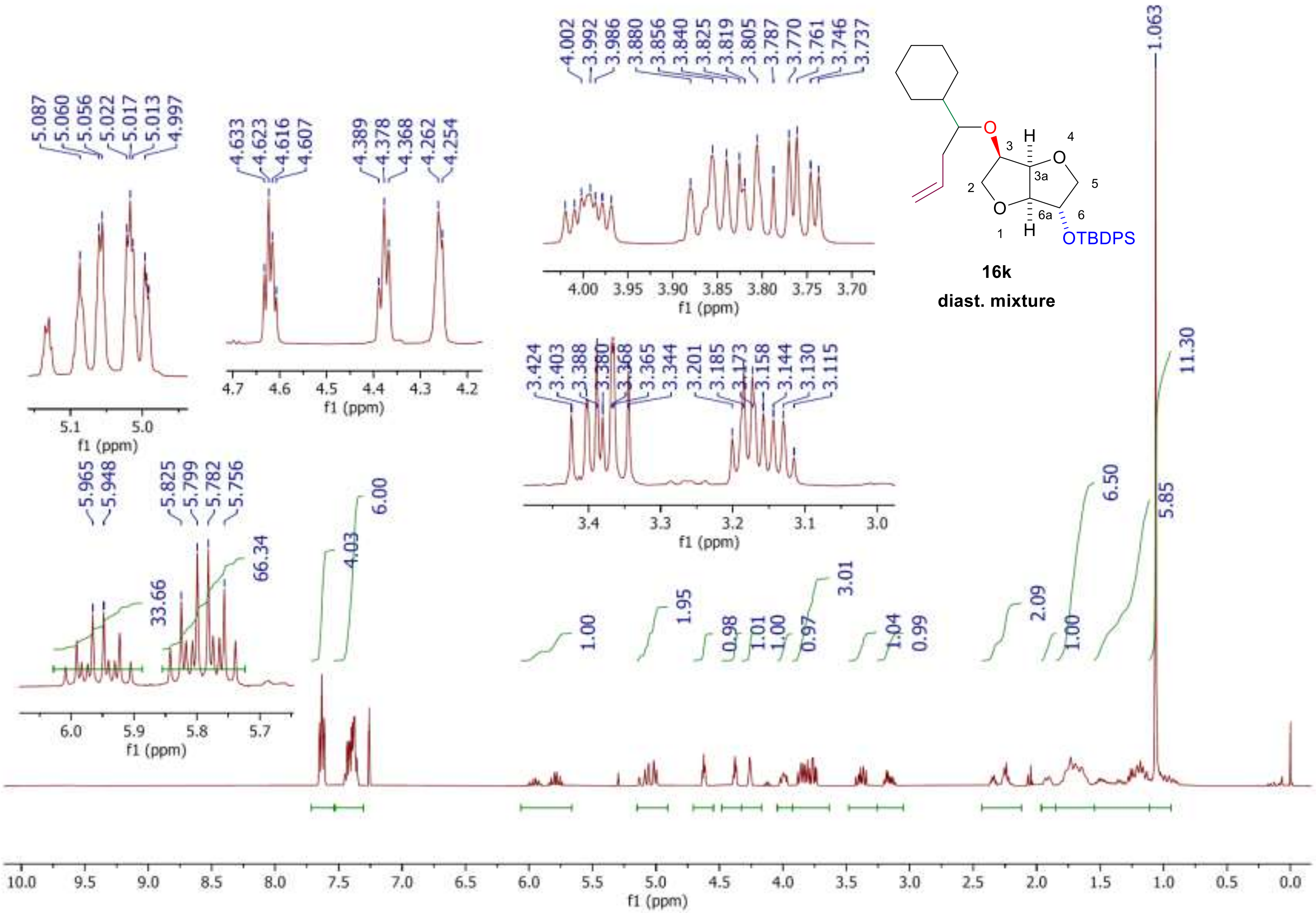

16k
diast. mixture
OTBDPS
f1 (ppm)

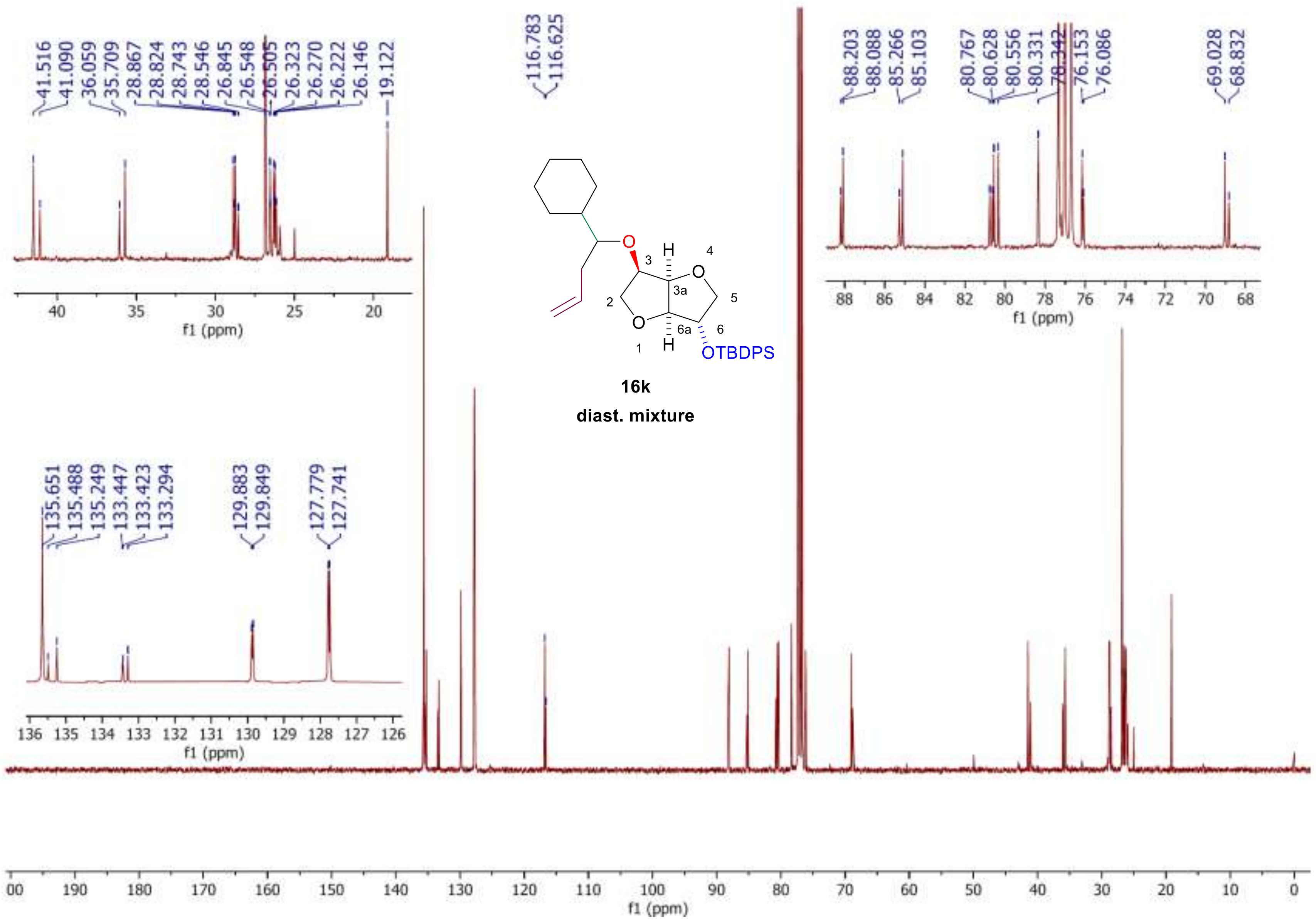

41.516
41.090
36.059
35.709
28.867
28.824
28.743
28.546
26.845
26.548
26.505
26.323
26.270
26.222
26.146
19.122
116.783
116.625
88.203
88.088
85.266
85.103
80.767
80.628
80.556
80.331
78.342
76.153
76.086
69.028
68.832
135.651
135.488
135.249
133.447
133.423
133.294
129.883
129.849
127.779
127.741
OTBDPS
16k
diast. mixture
f1 (ppm)

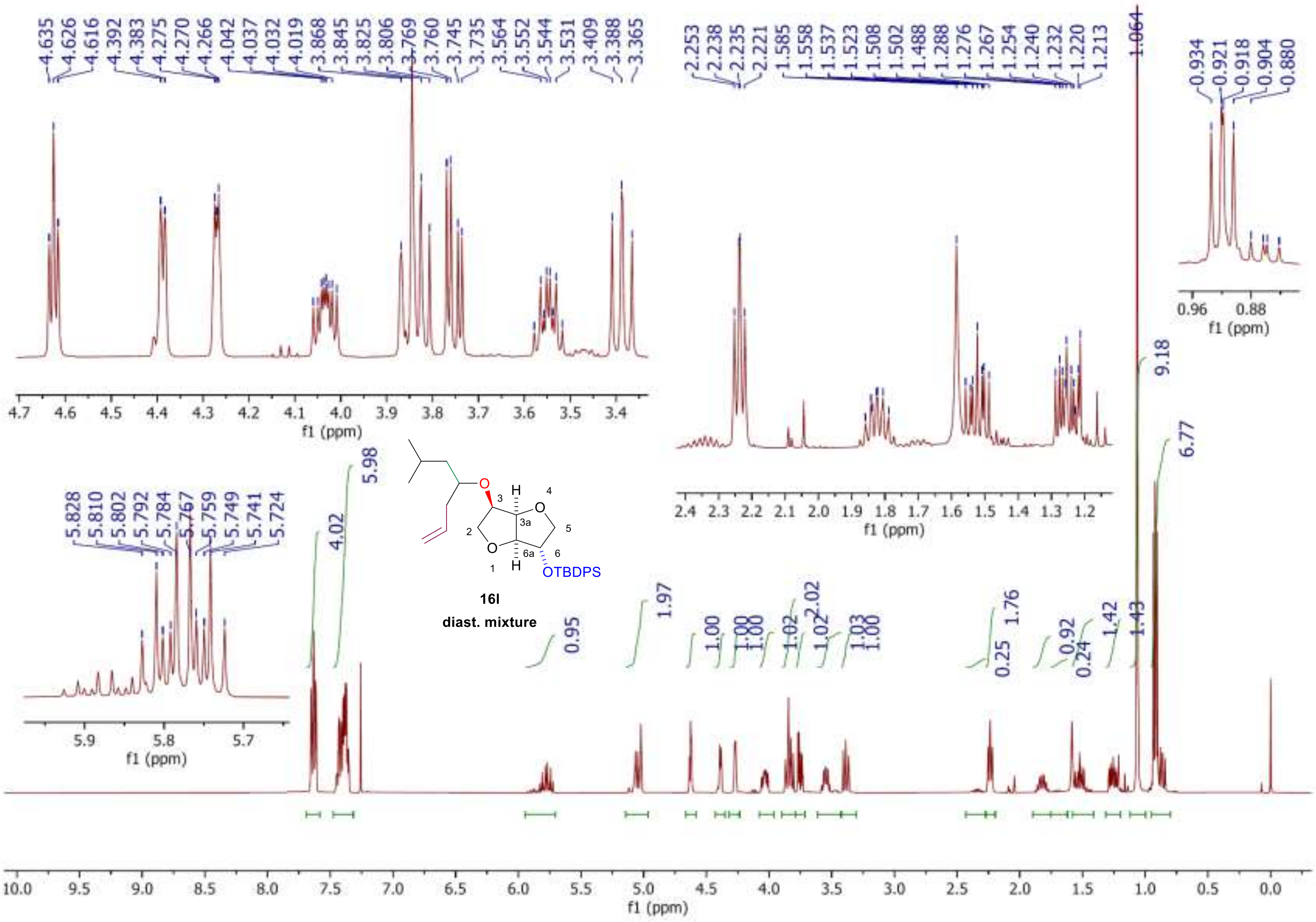
16l
diast. mixture
OTBDPS
f1 (ppm)

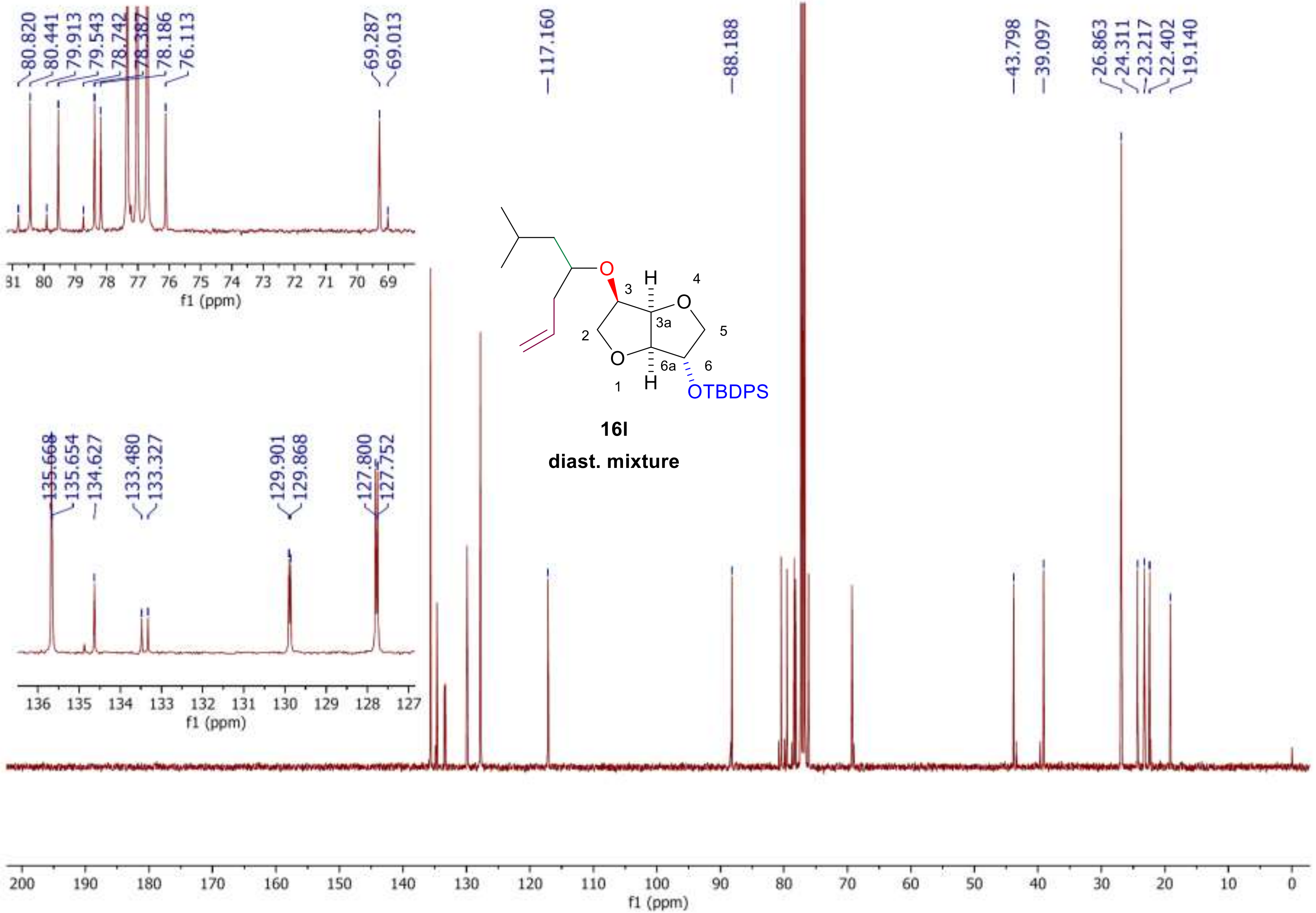
80.820
80.441
79.913
79.543
78.742
78.387
78.186
76.113
69.287
69.013
117.160
88.188
43.798
39.097
26.863
24.311
23.217
22.402
19.140
135.668
135.654
134.627
133.480
133.327
129.901
129.868
127.800
127.752
f1 (ppm)
O
H
O
3
3a
4
5
2
O
6a
6
1
H
OTBDPS
16l
diast. mixture

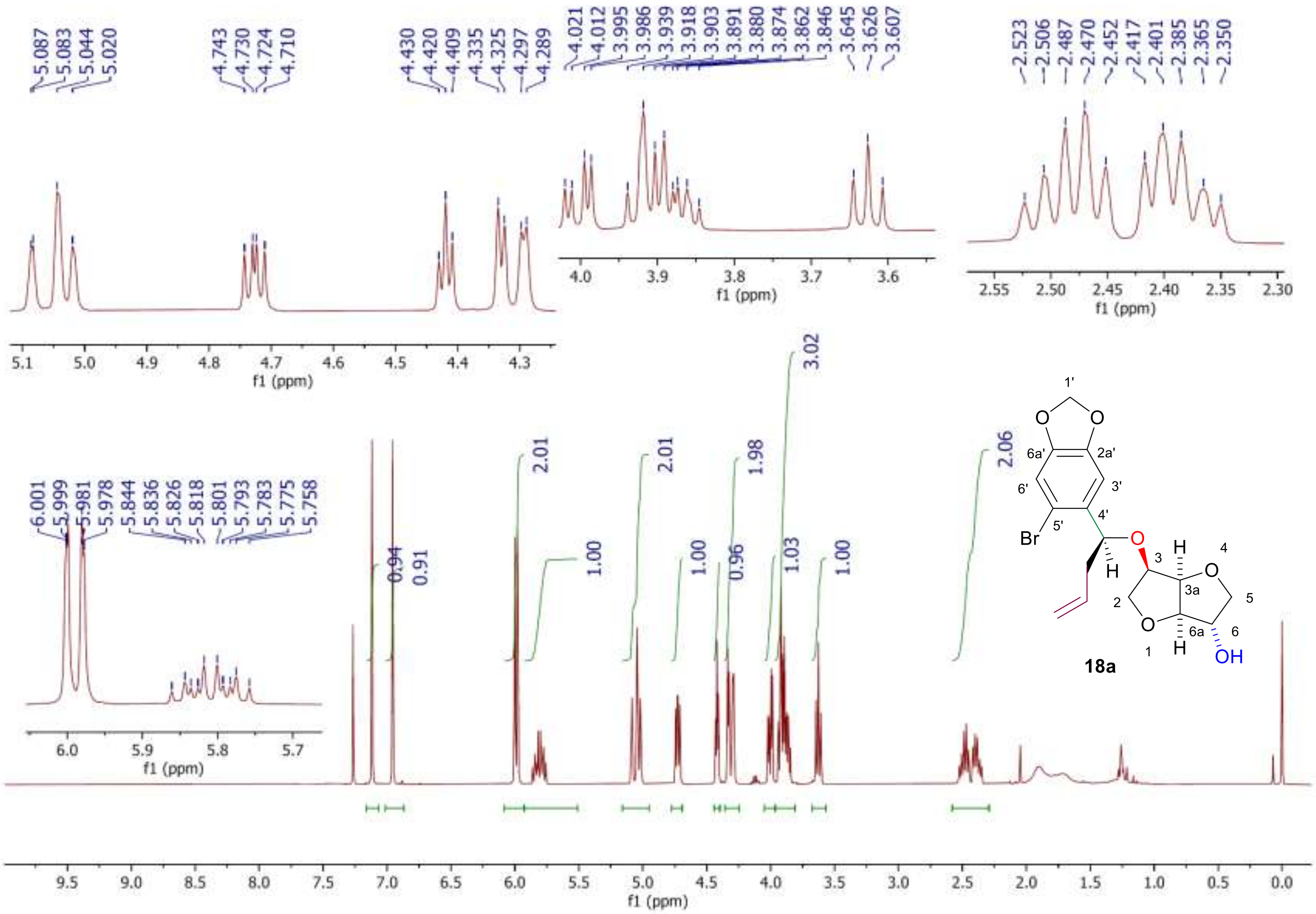
18a
Br
OH
f1 (ppm)

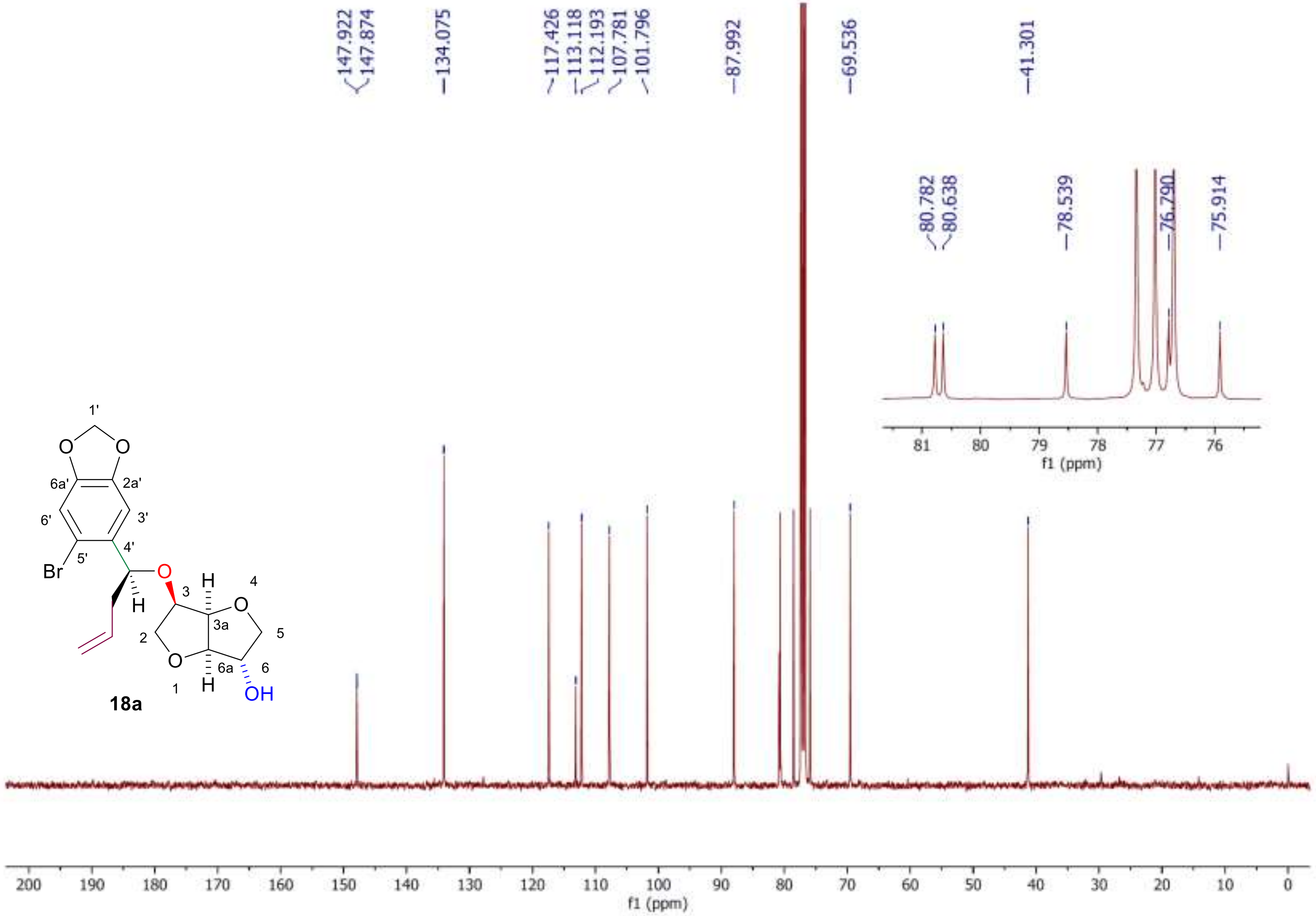
147.922
147.874
134.075
117.426
113.118
112.193
107.781
101.796
87.992
69.536
41.301
80.782
80.638
78.539
76.790
75.914
81
80
79
78
77
76
f1 (ppm)
18a
200
190
180
170
160
150
140
130
120
110
100
90
80
70
60
50
40
30
20
10
0
f1 (ppm)
